# The TUBG meshwork is associated with centromere dynamics and micronuclear organization

**DOI:** 10.1038/s42003-026-10880-y

**Published:** 2026-09-16

**Authors:** Alaaddin Muteb, Martin Johansson, Maria Alvarado-Kristensson

**Affiliations:** 1 Molecular Pathology, Department of Translational Medicine, Lund University, Malmö, Sweden; 2 Department of Laboratory Medicine, Institute of Biomedicine, Sahlgrenska Center for Cancer Research, Sahlgrenska Academy, University of Gothenburg, Gothenburg, Sweden

**Keywords:** Nuclear organization, Molecular medicine

## Abstract

This study investigates how γ-tubulin and the centrosome contribute to interphase centromere dynamics and nuclear organization. Although classically associated with mitotic microtubule nucleation, here we show that γ-tubulin associates with chromatin and is enriched within centromere-defined volumes. Using live-cell imaging, immunofluorescence, and chromatin immunoprecipitation sequencing, we detect γ-tubulin-associated signal at satellite-rich, centromere-proximal chromatin. Reduced γ-tubulin levels are associated with increased centromere fluorescence intensity and reduced mobility, linking the γ-tubulin network to centromere organization. Under acute cisplatin-induced stress, centromere mobility increases, whereas centromere clustering is observed in separate fixed-cell analyses. Ser131 phosphorylation is associated with γ-tubulin self-assembly and centromere-related dynamics. Additionally, γ-tubulin accumulates in micronuclei, coinciding with increased replication-associated signal and DNA fluorescence. In primary clear cell renal cell carcinoma cells, stress is associated with higher γ-tubulin fluorescence intensity within centromere-defined volumes. Together, these findings support an association between the γ-tubulin meshwork and centromere organization, chromatin compartmentalization, and responses to genomic stress.

## Introduction

The centromere, a repetitive satellite DNA region, supports kinetochore formation and chromosome segregation during mitosis and contributes to chromatin organization and genome stability during interphase^1^. Its interphase biology remains poorly understood. Because centromeric DNA is highly repetitive, distinguishing direct chromatin binding from association with centromere-associated protein complexes requires complementary biochemical and imaging approaches.

In acentriolar higher plant cells, TUBG colocalizes with αβ-tubulin dimers in the kinetochore/centromere region^2^, suggesting involvement in kinetochore-microtubule organization. In mammalian cells, TUBG is enriched at centrosomes, microtubule-organizing centers marked by SAS6 and pericentrin, where it is a core component of the γ-tubulin ring complex (γ-TuRC), which is required for mitotic microtubule nucleation.

TUBG also assembles into a higher-order scaffold termed the “TUBG meshwork,” which extends beyond the pericentriolar material to include non-centrosomal γ-strings and γ-tubules^3^. This meshwork links cytoplasmic and nuclear compartments and is associated with nuclear architecture. During interphase, when the nuclear envelope restricts cytosolic microtubule access to centromeres, TUBG associates with chromatin and chromatin-associated structures.

TUBG is associated with nuclear architecture, chromatin organization, nuclear lamina reformation, E2F regulation, PCNA recruitment to replication-associated regions, and DNA damage responses. TUBG depletion affects the organization of microtubules, vimentin, Lamin B, actin, as well as centrosome dynamics, supporting a critical hubbing role for TUBG in cytoskeletal and nuclear organization^4–13^.

Altered TUBG expression and localization occur in gliomas, medulloblastomas, glioblastomas, hepatocellular carcinoma, non-small cell lung cancer, and breast cancer^14–19^. Centrosome amplification and aberrant TUBG expression are associated with genomic instability and poor clinical outcomes, emphasizing the relevance of TUBG-associated structures in cancer biology^20,21^.

Here, we investigate whether the TUBG meshwork is associated with interphase centromere dynamics and nuclear organization. We show that centrosomal TUBG forms a centrosome–nuclear bridge-like structure in which a pericentriolar TUBG mass extends across the nuclear envelope and associates with centromere-defined chromatin regions. Reduced TUBG expression is associated with increased centromere fluorescence intensity and reduced mobility. Ser131 phosphorylation is associated with TUBG meshwork assembly and centromere positioning. Finally, TUBG accumulation in micronuclei is linked to the structural organization of displaced genomic material. Together, these findings support associations among the TUBG meshwork, centromere-associated chromatin, micronuclear organization, and cellular stress responses during interphase.

## Results

### TUBG associates with centromeres and nuclear structures

To distinguish the canonical centrosome from the wider TUBG meshwork, we used molecular and structural criteria. We define the “centrosome” as the dense, SAS6-positive microtubule-organizing center, appearing as a high-intensity TUBG-positive spherical mass. The “TUBG meshwork” encompasses this centrosomal hub and non-centrosomal filamentous γ-strings and γ-tubules. The “centrosome-nuclear bridge” denotes the pericentriolar TUBG mass extending across or into the region defined by nuclear-envelope markers.

**Fig. 1:**
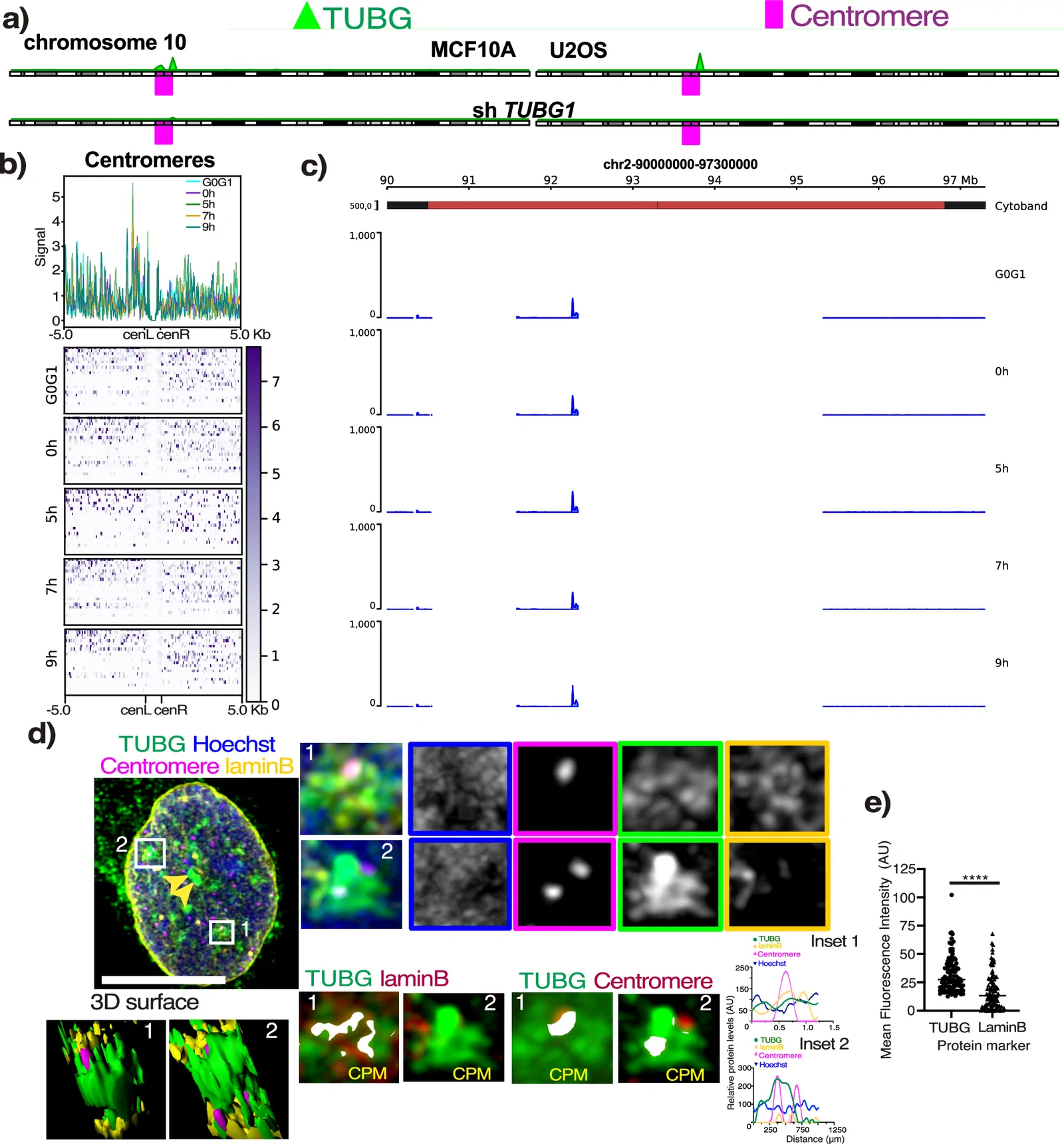
Nuclear localization and centromeric association of TUBG1. **a** Representative genomic tracks showing TUBG ChIP-seq signal distribution across selected chromosomal regions (chromosome 10) in MCF10A cells (GSM2884570_gTub_no_shRNAi_2h) and U2OS cells (GSE173732_TUBG_3h_U2OS). A comparison is shown with cells exhibiting reduced TUBG expression (sh *TUBG*; MCF10A, GSM2884578_gTub_with_shRNAi_2h; U2OS, GSE173732_TUBG_3h_shgtub)^4,22^. **b** Signal-based centromere-centered analysis of normalized TUBG ChIP-seq coverage. Meta-profiles (top) and heatmaps (bottom) show scaled signal across annotated centromere regions (cenL–cenR) with ±5 kb flanking regions in cells synchronized at different cell cycle stages (G0/G1, 0 h, 5 h, 7 h, and 9 h; GSE148798_gTub_G0_G1^4^, GSE148798_gTub_0 h^4^, GSE148798_gTub_5 h^4^, GSE148798_gTub_7 h^4^, and GSE148798_gTub_9 h^4^). The meta-profiles indicate that TUBG-associated signal across centromere regions is broadly comparable among time points, consistent with a stable centromere-associated signal over the analyzed cell cycle stages. Heatmaps further show that the distribution of signal across individual centromeres is likewise largely maintained, although signal intensity varies between centromeres, indicating heterogeneous but recurrent occupancy rather than uniform enrichment across all centromeres. **c** Representative genomic track showing normalized TUBG ChIP-seq signal across a centromere-containing region of chromosome 2 (chr2:90,000,000–97,300,000) in synchronized U2OS cells (G0/G1, 0h, 5h, 7h, and 9h). This panel illustrates the local signal pattern across one representative centromeric region and shows that the overall distribution is comparable across time points. **d** Representative Airyscan 2 images of H9 neural stem cells (NSC). Cells were immunofluorescently co-stained with anti-TUBG (green), anti-centromere (magenta), and anti-Lamin B (yellow); DNA counterstained with Hoechst. To maintain visual clarity, panels display partial maximum intensity projections (MIPs) of Z-stacks (0.1 μm intervals). Yellow arrowheads indicate centrosomes; white boxes delineate magnified insets. Insets highlight nuclear TUBG foci (labeled 1, 2) in close spatial association with centromeres and the nuclear lamina. Bottom right panels show line profiles. 3D surface rendering visualizes the spatial relationship between TUBG, centromeres, and Lamin B. Note that 3D renderings utilize the full Z-stack volume (membrane to membrane), revealing structures outside the 2D projection range (e.g., in inset 2). Colocalization pixel maps (CPM) highlight spatial overlap in white. Scale bars = 10 μm. Images are representative of four independent experiments. **e** 3D quantification of mean fluorescence intensity within centromere volumes. Centromeric volumes were defined by 3D segmentation of anti-centromere signal in interphase nuclei (*n* = 100 centromere-defined volumes in total from *n* = 3 biologically independent experiments). Quantification was performed at the level of individual segmentable centromere-defined volumes rather than by binary positive/negative visual classification of individual centromeres or cells. All segmentable centromeres within the analyzed cells were included when an individual centromere-defined 3D volume could be established. TUBG showed significantly higher fluorescence intensity within centromere-defined volumes than Lamin B, which was quantified using the same centromere-defined ROIs and analysis workflow. Because the displayed maximum-intensity projections collapse the Z-dimension, they do not fully preserve plane-specific spatial relationships. Quantification was therefore performed on the complete Z-stacks using segmented centromere-defined ROIs. Data are presented as mean ± SD from three independent experiments. Statistical significance was determined using a two-sided Wilcoxon matched-pairs signed-rank test (****P < 0.0001), pairing TUBG and Lamin B signals within each individual centromere-defined volume.

To investigate nuclear TUBG, we analyzed publicly available TUBG ChIP-seq datasets from human non-tumorigenic mammary epithelial MCF10A and osteosarcoma U2OS cells^4,22^. Genome-wide peak-density plots showed TUBG enrichment in centromere-associated regions, with reduced enrichment in cells expressing a TUBG1-targeting short hairpin RNA (shRNA) (Fig. 1a, Supplementary Fig. 1a and Table 1).

**Table 1:** Distribution of TUBG-associated peaks relative to centromeres

Sample Id			Distance ranges					
	1	1000	5000	10,000	50,000	1e + 05	5e + 05	1e + 06
GSE148798_gTub_0h	0.01	0.01	0.01	0.01	0.01	0.01	0.02	0.02
GSE148798_gTub_3h	0.01	0.01	0.01	0.01	0.01	0.01	0.01	0.02
GSE148798_gTub_5h	0.01	0.01	0.01	0.01	0.01	0.01	0.02	0.02
GSE148798_gTub_7h	0.01	0.01	0.01	0.01	0.01	0.01	0.01	0.02
GSE148798_gTub_9h	0.02	0.02	0.02	0.02	0.02	0.03	0.05	0.05
GSE148798_gTub_G0_G1	0.01	0.01	0.01	0.01	0.01	0.01	0.02	0.03
GSE173732_TUBG_3h_U2OS	0.30	0.30	0.30	0.33	0.39	0.49	0.64	0.65
GSE173732_TUBG_3h_shgtub	0.00	0.00	0.00	0.00	0.00	0.00	0.06	0.06
GSE173732_TUBG_7h_U2OS	0.14	0.14	0.14	0.16	0.24	0.50	0.67	0.67
GSE173732_TUBG_7h_shgtub	0.00	0.00	0.00	0.00	0.00	0.00	0.00	0.00
GSE173732_TUBG_9h_U2OS	0.33	0.33	0.33	0.35	0.40	0.50	0.67	0.69
GSE173732_TUBG_9h_shgtub	0.08	0.08	0.08	0.08	0.12	0.40	0.56	0.56
GSE173732_TUBG_NS_U2OS	0.17	0.17	0.17	0.19	0.28	0.48	0.62	0.62
GSE173732_TUBG_NS_shgtub	0.00	0.00	0.00	0.00	0.00	0.03	0.03	0.05
GSM2884568_gTub_no_shRNAi_1h	0.37	0.37	0.37	0.37	0.39	0.49	0.59	0.65
GSM2884576_gTub_with_shRNAi_1h	0.34	0.34	0.34	0.34	0.35	0.44	0.54	0.57
GSM2884570_gTub_no_shRNAi_2h	0.39	0.39	0.39	0.40	0.42	0.50	0.65	0.71
GSM2884578_gTub_with_shRNAi_2h	0.23	0.23	0.23	0.23	0.24	0.28	0.33	0.34
Random	0.01	0.01	0.01	0.01	0.01	0.01	0.01	0.2

Values represent the fraction of TUBG-associated ChIP-seq peaks located within each indicated distance from annotated centromeres for each sample. Randomly generated genomic regions are included as a control. Distances are reported in base pairs.

To complement the peak-based analysis and account for signal loss in repetitive centromeric DNA, we performed a centromere-centered analysis of normalized ChIP-seq coverage (Fig. 1b). Meta-profiles showed broadly comparable TUBG occupancy across cell cycle stages, while heatmaps revealed a largely preserved distribution across individual centromeres despite variable signal intensity. Thus, occupancy was heterogeneous but recurrent rather than uniform. This reproducible signal near annotated centromeres does not distinguish direct chromatin association from association through intermediary complexes or broader chromatin domains. Because short-read ChIP-seq has limited mappability within repetitive centromeric cores, the signal there may be underestimated.

RepeatMasker showed that TUBG-associated ChIP-seq signal overlapped with satellite DNA in centromeric and adjacent repetitive regions (Supplementary Fig. 1b). Distances between TUBG-associated peaks and centromeres across cell cycle stages deviated significantly from random expectation (Supplementary Fig. 1c and Table 1), supporting a non-random centromere-proximal association. A chromosome 2 centromeric region showed comparable TUBG signal across G0/G1, 0 h, 5 h, 7 h, and 9 h, consistent with the global analysis (Fig. 1c).

Previous studies have described filamentous and lattice-like TUBG structures, including γ-strings, which associate with chromatin-proximal regions and other cellular compartments^4,6,22–24^. Given these reported associations, we investigated whether TUBG-containing structures are also associated with centromere-defined chromatin regions. To visualize the spatial associations between TUBG and centromeres, we analyzed U2OS and H9-derived neural stem cells (H9-NSC) using a Zeiss LSM 900 equipped with Airyscan 2. Co-immunostaining with anti-TUBG and anti-centromere antibodies revealed close spatial associations among γ-strings, centromeres, and DNA in U2OS cells (Supplementary Fig. 1d). Three-dimensional surface analysis further demonstrated a close spatial association between these structures.

In H9-NSC cells, we additionally observed TUBG-foci, which were previously described and characterized by live-cell imaging in our earlier work^25^, in close spatial association with both Lamin B and centromeres in interphase nuclei (Fig. 1d). To quantify this spatial relationship independently of visual assessment of projected images, we performed automated 3D segmentation and volumetric quantification of fluorescence intensity across the centromere population (Fig. 1e). All segmentable centromeres within the analyzed cells were included when an individual centromere-defined 3D volume could be established. Quantitative analysis of these segmented centromere-defined 3D ROIs yielded continuous measurements of raw TUBG fluorescence intensity within the centromere-defined volumes. Fluorescence intensity was quantified by extracting raw TUBG signal from segmented centromere-defined 3D ROIs and comparing these values with Lamin B fluorescence measured within the same ROIs. The 3D ROI-based quantification of TUBG fluorescence was applied across the cell types analyzed in this study, with 100 centromere-defined volumes analyzed per cell type or experimental group in the relevant analyses, whereas the paired comparison with Lamin B was performed only for Fig. 1e. No binary threshold was used to classify individual centromeres as TUBG-positive or TUBG-negative. For the paired analysis shown in Fig. 1e, TUBG exhibited significantly higher fluorescence intensity than Lamin B across 100 centromere-defined volumes in total from three biologically independent H9-NSC experiments (31.53 ± 15.86 vs. 17.62 ± 15.81 A.U.; *n* = 100 centromeres per cell type; *P* < 0.0001; Wilcoxon matched-pairs signed-rank test; Fig. 1e).

Together, these findings demonstrate recurrent TUBG-associated signal near centromere-proximal chromatin and within centromere-defined volumes, with fluorescence intensity varying among individual centromeres.

### TUBG localization and its association with centromere-associated phenotypes

**Fig. 2:**
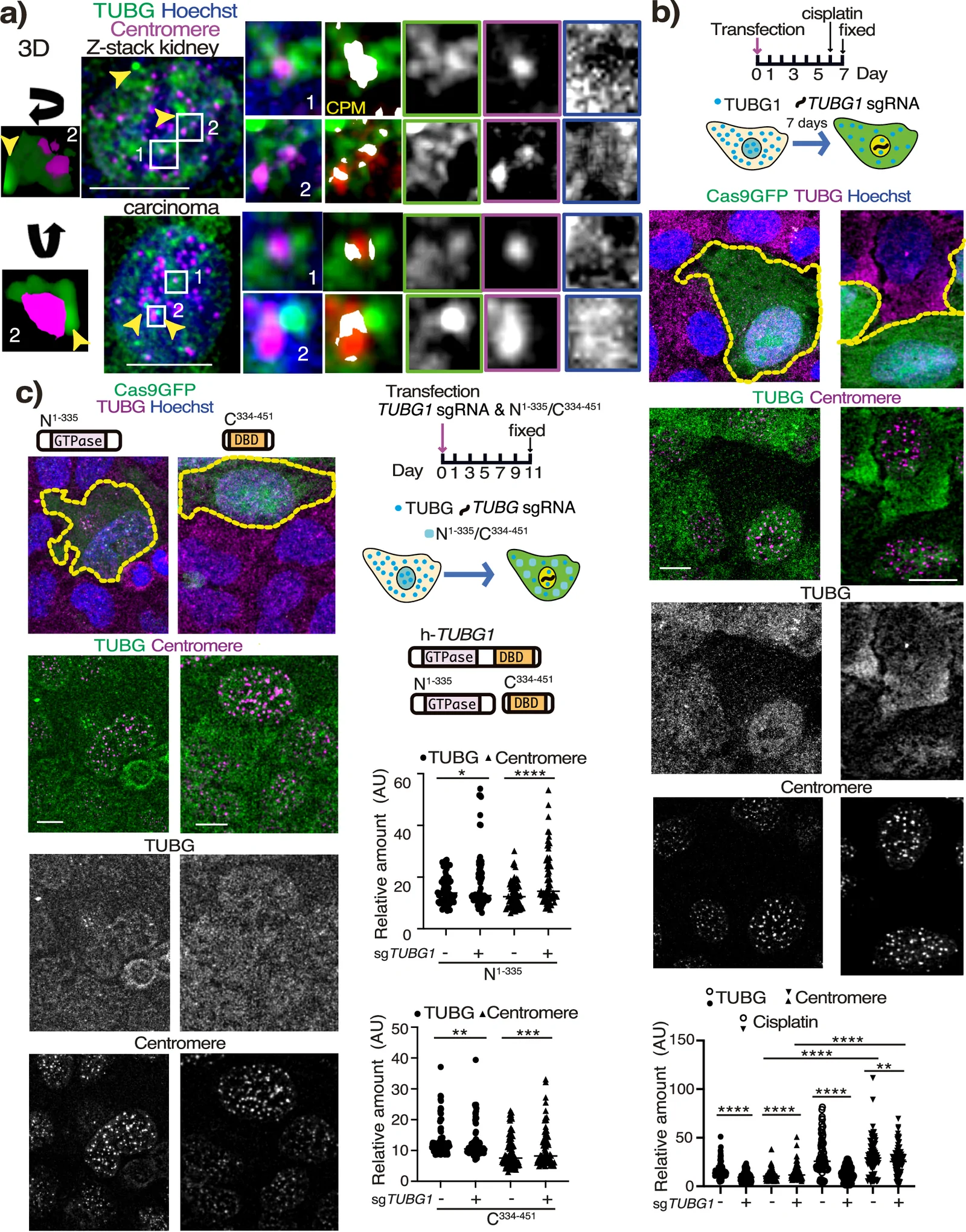
TUBG localization and its association with centromere-associated phenotypes. **a** TUBG localization and spatial association with centromeres in human kidney cells. Representative Z-stack maximum intensity projections (MIPs) of fixed primary human normal kidney epithelial (upper panels) and clear cell renal cell carcinoma (ccRCC) cells (lower panels) acquired with a Nikon A1RHD microscope at 0.1-μm Z-intervals. Cells were immunofluorescently co-stained for TUBG, centromeres, and DNA (Hoechst). Yellow arrowheads indicate centrosomes; white boxes denote magnified regions shown in the insets. Cropped image stacks highlight nuclear TUBG foci (labeled 1, 2) in close spatial association with centromeres. Notably, inset 2 provides a 3D volume-rendered view (left) capturing the specific centrosome in closest proximity to the centromeres in focus. The colocalization pixel map (CPM) was generated from the original red centromere and green TUBG channels using the ImageJ colocalization plugin; colocalized pixels are displayed in white. The red signal in CPM panels therefore reflects the analytical channel assignment used for colocalization mapping and is distinct from the magenta centromere pseudocolor used in the merged presentation images. Scale bars: 10 μm. Images are representative of four independent experiments. **b**, **c** Experimental design and quantitative analysis of association between TUBG expression and centromere-associated phenotypes in U2OS cells. U2OS cells were analyzed under various conditions to assess the effect of TUBG expression on centromeres. A schematic timeline of the experimental procedure is included for context. Fixed cells were immunostained with anti-TUBG and anti-centromere antibodies, with DNA counterstained using Hoechst. Whole-cell confocal images were captured, and Z-stacks were processed into MIPs. Cells expressing Cas9-GFP-*TUBG1*-sgRNA (Cas9-GFP; green) are outlined by yellow dashed lines. Scale bars: 10 μm. Graphs show fluorescence intensity of TUBG and centromeres measured using ImageJ. Symbols identify the experimental groups as indicated in the graph key. Lines connect paired measurements obtained from cells analyzed within the same imaging field. Filled and open symbols indicate the treatment status shown in the graph key. Paired comparisons were analyzed using two-sided Wilcoxon matched-pairs signed-rank tests. Comparisons between independent treatment groups were analyzed using two-sided Mann–Whitney *U* tests. The Wilcoxon matched-pairs signed-rank test was used for paired comparisons because matched cells were analyzed within the same imaging fields and the data did not satisfy normality assumptions by Shapiro–Wilk testing. Independent treatment comparisons were analyzed using the Mann–Whitney *U* test. **b** Quantitative analysis of TUBG and centromere fluorescence intensity in TUBG1-deficient cells. Cells expressing Cas9-GFP-*TUBG1*-sgRNA (*n* = 100 cells) were compared with adjacent Cas9-GFP-*TUBG1*-sgRNA-non-expressing control cells (*n* = 100) within the same imaging fields to provide an internal experimental control. To investigate the combined effect of genetic depletion and chemical stress, cells were treated with 5 μM cisplatin for 24 h prior to fixation (*n* = 84 for both sg-expressing and non-expressing populations). The graph shows that genetic depletion of *TUBG1* was associated with significantly higher centromere fluorescence intensity compared with adjacent non-expressing control cells (****P < 0.0001). Cisplatin treatment was also associated with significantly higher centromere fluorescence intensity in both non-expressing control cells (****P < 0.0001) and sgTUBG1-expressing cells (**P = 0.0071) compared with their respective untreated controls. A direct paired comparison of the cisplatin-treated populations showed significantly higher centromere fluorescence intensity in non-expressing control cells than in neighboring sgTUBG1-expressing cells (***P < 0.001, a two-sided Wilcoxon matched-pairs signed-rank test). This result indicates that the centromere-associated response to cisplatin differed between cells with normal and reduced TUBG levels. Statistical significance for comparisons between matched populations within the same field of view was determined by a Wilcoxon matched-pairs signed-rank test, effectively controlling for local variations in immunofluorescence intensity and background noise. Comparisons between independent untreated and cisplatin-treated groups were performed using the Mann–Whitney *U* test. Data are presented as mean ± SD from five independent experiments. **c** Quantitative analysis of TUBG and centromere fluorescence intensity in cells expressing *TUBG1*-sgRNA-resistant constructs. Fluorescence intensity in control cells expressing only *TUBG*^1–335^ (N^1–335^; *n* = 63 cells) or *TUBG*^334-451^ (C^334-451^; *n* = 101 cells) was compared to cells co-expressing Cas9-GFP-*TUBG1*-sgRNA with the respective resistant constructs (mean ± SD, *n* = 63 and *n* = 101, respectively; *n* = 3 biologically independent experiments). Statistical significance is indicated as *P = 0.0476, **P = 0.0018, ***P < 0.001, ****P < 0.0001 (two-sided Wilcoxon matched-pairs signed-rank tests).

To assess whether the TUBG–centromere spatial association was also observed in primary human renal cells, we analyzed primary human kidney epithelial cells and clear cell renal cell carcinoma (ccRCC) cells (Fig. 2a). We performed automated 3D segmentation and volumetric quantification of fluorescence intensity using the 3D ImageJ Suite^26^ to quantify TUBG fluorescence within centromere-defined volumes. This analysis yielded continuous raw TUBG fluorescence-intensity measurements across the analyzed centromere-defined volumes (*n* = 100 centromeres per group) in both normal (25.15 ± 8.66 A.U.) and cancerous renal cells (15.08 ± 5.02 A.U.). No binary threshold was used to classify individual centromeres as TUBG-positive or TUBG-negative. These observations across the analyzed cell types support a recurrent spatial association between TUBG fluorescence and centromere-defined regions (Fig. 2a).

Having observed TUBG fluorescence within centromere-defined volumes in these renal cell preparations, we next examined the spatial distribution of these regions within the cell. We observed that centromere-defined regions associated with TUBG signal were frequently located in close proximity to centrosomes (24 ± 7% in normal kidney vs. 15 ± 4% in ccRCC cells; *n* = 400 centrosomes per group). This spatial distribution supports an association between nuclear TUBG- and centromere-associated regions and the cytoplasmic centrosomes (Fig. 2a).

To test whether reduced TUBG levels are associated with changes in centromere-associated phenotypes, we used Cas9-GFP-*TUBG1-*sgRNA to deplete TUBG expression in U2OS cells (Fig. 2b). To ensure the rigor of this perturbation analysis, we verified the depletion of TUBG protein levels in every analyzed GFP-positive cell. Quantitative analysis revealed that depletion of TUBG1 significantly increased centromere fluorescence intensity compared with non-expressing control cells. These findings show that reduced TUBG levels are associated with increased centromere fluorescence intensity. However, the fluorescence-intensity measurements do not establish altered centromere function, chromatin compaction, chromatin condensation, or altered centromere volume.

We next tested whether the increased centromere fluorescence intensity was accompanied by altered centromere dynamics. Using 4D live-cell imaging of mCherry-CENP-A and Cas9-GFP-*TUBG1-*sgRNA-expressing cells, we quantified centromere kinematics over 49-minute intervals. The increased centromere fluorescence intensity was accompanied by reduced mobility: mean track speed decreased from 1.31 ± 0.78 µm/min in control cells (*n* = 30 tracks) to 0.36 ± 0.92 µm/min upon TUBG depletion (*n* = 26 tracks). This group-level association does not establish that the fluorescence-intensity change directly caused the reduction in centromere mobility.

To determine whether these centromere-associated measurements changed in response to genotoxic stress, we treated cells with 5 μM cisplatin for 24 h, a DNA-crosslinking agent^27^. Cisplatin treatment was associated with a significant increase in centromere fluorescence intensity in control cells (****P < 0.0001) and in sg*TUBG1*-expressing cells (****P < 0.0001; Fig. 2b). A direct paired comparison within the cisplatin-treated cultures showed significantly higher centromere fluorescence intensity in non-expressing control cells than in neighboring sgTUBG1-expressing cells (*P* < 0.01; Fig. 2b). This comparison indicates that the centromere-associated response to cisplatin differed between cells with normal and reduced TUBG levels.

To resolve the acute effects of stress on 4D dynamics, we performed paired time-lapse imaging of cells in which TUBG expression was restored using an shRNA-resistant add-back construct (Supplementary Fig. 2). While baseline speed was 0.64 ± 0.63 µm/min (*n* = 43 tracks), mobility significantly increased to 1.03 ± 0.78 µm/min during the 50–99-min window after cisplatin addition (*n* = 63 tracks)^28^. These data show increased centromere mobility following acute cisplatin exposure in the TUBG add-back model. Separately, fixed-cell analyses showed increased centromere clustering after cisplatin treatment. Because the mobility and clustering observations were obtained from distinct live- and fixed-cell datasets, respectively, they do not establish a temporal sequence or causal relationship between these phenotypes. Together, the findings support associations among TUBG levels, centromere fluorescence intensity, centromere mobility, and the response to cisplatin without establishing direct causal relationships.

Finally, we investigated whether specific TUBG structural domains were sufficient to reverse the measured phenotype. We assessed whether the N-terminal fragment (TUBG^1-335^, containing the GTPase domain^6,11,22^) or the C-terminal fragment (TUBG^334-451^, containing the nuclear localization and DNA-binding domains^6,11,22^) could restore the measured centromere-associated phenotypes (Fig. 2c). While previous work showed that full-length sg-resistant TUBG1 could rescue cell survival, neither the N-terminal fragment nor the C-terminal fragment fully reversed the centromere intensity changes induced by Cas9-GFP-*TUBG1-*sgRNA^6,11,22,23,25^. These findings indicate that neither fragment alone was sufficient to restore the measured phenotype and are consistent with a requirement for full-length TUBG in this assay. They do not establish the underlying mechanism or demonstrate altered centromere function.

### Centrosome-associated TUBG forms a cytosol-nuclear chromatin-proximal structure

**Fig. 3:**
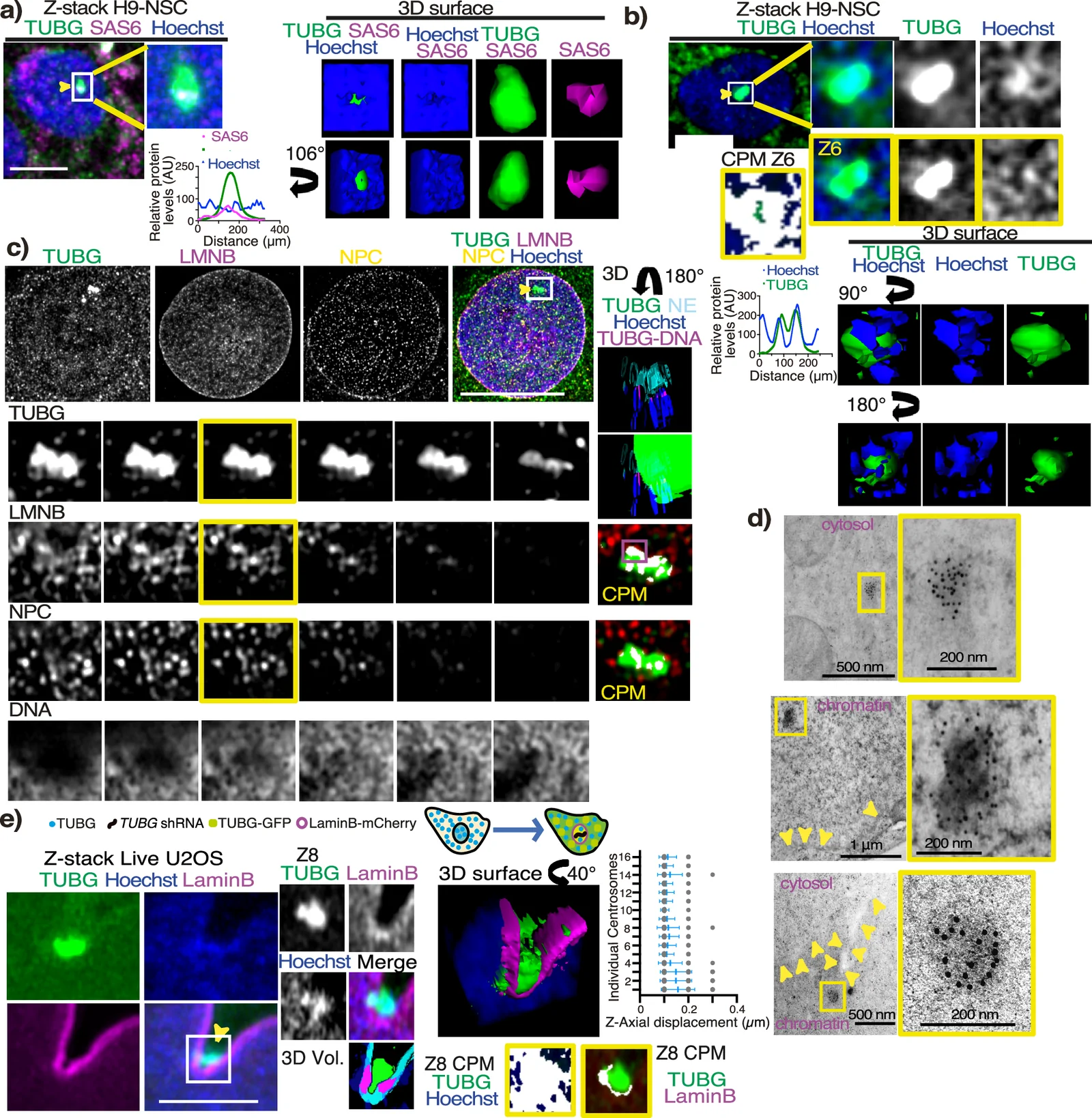
Centrosomal TUBG extends toward the nuclear interior and is associated with chromatin-proximal regions. **a**–**c** Centrosomal TUBG extends across the boundary defined by nuclear envelope (NE) markers and is spatially associated with DNA and the nuclear lamina in human neural stem cells (H9-NSC) and osteosarcoma cells. **a**, **b** Confocal Z-stack maximum intensity projections (MIPs) and line profiles of H9-NSCs (acquired at 0.4-μm intervals) demonstrating pericentriolar material (PCM) extending into the nuclear volume and showing close spatial association with chromatin. Insets in **b** show magnified MIPs and a single Z-slice (Z6) highlighting the spatial association between the PCM and chromatin. **c** High-resolution Airyscan 2 imaging (acquired at 0.1-μm intervals) of a U2OS cell labeled for endogenous TUBG (centrosome), LMNB/NPC (nuclear envelope), and Hoechst (DNA). For 3D volumetric analysis, the magenta box indicates the region of interest (ROI) processed for 3D surface reconstruction; note that this reconstruction captures a centrosomal doublet in close proximity to the NE. In the upper 3D snapshot, the NE boundary (cyan) was defined by a Boolean OR operation of LMNB and NPC signals to ensure that discontinuities in either individual marker did not produce gaps in the segmented boundary, providing a conservative estimate of TUBG signal located within the segmented nuclear volume. DNA is rendered in blue. The specific volume of TUBG-DNA spatial overlap/proximity is highlighted in magenta, representing the internal centrosomal fraction that overlaps with or is closely apposed to chromatin. In the lower 3D snapshot, the total centrosomal TUBG volume is added in green to visualize the spatial orientation of the cytosolic centrosome relative to the NE-associated TUBG extension. 3D surfaces were rotated to provide an optimal perspective of the spatial relationship. Insets in **c** show magnified sequential Z-slices illustrating the distribution of TUBG relative to the NE boundary. Colocalization pixel maps (CPM) for the magnified regions (yellow plane) indicate overlapping signals (white) between TUBG (green), DNA (blue), and the NE (red: NPC/LMNB). As in Fig. 2, CPM panels represent analytical colocalization outputs generated from the original channel assignments; white pixels indicate spatial overlap between the indicated channels. **d** Endogenous TUBG localizes within the nucleus by immunoelectron microscopy. Detection of endogenous TUBG in U2OS cells using high-pressure freezing and immunogold labeling (anti-TUBG; gold-conjugated protein A). Yellow arrowheads indicate the nuclear envelope; yellow boxes highlight immunolabeled TUBG structures in the cytosol and in close association with nuclear chromatin. **e** Live-cell imaging of TUBG-associated centrosome-nuclear spatial relationships. The schematic illustrates stable expression of TUBG-GFP (shRNA-resistant) in *TUBG-*shRNA cells (to reduce endogenous TUBG), coexpressing Lamin B-mCherry. Live U2OS cells were imaged with Airyscan microscopy (0.1-μm intervals) and displayed as MIPs. DNA was stained with Hoechst. Insets highlight the eighth Z-slice (Z8) within the white box, illustrating the centrosomal TUBG extending across or remaining closely apposed to the Lamin B-defined nuclear boundary. For 3D Volumetric analysis, surfaces were generated to optimize visualization of the cytoplasmic-nuclear chromatin-proximal arrangement. In the 3D volume (3D Vol.) snapshot, the NE boundary is rendered in cyan (Lamin B-mCherry) and the DNA in blue. The specific volume of TUBG–DNA spatial overlap/proximity is rendered in magenta, representing the internal centrosomal fraction overlapping with or closely apposed to chromatin. The centrosomal TUBG volume remaining outside the nucleus is added in green to provide the spatial context. Yellow arrowheads denote the centrosome, while the yellow insets display CPMs, showing spatial overlap between Lamin B (magenta) and TUBG (green) or DNA (blue) with TUBG (green) channels within the magnified region of the white box, where colocalization between specified channels is shown as white pixels. The accompanying graph in e shows the quantitative analysis of centrosome–nuclear-envelope dynamics. Distribution of active Z-axial displacements (> 0.1 μm) for individual centrosomes. Gray dots represent individual frame-to-frame movements (*n* = 1050 frames); blue symbols represent the mean active step for each centrosome (*n* = 16 centrosomes from 12 cells). Movements below 0.1 μm, corresponding to the Z-sampling interval and operationally defined as a “stable state,” were maintained for 46.86 ± 11.98% of the observation period and were excluded from this plot to highlight active repositioning dynamics. The population mean active step was 0.1236 ± 0.0712 μm. Error bars indicate ± SD. Scale bars: 10 μm (**a**–**c**); 500 nm in the upper and lower overview images, 1 μm in the middle overview image, and 200 nm in the magnified insets in (**d**); 5 μm in (**e**). Images are representative of four independent experiments.

To gain deeper insights into the spatial organization of centrosome-associated TUBG, we examined H9-NSC and U2OS osteosarcoma cells. These analyses revealed a strong spatial association between centrosomes and the nuclear envelope. Co-immunostaining with anti-TUBG and anti-SAS6 (a centrosome marker) antibodies showed that 69.8% ± 16.4% of centrosomes in undifferentiated H9-NSCs were closely apposed to or spatially overlapping with the nuclear envelope (*n* = 3 experiments, 600 cells; ***P < 0.001) (Fig. 3a). Importantly, TUBG immunofluorescence demonstrated that pericentriolar TUBG was closely associated with nuclear chromatin (Fig. 3b) and was also associated with Lamin B and nuclear pore complexes (Fig. 3c), consistent with the presence of TUBG signal at the nuclear periphery and within the region bounded by the nuclear-envelope markers.

To quantify the extent of TUBG distribution relative to the nuclear boundary, we performed 3D volumetric reconstruction and Boolean intersection analysis of two Airyscan datasets (Fig. 3c). In a representative fixed U2OS cell exhibiting a complex centrosomal arrangement (comprising two adjacent centrosomes in Fig. 3c), the total centrosomal TUBG volume was 6.431 μm^3^. Of this volume, 4.003 μm^3^ overlapped with the segmented nuclear interior, corresponding to 62.25% of the total segmented centrosomal TUBG volume. Within this internal pool, 0.183 μm^3^ of TUBG volume spatially overlapped with or was closely apposed to Hoechst-stained chromatin. These measurements show that centrosomal TUBG signal extends across the nuclear-envelope boundary and includes a discrete chromatin-proximal fraction. The resulting TUBG–DNA overlap/proximity index of 0.046 indicates that a small proportion of the internal TUBG volume spatially overlapped with or was closely apposed to Hoechst-stained chromatin.

Complementary ultrastructural evidence was obtained by immunoelectron microscopy of high-pressure-frozen U2OS cells. This analysis revealed TUBG-labeled regions closely associated with chromatin-dense areas (Fig. 3d), consistent with the immunostaining patterns and with the localization of TUBG within the pericentriolar matrix and at chromatin-proximal sites.

Airyscan live-cell imaging of U2OS cells stably expressing *TUBG*-shRNA, sh-resistant TUBG-GFP (sh*TUBG*-TUBG; Supplementary Fig. 2), and Lamin B-mCherry (sh*TUBG*-TUBG-LMNB-U2OS) showed that centrosome-associated TUBG extended from the cytoplasm across or into the Lamin B-defined nuclear boundary and remained closely apposed to DNA (Fig. 3e). Volumetric analysis of these live-cell dynamics revealed a total TUBG volume of 2.861 μm^3^, of which 0.57 μm^3^ overlapped with the segmented nuclear interior (19.92%). The lower total volume compared to fixed-cell measurements likely reflects the analysis of a single centrosome and the potential underestimation of the total pericentriolar matrix by the TUBG-GFP reporter. The internal pool included a TUBG–DNA spatial overlap/proximity volume of 0.439 μm^3^, resulting in a TUBG–DNA overlap/proximity index of 0.77. Thus, in this representative live-cell dataset, a substantial fraction of the internal centrosome-associated TUBG signal overlapped with or was closely apposed to chromatin.

Together, these findings indicate that centrosomes may provide a structural interface through which TUBG-associated assemblies are positioned near nuclear chromatin.

### Dynamic centrosome–nuclear spatial associations characterize TUBG positioning near nuclear structures

Given the observed spatial association of pericentriolar TUBG with nuclear components, we next investigated the dynamic nature of these spatial relationships. Our aim was to characterize centrosome movement near the nuclear envelope and determine how TUBG-associated structures are positioned relative to nuclear components.

We first performed time-lapse imaging of sh*TUBG*-TUBG U2OS cells stably expressing mCherry-NUP50-N10, captured with Airyscan 2 microscopy. These experiments revealed dynamic centrosome behavior. Specifically, the centrosome was observed moving out of the imaging plane toward the nuclear envelope (labeled with mCherry-NUP50 and Hoechst), subsequently reappearing in the plane within minutes (Supplementary Fig. 3a), demonstrating centrosome mobility near the nuclear envelope.

To further examine these dynamic spatial associations between the pericentriolar matrix and the inner nuclear-envelope marker Lamin B, we captured Z-stack time-lapse images of sh*TUBG*-TUBG-LMNB-U2OS cells. These Z-stacks revealed transient spatial associations of the pericentriolar matrix (TUBG-GFP) with the nuclear envelope (Lamin B-mCherry; Supplementary Fig. 3b). To quantify these dynamics, we tracked axial (*Z* axis) displacement over time. Despite this mobility, the centrosome–nuclear interface exhibited substantial positional stability, remaining in the operationally defined stable state (displacement < 0.1 μm) for 46.86% ± 11.98% of the total observation period. When active repositioning occurred (defined as displacement > 0.1 μm), the centrosome moved with a mean active step of 0.1236 ± 0.0712 μm (Fig. 3e: *n* = 1050 frames, 12 cells, 16 centrosomes). Over the course of the imaging sessions, each centrosome traversed an average cumulative distance of 4.42 ± 1.69 μm, representing a continuous but spatially constrained exploration of the nuclear periphery. To assess whether these associations reflected reproducible spatial relationships rather than stochastic overlap or technically induced stage drift, we performed 3D volumetric colocalization analysis on frames capturing peak nuclear proximity (Supplementary Fig. 3b). In these sampled frames, the thresholded Manders’ Coefficient (*M*_*2*_) reached 1.0, with a mean colocalized volume of 90.03% ± 7.42%, supporting a high degree of spatial overlap under the analyzed conditions.

Collectively, these dynamic observations—demonstrating not only the extension of pericentriolar TUBG into the segmented nuclear region, together with centrosome movement and transient spatial associations with nuclear-envelope components—are consistent with centrosome mobility contributing to the positioning of cytosolic TUBG-associated structures near centromeres and other nuclear structures, without establishing direct physical continuity or mechanistic control.

### Dynamic TUBG-associated structures show spatial associations with interphase centromeres

Building upon our previous observation of TUBG association with centromeres and localization at the centrosome–nuclear interface, we next investigated the real-time spatial relationships between the TUBG meshwork and centromeres during interphase. This network includes γ-strings and γ-tubules, cytosolic TUBG-containing fibers with reported diameters of ~20–25 nm^23^. Together, these structures form a TUBG network spanning the cytoplasm, mitochondria, and the nuclear compartment, where TUBG has been associated with cell polarization, proliferation, and homeostasis, centrosome positioning, and cellular organization through the distribution of TUBG1 among centrosomes, γ-strings, and γ-tubules^3,4,12,22,29^.

**Fig. 4:**
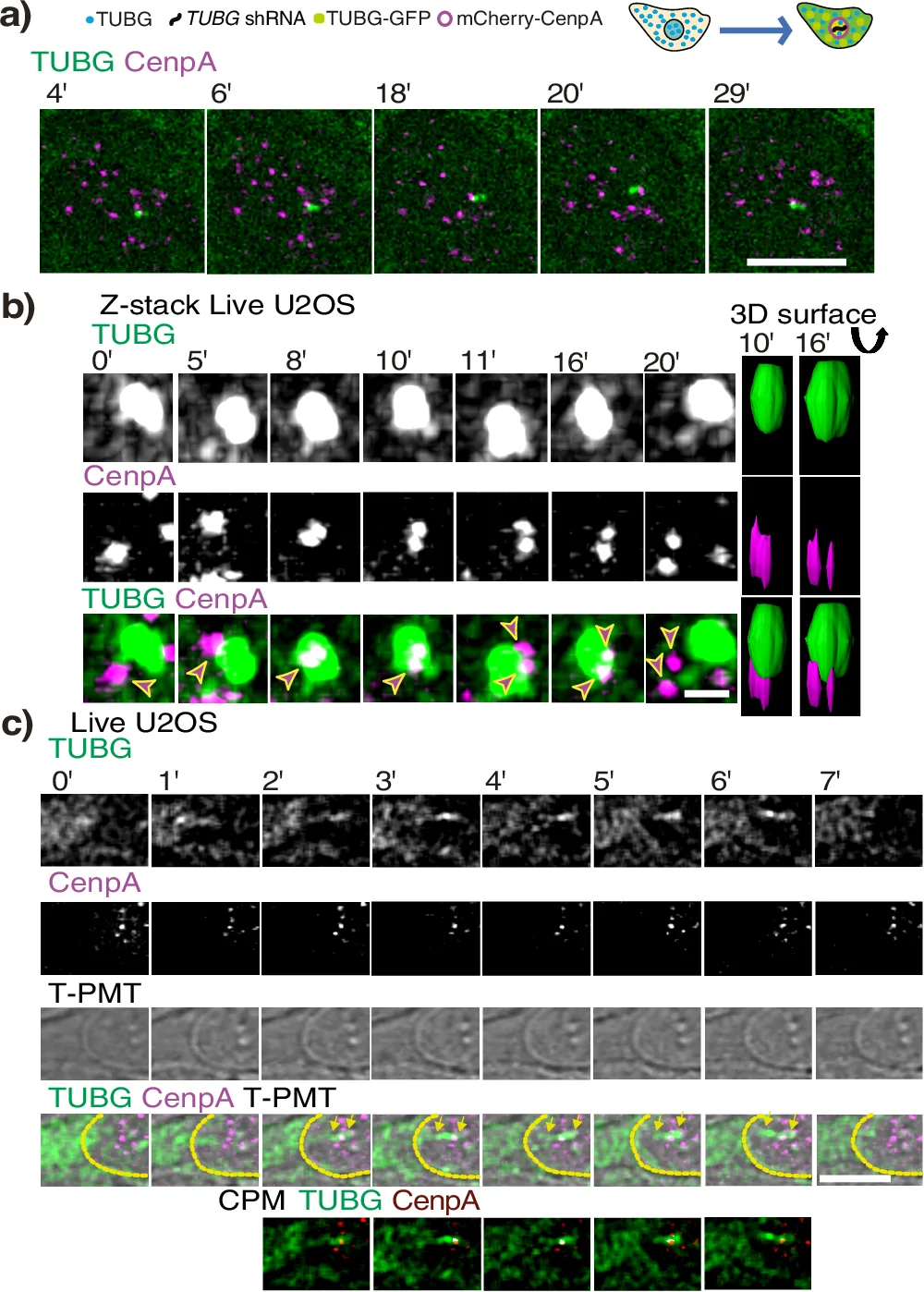
Centrosome–centromere dynamics and γ-tubule formation. **a**–**c** Live-cell imaging of TUBG-associated centrosome–centromere dynamics and γ-tubule formation. Live U2OS cells stably expressing *TUBG* shRNA, sh-resistant TUBG-GFP, and mCherry-CENP-A (a centromere marker) were imaged using a confocal microscope. Time-lapse Z-stack images were acquired at 1-minute intervals (with 1-μm intervals between Z-planes) and displayed as either maximum intensity projections (MIPs; **a**, **b**) or single Z-slices (**c**). The time points shown in the time-lapse sequences correspond to those in Supplementary Videos 1–3 (*n* = 200 cells from four independent experiments). A schematic representation of the modifications made to the cell lines is included for context. **a** Time-lapse sequence illustrating the dynamic movement of centrosomal TUBG (green) in close spatial association with centromeres (magenta) at the nuclear surface. Scale bars: 10 μm. **b** Time-lapse sequence showing a close spatial association between centrosomal TUBG (green) and a single centromeric focus (magenta), followed by the apparent separation of the centromeric signal into two foci. 3D surface reconstructions generated using the ImageJ 3D Viewer plugin were rotated to optimize visualization of centrosome–centromere spatial associations. Yellow and magenta arrowheads indicate the resulting two centromeric foci. Scale bars: 1 μm. **c** Transmitted photomultiplier tube (T-PMT)/fluorescence time-lapse images show the formation of a γ-tubule (TUBG, green) extending from the nuclear envelope toward CENP-A-labeled centromeric foci (magenta). Colocalization pixel maps (CPM), where white areas indicate colocalized pixels across channels, highlight spatial overlap between CENP-A and TUBG signals. Scale bars: 10 μm.

We performed live-cell imaging of sh*TUBG*-TUBG U2OS cells stably expressing the centromeric protein CENP-A tagged with mCherry (sh*TUBG*-TUBG-CENP-A-U2OS). These analyses revealed that the centrosome maintains a persistent proximity to the nuclear periphery and is spatially associated with a specific subset of centromeric foci (Fig. 4a). In some instances, centrosomal proximity temporally coincided with changes in the spatial distribution of centromeric foci (Fig. 4b and Supplementary Video 2). This temporal co-occurrence is consistent with an association between the TUBG-containing centrosomal region and centromere-associated structural behavior during interphase, but does not establish that TUBG directly causes centromere reorganization.

Further live-cell observations showed TUBG accumulation at the nuclear membrane, followed by the formation of filamentous γ-tubules that extended into the nuclear interior^3^. Notably, these γ-tubules formed transient spatial associations with specific centromeres (Fig. 4c and Supplementary Video 3), demonstrating a reproducible spatial relationship between dynamic TUBG-containing structures and a subset of centromeric foci.

Taken together, these results provide live-cell evidence that TUBG accumulation near centromere-associated regions, together with γ-tubule formation, is associated with the spatial behavior of a specific centromere subpopulation during interphase. These observations suggest that the centrosome–nuclear interface may position TUBG-associated structures near nuclear compartments and that the meshwork assembly state is associated with these spatial relationships. The present observations do not establish a direct physical interaction or causal control of centromere organization by TUBG.

### The TUBG meshwork is associated with centromere positioning under stress conditions

Expanding on our characterization of centrosome-associated TUBG near nuclear structures, we further investigated spatial associations between the broader TUBG meshwork and centromeres under cellular stress conditions. Given the pivotal role of TUBG in the brain and the occurrence of centrosome amplification in cancer, we hypothesized that cellular stress might alter the spatial relationship between TUBG-containing structures and centromeres. We therefore examined whether centromeres were positioned near centrosomal and non-centrosomal TUBG-containing structures under oxidative and genotoxic stress.

**Fig. 5:**
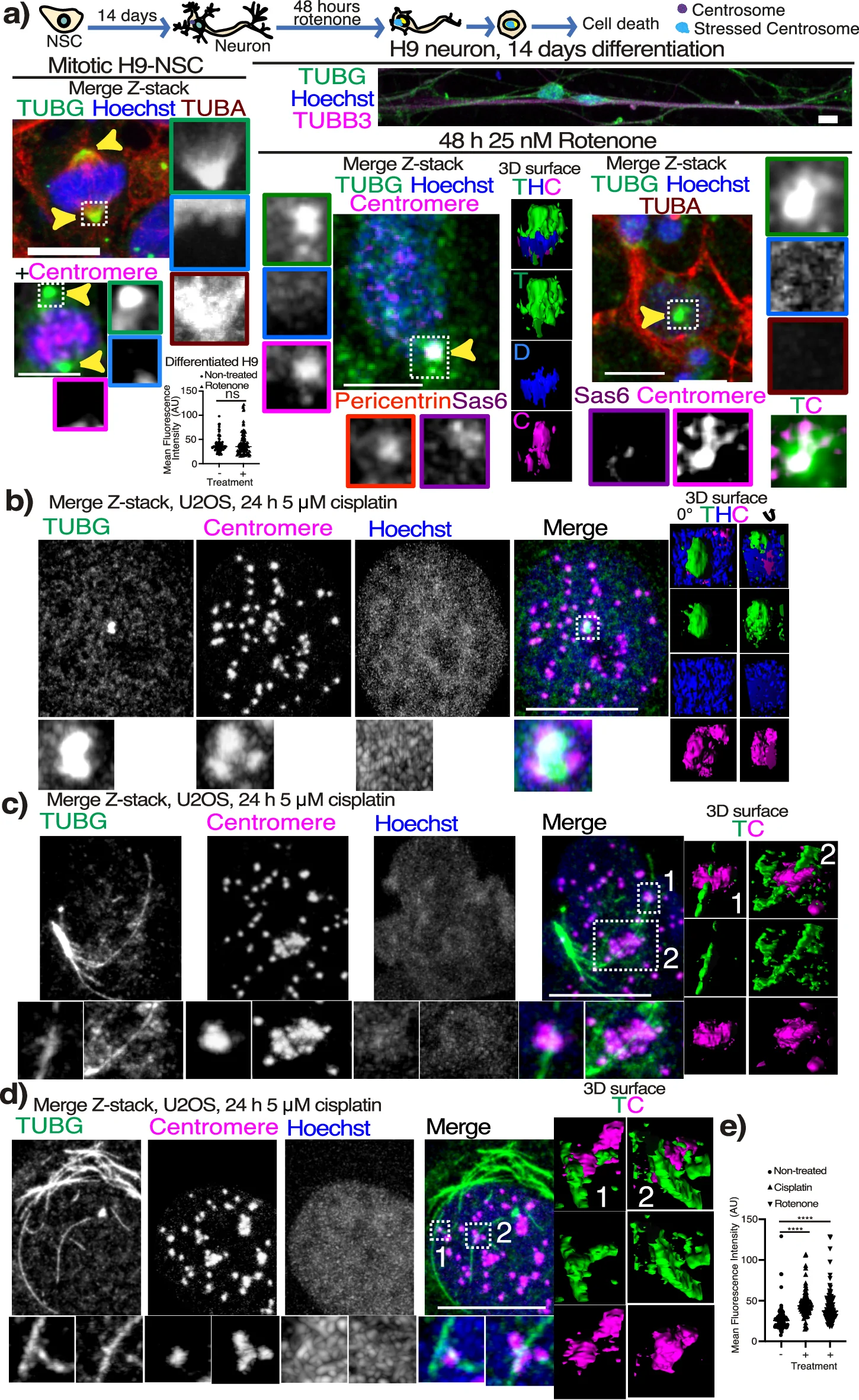
The TUBG meshwork is associated with centromere positioning under stress conditions. **a** Experimental timeline and maximum intensity projections (MIPs; Z-stack images, 0.4-μm intervals). Images show mitotic H9 neural stem cells (NSCs; left), NSCs differentiated into neurons over 14 days (top right), and neurons treated with rotenone (right) to induce oxidative stress. Cells were immunolabeled for centrosome markers (anti-TUBG, anti-Sas6, and anti-pericentrin), neuronal morphology (anti-β3-tubulin, TUBB3), centromere-associated proteins (anti-centromere antibody), and microtubules (anti-α-tubulin, TUBA). DNA was counterstained with Hoechst. Insets show 3D surface reconstructions (ImageJ 3D Viewer) of centrosomes (T), DNA (H), and centromeres displaying close spatial associations (C), rotated for optimal visualization. Scale bars: 10 μm in (**a**–**d**). Yellow arrowheads indicate centrosomes with mitotic-like characteristics. The accompanying graph depicts 3D quantification of TUBG mean fluorescence intensity within centromere volumes (defined by 3D segmentation, *n* = 100 per sample). Rotenone treatment was associated with a non-significant trend (*P* = 0.5959) toward higher TUBG fluorescence intensity within these volumes. Data represent mean ± SD from four independent experiments; statistical significance was determined using a two-sided Mann–Whitney *U* test. (**b**–**d**) Spatial associations between TUBG-containing structures and centromeres in U2OS cells following 24 h of cisplatin treatment. MIPs (Z-stack images, 0.1-μm intervals) show cells immunolabeled with anti-TUBG and anti-centromere antibodies, with DNA counterstained with Hoechst. Cropped images show 3D-surface reconstructions of centromeres (C) located near centrosomal TUBG hubs (**b**; T), near a ring-shaped γ-tubule (**c**; T), or at the intersection of two γ-tubules (**d**; T). **e** Summary of the 3D quantification of mean fluorescence intensity within centromere-defined volumes (*n* = 100 centromeres per sample) for the indicated U2OS experimental groups (**b**–**d**). Both cisplatin and rotenone treatments were associated with significantly higher TUBG fluorescence intensity within centromere-defined volumes compared to untreated controls. Data represent mean ± SD from three independent experiments. Statistical significance was determined using a two-sided Mann–Whitney *U* test (****P < 0.0001).

To investigate these stress-associated spatial relationships, we differentiated H9-NSC cells into neurons over 14 days and then exposed them to 25 nM rotenone for 48 hours to induce oxidative stress^30,31^. After rotenone treatment, neurons lost their elongated morphology and adopted a rounded, mitotic-like shape^30^. During this transition, centrosomes were frequently observed near chromatin regions containing centromeres (Fig. 5a). While these centrosomes resembled those in mitotic cells, they did not participate in spindle assembly; instead, centromeres were observed in close proximity to these sites. These observations indicate a stress-associated spatial relationship between non-mitotic centrosomal regions and centromere-containing chromatin but do not establish that centrosomes actively reposition centromeres.

3D quantification of these regions in H9-neurons showed a non-significant trend toward higher TUBG fluorescence intensity within centromere-defined volumes (Fig. 5a). Because this difference was not statistically significant, we interpret this dataset as showing a qualitative spatial rearrangement of the pre-existing TUBG pool rather than evidence of increased TUBG recruitment or altered centromere function.

The stress-associated nature of this spatial association was also examined in U2OS cells treated with 5 µM cisplatin, a DNA-damaging agent that induces genomic stress^27,32^. Similar to findings in the rotenone-treated H9-neurons (Fig. 5a), centromeres were observed near centrosomes in U2OS cells (Fig. 5b). Centromeres were also observed near ring-like γ-tubule structures (Fig. 5c) and γ-tubule intersections (Fig. 5d), demonstrating spatial associations between centromeric foci and distinct TUBG meshwork configurations under stress. Summary 3D quantification in U2OS cells showed that cisplatin and rotenone treatments were associated with significantly higher TUBG fluorescence intensity within centromere-defined volumes compared with untreated controls (*P* < 0.0001, Mann–Whitney test; Fig. 5e).

Unlike the H9-neuron dataset, the U2OS analyses showed statistically significant changes in TUBG fluorescence intensity within centromere-defined volumes. Differences between these models may reflect variation in baseline TUBG distribution, cell type, or stress response, but the present measurements do not distinguish among these possibilities. Together, these findings support a stress-associated spatial relationship between TUBG-containing structures and centromere-defined regions. They do not establish a direct interaction, altered centromere function, or causal control of centromere positioning by the TUBG meshwork.

### Ser131 substitutions are associated with TUBG meshwork assembly and centromere-associated dynamics

Our live-cell imaging revealed dynamic spatial associations between the TUBG meshwork, including centrosome-associated pools and γ-tubules, and interphase centromeres. Because TUBG–TUBG interactions form the structural basis of this meshwork, we next investigated whether the phosphorylation state of serine 131 (Ser131), modeled using non-phosphorylatable and phospho-mimetic substitutions, was associated with meshwork assembly and centromere-associated behavior. Previous studies have shown that the phospho-mimetic Ser131Asp substitution favors lateral TUBG self-assembly^33–35^. We therefore examined whether the two Ser131 mutants differed in TUBG-focus formation and spatial association with centromeres.

**Fig. 6:**
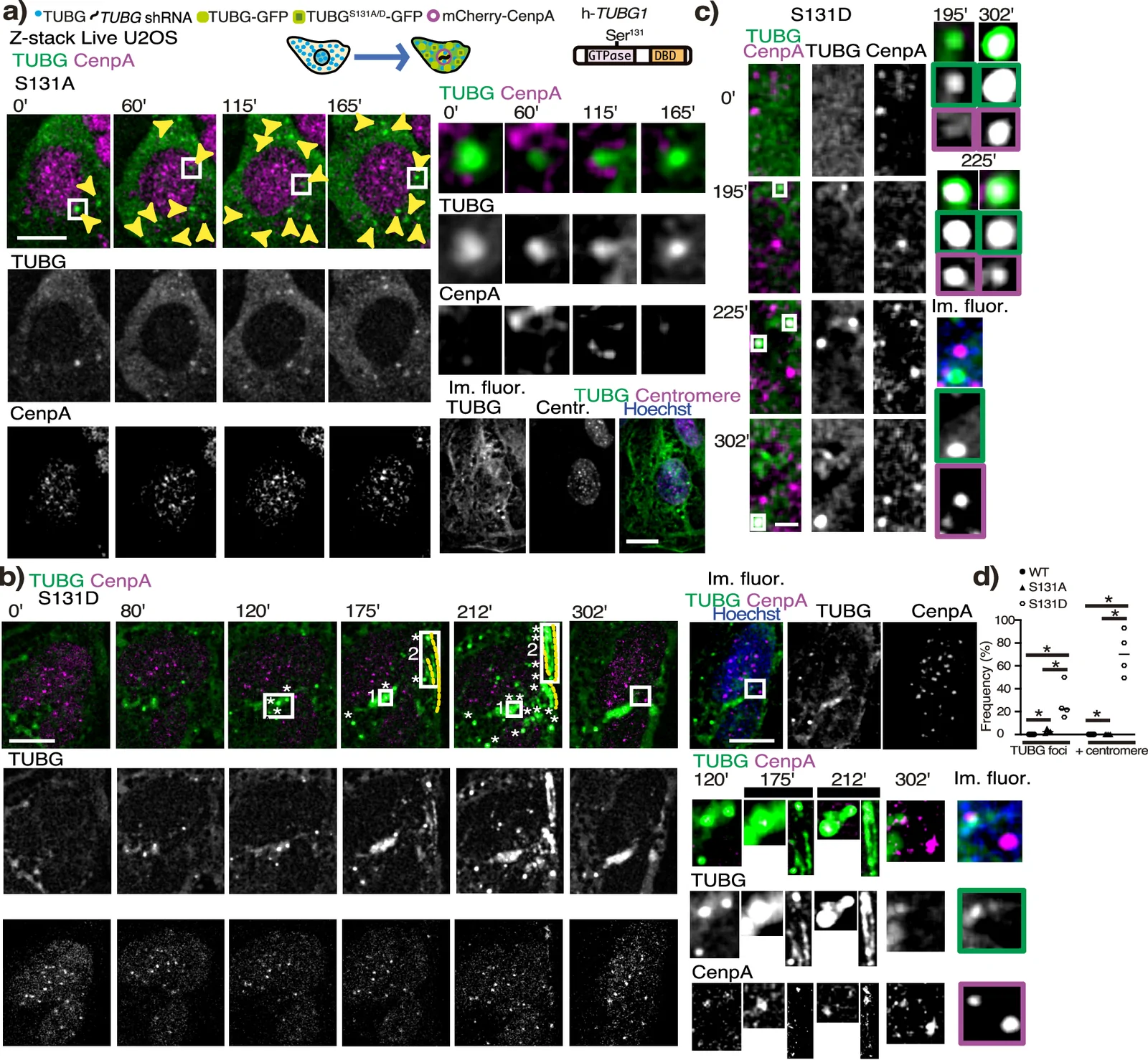
Ser131 mutant-associated TUBG–centromere dynamics and γ-tubule formation. **a**–**c** Live-cell imaging of non-phosphorylatable TUBG-GFP Ser131Ala and phospho-mimetic TUBG-GFP Ser131Asp mutants and their spatial associations with centromeres and γ-tubule formation. Sh*TUBG*-TUBG-CENP-A-U2OS cells (stably expressing Sh*TUBG* to reduce endogenous TUBG and mCherry-CENP-A as a centromere marker) were transiently transfected with either TUBG-GFP Ser131Ala (**a**) or TUBG-GFP Ser131Asp (**b**, **c**). Cells were imaged using a confocal microscope. Time-lapse Z-stack images were acquired at 2.5-minute intervals (with 1-μm intervals between Z-planes) and displayed as maximum intensity projections. Cells were treated with 5 μM cisplatin, and live-cell imaging was performed during treatment. Approximately 5 minutes after the last time-lapse frame, cells were fixed. Fixed cells were then immunostained with anti-TUBG (green boxes) and anti-centromere (magenta boxes) antibodies, with DNA counterstained using Hoechst. The total incubation time with cisplatin before fixation was 302.5 minutes. A schematic representation of the modifications made to the cell lines is included for context. White boxes indicate the magnified regions shown in the insets (images are representative of four independent experiments). Related to Supplementary Videos 4–6. The panels combine live-cell imaging with subsequent immunofluorescence staining (Im. fluor.) of fixed cells to confirm protein localization. Scale bars: 10 μm in (**a**, **b**); 2 μm in (**c**). **a** Time-lapse series showing the transient formation of TUBG-GFP Ser131Ala foci. For this specific series, fixation occurred 143 minutes after the last time-lapse frame shown. Yellow arrowheads indicate TUBG-GFP Ser131Ala foci that were not observed in close spatial association with centromeres (*n* = 148 cells). **b**, **c** Time-lapse sequences illustrating the dynamic formation of phospho-mimetic TUBG-GFP Ser131Asp foci (indicated by white asterisks; green) and their subsequent spatial association with the centromere marker mCherry-tagged CENP-A (magenta) (*n* = 126 cells). Immunofluorescence staining confirmed the incorporation of mCherry-CENP-A into endogenous centromeres. **b** This panel highlights the dynamic formation of TUBG-GFP Ser131Asp-labeled γ-tubules, denoted by yellow hatched lines. **c** This panel shows the dynamic formation of two TUBG-GFP Ser131Asp-labeled foci, with which centromeres became spatially associated and subsequently displayed coordinated movement. **d** Quantitative analysis of TUBG-focus formation and centromere association. The graph shows the percentage of total cells that formed TUBG foci and the percentage of TUBG-foci-positive cells in which the foci were spatially associated with centromeres. Individual data points represent the values obtained in each independent experiment for WT (filled circles), Ser131Ala/S131A (triangles), and Ser131Asp/S131D (open circles). The two *x* axis categories show the percentage of cells that formed TUBG foci and the percentage of cells in which TUBG foci were spatially associated with centromeres, respectively. Horizontal lines and error bars indicate the mean ± SD from four independent experiments. Total numbers of analyzed cells were WT, *n* = 254; Ser131Ala, *n* = 255; and Ser131Asp, *n* = 169. Statistical significance was determined using two-sided unpaired Mann–Whitney *U* tests. For TUBG-focus formation: WT versus Ser131Ala, *P* = 0.0286; WT versus Ser131Asp, *P* = 0.0286; Ser131Ala versus Ser131Asp, *P* = 0.0286. For spatial association of TUBG foci with centromeres: WT versus Ser131Asp, *P* = 0.0286; Ser131Ala versus Ser131Asp, *P* = 0.0286. (*P < 0.05).

To test this, we generated sh*TUBG*-TUBG-CENP-A-U2OS cells that transiently expressed either a non-phosphorylatable TUBG-GFP Ser131Ala mutant (Fig. 6a and Supplementary Video 4) or a phospho-mimetic TUBG-GFP Ser131Asp mutant (Fig. 6b, c and Supplementary Videos 5 and 6)^33^. Quantitative analysis (Fig. 6d) revealed that, compared with control TUBG-GFP-expressing cells (*n* = 254 cells), a significantly lower percentage of cells expressing the non-phosphorylatable mutant (2.7%; *n* = 255 cells) formed transient TUBG foci, whereas a higher percentage of cells expressing the phospho-mimetic mutant (26%; *n* = 169 cells) exhibited these structures (Fig. 6a–d).

Because γ-tubules emerge from these TUBG centers, making it difficult to resolve individual filaments from dense foci, we used TUBG-focus formation as the primary metric for TUBG meshwork assembly under these experimental conditions. Ser131Ala foci were rarely observed and were not detected in spatial association with centromeres in the analyzed live-cell sequences, whereas Ser131Asp foci formed more frequently and were observed in close spatial association with centromeres (Fig. 6d). The Ser131Asp mutant also formed γ-tubules near centromeric foci (Fig. 6b). Furthermore, live-cell imaging captured the dynamic movement of Ser131Asp foci, during which associated centromeres remained spatially apposed and displayed coordinated movement (Fig. 6c).

These differences between the Ser131Ala and Ser131Asp mutants indicate that the Ser131 substitution state is associated with TUBG-focus formation, γ-tubule assembly, and spatial association with centromeres under the conditions analyzed. Because phospho-mimetic and non-phosphorylatable mutants do not directly measure endogenous phosphorylation dynamics, these observations do not establish that Ser131 phosphorylation alone causes centromere recruitment or organization. Altogether, these findings support an association between the Ser131 substitution-dependent TUBG assembly state and centromere-associated spatial dynamics under stress.

### TUBG meshwork remodeling and centromere-associated localization under cellular stress

**Fig. 7:**
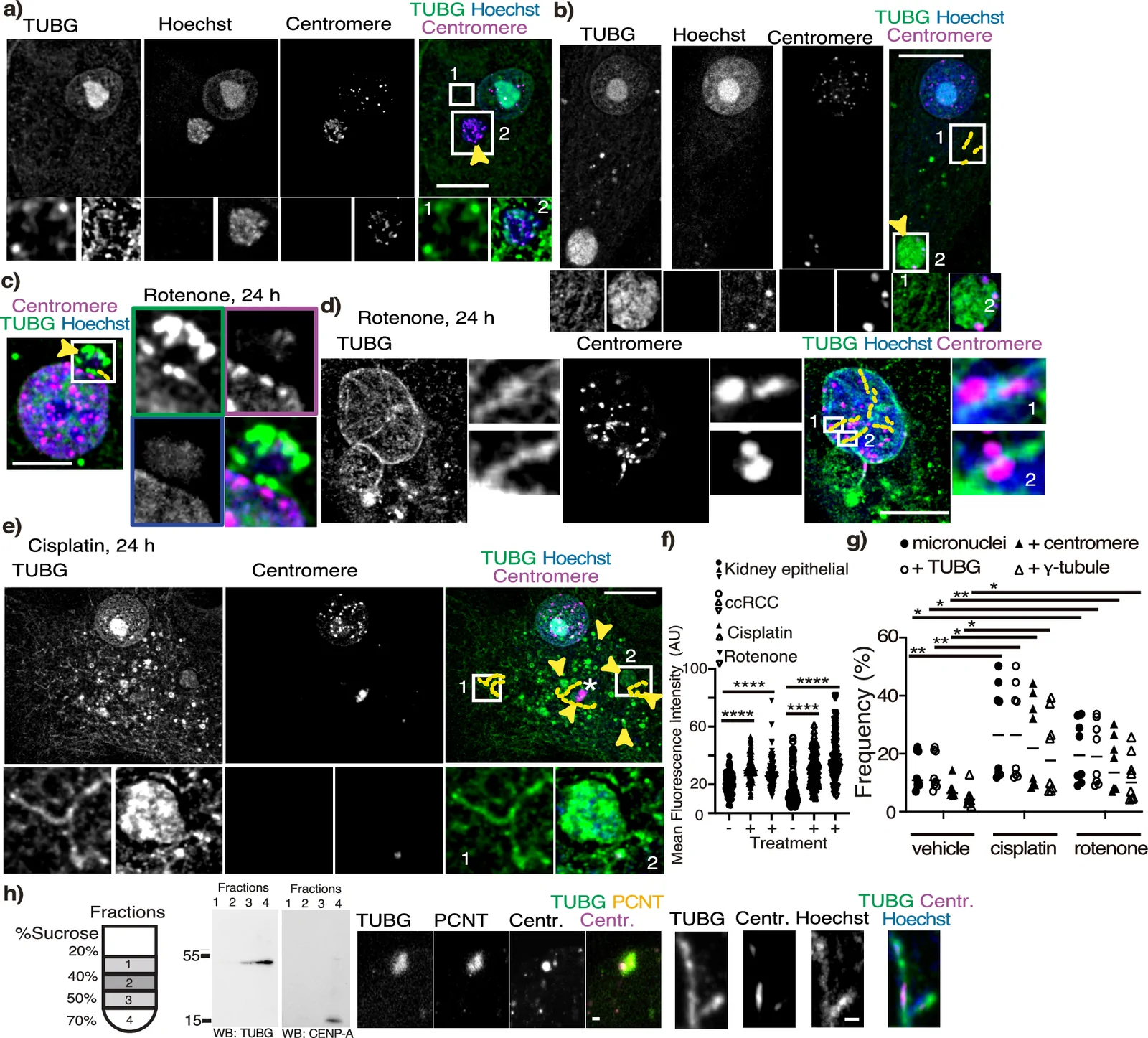
TUBG meshwork remodeling and centromere association under stress. **a**–**e** Immunofluorescence analysis of the TUBG meshwork and centromere-associated structures in primary renal cell carcinoma (ccRCC) cells. Cells were treated for 24 hours with DMSO (vehicle; **a**, **b**), 500 nM rotenone (**c**, **d**), or 5 μM cisplatin (**e**) prior to fixation. Cells were immunolabeled for TUBG and centromeres, and DNA was counterstained with Hoechst. Representative Z-stack maximum intensity projections (MIPs; 0.4-μm intervals) were acquired by confocal microscopy. White boxes denote magnified regions shown in the insets. Cropped image stacks highlight nuclear TUBG foci, γ-tubules (yellow hatched lines), and TUBG-positive micronuclei (yellow arrowheads). Scale bars in (**a**–**e**): 10 μm. Images are representative of four independent experiments. **a** DMSO-treated control cells showing two cytosolic TUBG-foci (inset 1) and a TUBG/centromere-positive micronucleus (inset 2). **b** DMSO-treated control cells illustrating γ-tubules (inset 1) and a TUBG/centromere-positive micronucleus (inset 2). **c** Rotenone-treated cell showing a centromere-positive nucleus with multiple TUBG-foci, from which a γ-tubule extends. **d** Rotenone-treated cell demonstrating nuclear γ-tubules in close association with centromeres. **e** Cisplatin-treated cell showing cytosolic γ-tubules (inset 1) in close spatial association with a TUBG-positive micronucleus (inset 2). **f** Summary of the 3D quantification of mean TUBG fluorescence intensity within centromere volumes (*n* = 100 individual centromeres per sample) in primary human normal kidney epithelial cells and ccRCC cells. Both cisplatin and rotenone treatments were associated with significantly higher TUBG fluorescence intensity within these volumes compared to untreated controls in both cell types. Data represent mean ± SD from three independent experiments. Statistical significance was determined using a two-sided Mann–Whitney *U* test (****P < 0.0001). Symbols correspond to the treatment groups indicated in the graph key: untreated/control, cisplatin-treated, and rotenone-treated samples. Statistical comparisons were performed between each treated group and its corresponding untreated control unless otherwise indicated. **g** Micronuclei were identified as discrete Hoechst-positive cytosolic chromatin bodies separated from the main nucleus and were subsequently scored for TUBG signal, centromere signal, or spatial association with γ-tubules. The graph shows the frequency of micronuclei and the frequencies of micronuclei associated with different components of the TUBG meshwork: TUBG-positive micronuclei, centromere-positive micronuclei, and micronuclei spatially associated with γ-tubules, in vehicle-treated (*n* = 1615 cells), cisplatin-treated (*n* = 1633 cells), and rotenone-treated (*n* = 1623 cells) populations. Filled circles indicate micronuclei, open circles indicate TUBG-positive micronuclei, filled triangles indicate centromere-positive micronuclei, and open triangles indicate micronuclei spatially associated with γ-tubules. Each data point represents one independent experiment, and horizontal lines indicate the mean from four independent experiments. Statistical significance was determined using two-sided paired *t* tests. For total micronuclei: vehicle vs cisplatin treatment, *P* = 0.0089; vehicle vs rotenone treatment, *P* = 0.0108. For TUBG-positive micronuclei: vehicle vs cisplatin treatment, *P* = 0.0098; vehicle vs rotenone treatment, *P* = 0.0145. For centromere-positive micronuclei: vehicle vs cisplatin treatment, *P* = 0.0141; vehicle vs rotenone treatment, *P* = 0.0184. For micronuclei spatially associated with γ-tubules: vehicle vs cisplatin treatment, *P* = 0.0181; vehicle vs rotenone treatment, *P* = 0.0229. (*P < 0.05, **P < 0.01). **h** Biochemical analysis of isolated γ-tubulin complexes. U2OS cells were treated with cisplatin (24 h) followed by pretreatment with cytochalasin B and the γ-tubule-stabilizer, L12. TUBG-rich fractions were isolated from nuclear extracts by a discontinuous sucrose gradient (schematic). Fractions were analyzed by western blotting (WB) for TUBG and CENP-A. Representative single confocal images display isolated centrosomes labeled with anti-TUBG, anti-pericentrin (PCNT), and anti-centromere antibodies. Maximum intensity projections (0.1-μm intervals) show isolated γ-tubules after immunostaining with anti-TUBG and anti-centromere (centr.) antibodies; DNA was counterstained with Hoechst. The co-fractionation and spatial proximity of these components support a stable association under the extraction conditions used but do not establish direct molecular interaction (*n* = 4). Scale bars in **h**: 1 μm.

Our preceding analyses showed spatial associations between TUBG-containing structures and interphase centromeres and indicated that Ser131 substitutions are associated with TUBG meshwork assembly and centromere-related dynamics^33–35^. Given the known role of the TUBG meshwork in organizing mitochondria in ccRCC cells^22^, we investigated how this network responds to mitochondrial stress (rotenone) and DNA damage (cisplatin) in primary ccRCC cells (Fig. 7a–g).

In control cells, we observed baseline cytosolic TUBG foci and γ-tubules^4,22,23^. Following rotenone treatment, we observed remodeling of the TUBG meshwork, including more frequent observation of centromere-positive nuclei containing multiple TUBG foci from which γ-tubules extended (Fig. 7c, d). Similarly, cisplatin treatment was associated with a higher frequency of cytosolic γ-tubules, which were often located near TUBG-positive micronuclei (Fig. 7e). The presence of micronuclei under stress is consistent with genomic instability induced by genotoxic or oxidative stress, which may involve mitotic defects, chromosome mis-segregation, mitotic catastrophe, or other mechanisms that were not directly investigated in the present study^36^.

3D volumetric analysis showed that both stressors were associated with significantly higher TUBG fluorescence intensity within centromere-defined volumes (*P* < 0.0001, Fig. 7f). While NSC neurons (Fig. 5) exhibited a non-significant trend, the significant results in ccRCC and U2OS cells demonstrate reproducible stress-associated increases in TUBG fluorescence within centromere-defined volumes in these two models. Differences between the H9-neuron, ccRCC, and U2OS datasets may reflect cell-type-specific baseline TUBG distribution or stress responses; however, the present measurements do not distinguish among these possibilities.

Furthermore, stress was associated with a significant change in the distribution of TUBG-associated structures around micronuclei, including an increased proportion of micronuclei spatially associated with γ-tubules (*P < 0.05, Fig. 7g). This finding indicates stress-associated remodeling of the TUBG meshwork near micronuclei but does not by itself demonstrate active recruitment or altered micronuclear function. This structural reorganization may occur in coordination with other cellular responses, such as chromatin remodeling and DNA damage signaling. The observed spatial association is consistent with a possible relationship between the TUBG meshwork and the compartmentalization of displaced genomic material, although a direct structural or functional role has not been established.

To further assess this biochemical association, we biochemically isolated TUBG complexes from nuclear extracts. Western blot and subsequent immunofluorescence of these isolated fractions showed that centrosomes and γ-tubules co-fractionate with centromere-containing material (Fig. 7h). Retention of this association during biochemical extraction supports stable co-fractionation under the conditions used but does not distinguish direct interaction from association through larger protein- or chromatin-containing assemblies. This association is reminiscent of the centrosomal accumulation of proteins observed during proteolytic aggresome formation^30^, but the present data do not establish that the TUBG meshwork performs an equivalent sequestration mechanism in response to genomic or metabolic stress.

### TUBG foci are associated with assembly sites within the meshwork

**Fig. 8:**
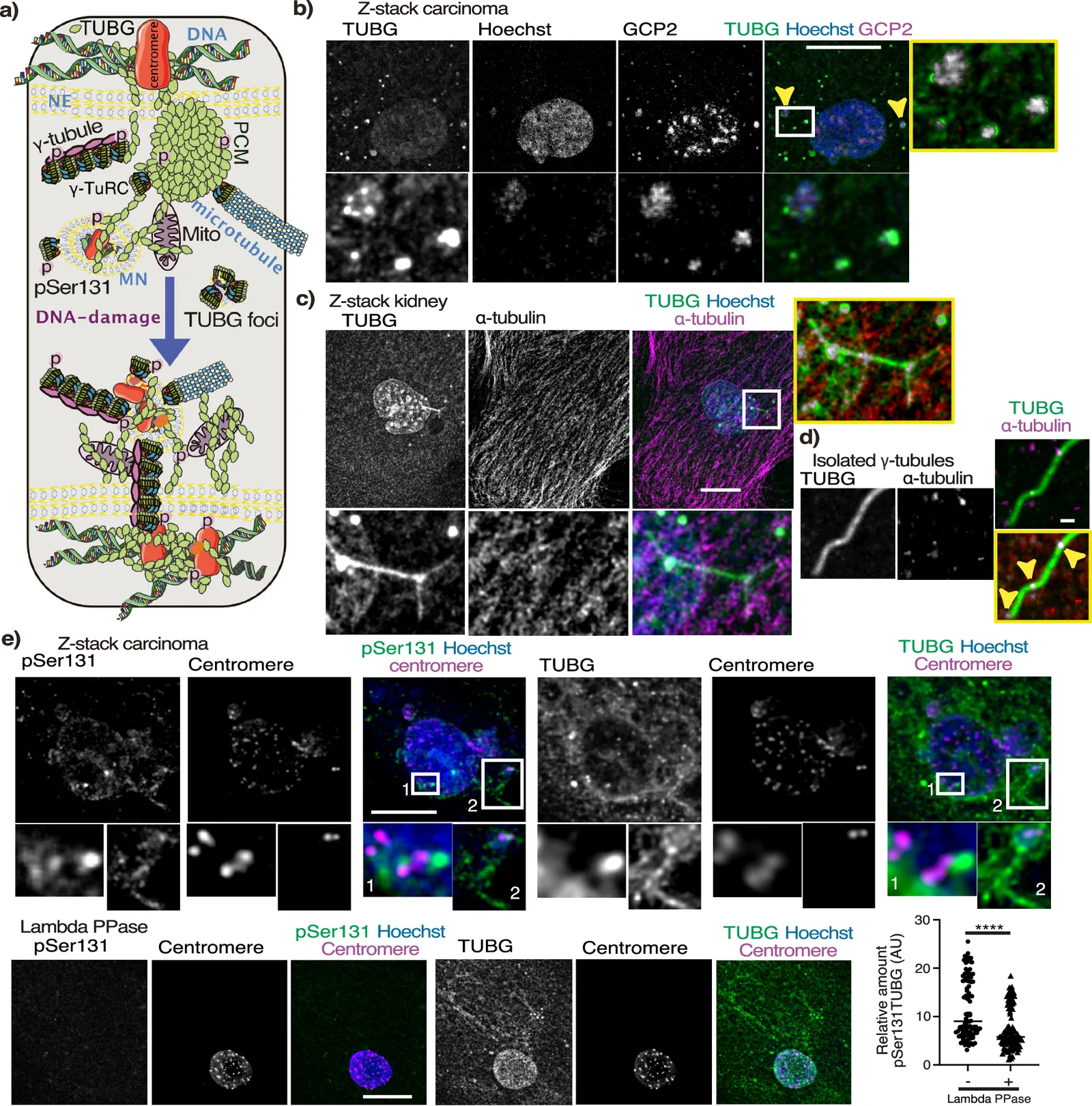
Subcellular localization and spatial associations of TUBG foci. **a** Schematic representation of TUBG meshwork organization and remodeling during cellular stress. TUBG foci are depicted as assembly-associated sites within the meshwork; under stress, TUBG is observed in association with micronuclei and γ-tubules as part of meshwork remodeling (NE nuclear envelope, PCM pericentriolar matrix, γ-TuRC TUBG-ring complex, Mito mitochondria, MN micronucleus, pSer131, Ser131-phosphorylated TUBG). **a** was assembled by the authors in Affinity Designer. Selected elements in (**a**) were adapted, with modifications, from Corvaisier and Alvarado-Kristensson, *Cancers* 12, 3102 (2020), under CC BY 4.0 licence^67^. **b**, **c**, **e** Immunofluorescence characterization of the TUBG meshwork components and Ser131 phosphorylation in human kidney cells. Representative Z-stack maximum intensity projections of fixed primary human renal carcinoma (**b**, **e**) and normal kidney epithelial (**c**) cells. Images were acquired with a confocal microscope at 0.4-μm intervals. White boxes denote magnified regions shown in the insets. Scale bars: 10 μm. Images are representative of four independent experiments. **b**, **c** Assessment of TUBG foci composition and spatial association with other cytoskeletal elements. Cells were immunofluorescently co-stained for TUBG and DNA (Hoechst), along with either anti-GCP2 (a γ-TuRC marker; **b**) or anti-α-tubulin (microtubule marker; **c**). **b** Cropped image stacks highlighting nuclear TUBG foci in close spatial association with GCP2. Yellow arrowheads indicate micronuclei with TUBG-associated foci. **c** Cropped image stacks highlighting TUBG foci and associated γ-tubules positioned near the microtubule network. **d** Analysis of isolated γ-tubules from cisplatin-treated U2OS cells. Representative single confocal images display an isolated γ-tubule after immunostaining with anti-TUBG and anti-α-tubulin antibodies. The colocalization pixel map (CPM) highlights discrete regions of spatial overlap/proximity between γ-tubules and α-tubulin (yellow arrowheads). Scale bars: 1 μm. **e** Characterization of TUBG phosphorylation in ccRCC cells. To confirm antibody specificity, samples were treated with lambda phosphatase (Lambda PPase) prior to immunostaining. The graph displays the fluorescence intensity of Ser131-phosphorylated TUBG measured using ImageJ. Statistical significance was determined using a two-sided Mann–Whitney *U* test (****P < 0.0001; *n* = 100 cells per condition pooled across *n* = 4 biologically independent experiments). **b**–**d** The yellow insets display CPMs, where white areas indicate colocalized pixels across channels.

We hypothesized that Ser131 phosphorylation may be associated with lateral self-assembly of TUBG, creating distinct TUBG foci within the broader meshwork (Fig. 8a). Immunofluorescence analysis showed that these foci were spatially associated with the γ-TuRC component GCP2 (Fig. 8b), consistent with the presence of a nucleation-associated component at these sites. Furthermore, these foci were observed near TUBG-containing structures extending between the nuclear compartment and the microtubule network (Fig. 8c)^23^.

To further examine these inter-cytoskeletal spatial associations at high resolution, we analyzed isolated γ-tubules from cisplatin-treated U2OS cells (Fig. 8d). Immunostaining for TUBG and α-tubulin, combined with colocalization pixel mapping (CPM), identified distinct regions of spatial overlap/proximity between γ-tubules and the microtubule lattice (Fig. 8d, yellow arrowheads). These observations support spatial associations between TUBG-containing γ-tubules and cytoplasmic microtubules but do not establish direct molecular interaction or physical continuity between the two structures.

To investigate Ser131 phosphorylation in relation to this meshwork organization, we utilized a phospho-specific Ser131 antibody. Analysis of ccRCC cells identified a punctate staining pattern on γ-tubules and TUBG foci (Fig. 8e) resembling the localization pattern observed with the phospho-mimetic TUBG-GFP Ser131Asp mutant (Fig. 6). The specificity of this modification was confirmed by lambda phosphatase treatment, which resulted in a near-complete loss of the phospho-Ser131 signal (Fig. 8e). However, the localization of the phospho-Ser131 signal and the behavior of a phospho-mimetic mutant do not by themselves establish that endogenous Ser131 phosphorylation directly controls meshwork assembly.

Together, these data indicate that γ-TuRC-associated TUBG foci are spatially associated with assembly-related components of the meshwork and that Ser131-phosphorylated TUBG localizes to these structures. These findings support an association between Ser131 phosphorylation and TUBG meshwork organization, without establishing direct causal regulation of assembly or function.

### TUBG foci dynamics in micronuclei and their spatial association with centromere-containing compartments

Our preceding analyses indicated spatial associations between the TUBG meshwork and centromere-associated structures and showed that Ser131 substitutions were associated with meshwork assembly and centromere-related dynamics under cellular stress (Fig. 8). Given that cellular stress and chromosome mis-segregation can lead to the formation of micronuclei^37^, which often encapsulate entire centromeric regions, we investigated whether TUBG foci are associated with the spatial organization of these compartments.

**Fig. 9:**
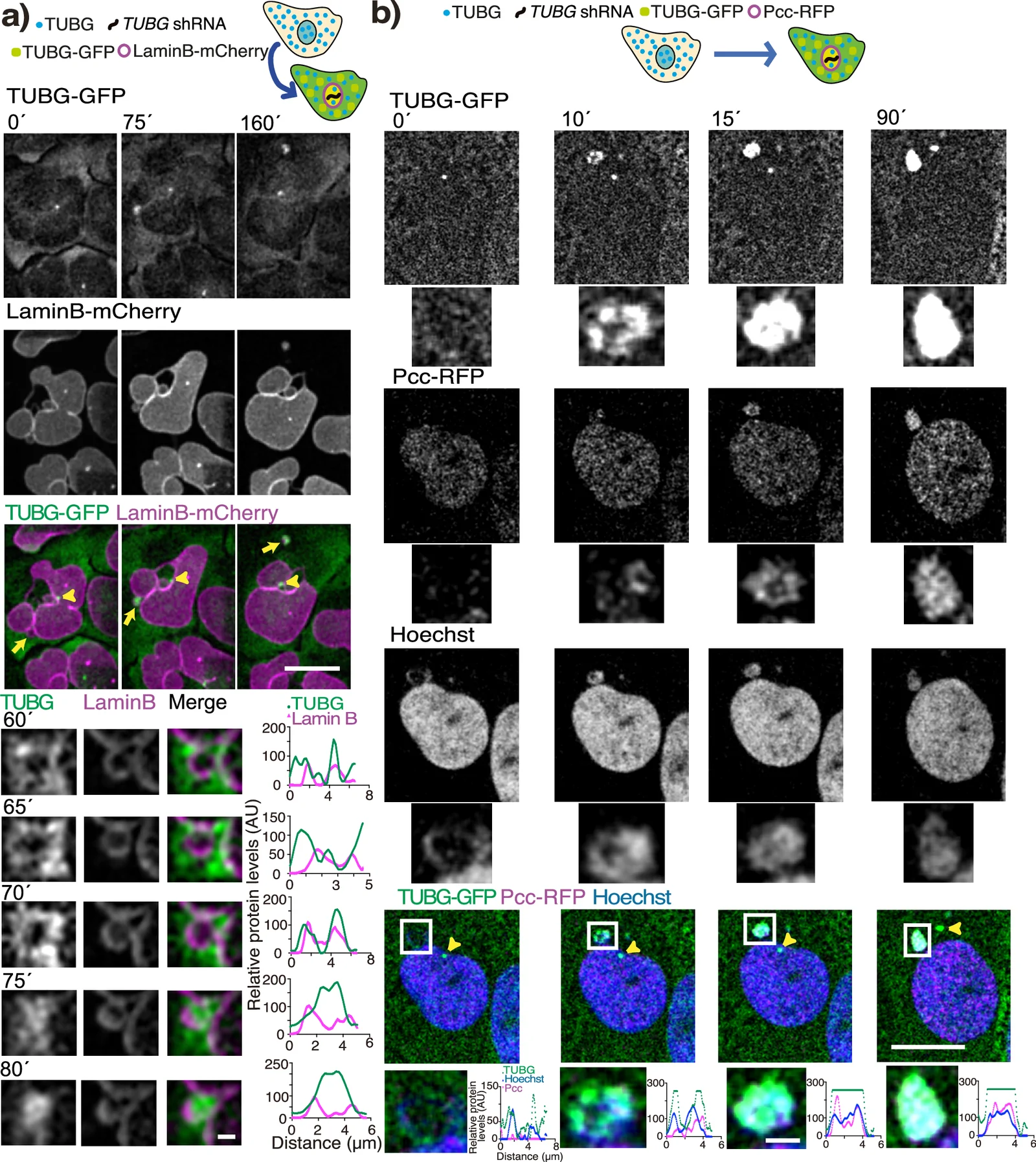
TUBG-foci dynamics in micronuclei. **a**, **b** Representative fluorescence time-lapse sequences showing TUBG-foci dynamics in micronuclei. U2OS cells stably expressing *TUBG1*-shRNA to reduce endogenous TUBG and coexpressing shRNA-resistant TUBG-GFP and either Lamin B-mCherry (a nuclear envelope marker; **a**) or PCNA-Chromobody-RFP (**b**) were imaged. Time-lapse Z-stack images were acquired at 5-minute intervals and displayed as maximum intensity projections. A schematic representation of the cell line modifications is included. Yellow arrowheads denote the centrosome (*n* = 200 cells, from four independent experiments). Scale bars: 10 μm for the full-field images in (**a**, **b**); 2 μm for the magnified insets. Related to Supplementary Videos 7 and 8. **a** Z-stack time-lapse frames (0.1-μm intervals), acquired using Airyscan high-resolution microscopy, depicting a micronucleus containing a TUBG-focus (TUBG-GFP; 60 min) positioned at its nuclear envelope (Lamin B-mCherry). This is followed by TUBG enrichment at the nuclear envelope (65–70 min) and subsequent accumulation of TUBG inside the micronucleus (75–80 min). Insets show the magnified micronucleus, illustrating this spatial progression. Yellow arrows denote the micronucleus. Dual-channel intensity profiles quantify the spatial relationship between TUBG-GFP and Lamin B-mCherry, consistent with movement of the TUBG signal from the envelope-associated region toward the micronuclear interior. **b** DNA was stained with Hoechst. Insets highlight the Z-stacks (with 1-μm intervals between Z-planes) within the white box, illustrating micronuclear accumulation of TUBG concurrent with PCNA-Chromobody (Pcc-RFP) signal and increased DNA-staining intensity. A multichannel intensity plot visualizes the temporal accumulation of TUBG-GFP, PCNA-Chromobody (Pcc-RFP), and Hoechst signal.

Using stable *TUBG*sh-U2OS cells co-expressing shRNA-resistant TUBG-GFP and Lamin B-mCherry (as a nuclear envelope marker), we observed the dynamic behavior of TUBG foci at the micronuclear envelope (Fig. 9a and Supplementary Video 7). Time-lapse imaging revealed TUBG foci initially localized at the micronuclear envelope (e.g., at 60 minutes), followed by progressive enrichment at the envelope (65–70 minutes), and subsequent accumulation within the micronucleus (75–80 minutes). Intensity profile analysis further supported this spatial progression, consistent with movement of the TUBG signal toward the micronuclear interior (Fig. 9a).

To examine temporal relationships between TUBG accumulation, a replication-associated marker, and the DNA signal, we monitored TUBG-GFP dynamics alongside PCNA-Chromobody-RFP (a marker for DNA replication) and DNA (Hoechst) within micronuclei (Fig. 9b and Supplementary Video 8). We observed that TUBG accumulation coincided temporally with increased PCNA-Chromobody and DNA signals. These observations demonstrate a temporal association but do not establish that TUBG directly regulates DNA replication, chromatin organization, or micronuclear function.

Together, the data indicate that TUBG foci dynamically accumulate at and within micronuclei and are temporally associated with replication-related and DNA-fluorescence changes in these compartments. This behavior supports an association between the TUBG meshwork and sequestered chromatin under stress conditions, without establishing a direct structural or functional role^37,38^.

## Discussion

This study extends the structural context of TUBG beyond microtubule nucleation by highlighting associations with centrosomes, centromeres, chromatin organization, nuclear architecture, cellular stress responses, and micronuclear organization. Consistent with reports of nuclear TUBG functions in chromatin organization and the recruitment of replication-associated machinery^4,6,11,39^, our data show reproducible associations between TUBG and centromere-defined structures.

ChIP-seq revealed reproducible TUBG-associated signal near annotated centromeres, while immunofluorescence detected TUBG fluorescence within centromere-defined volumes. These findings support a model in which TUBG is associated with the structural environment of centromeres and with their spatial relationship to centrosomes through nuclear TUBG-containing assemblies. Alongside centromere-associated proteins and complexes such as Cdk5rap2, CENP-A, and PBAF^40,41^, TUBG may represent an additional component associated with these regions.

TUBG-containing γ-strings and γ-tubules extended across the nuclear envelope and were spatially associated with centrosomes, centromeres, chromatin, Lamin B, and NUP50. Consistent with previous descriptions of filamentous TUBG networks^6,11,42,43^, these observations support an association between the TUBG meshwork and both chromatin organization and centromere positioning. However, this architectural model remains inferential; higher-resolution approaches, including cryo-electron tomography, are required to determine whether direct physical continuity exists among these structures. The imaging and biochemical data support recurrent spatial associations between TUBG-containing structures, centromere-defined regions, and the nuclear compartment, but do not establish a molecular interaction or causal mechanism under the conditions analyzed.

Phospho-Ser131 signal localized to TUBG-containing foci and was associated with γ-tubule assembly, centromere-related dynamics, and meshwork remodeling. The phospho-mimetic TUBG-GFP Ser131Asp mutant was likewise associated with TUBG focus formation, γ-tubule assembly, spatial association with centromeres, and coordinated centromere movement. These findings support an association between Ser131 phosphorylation and TUBG meshwork organization^33,35,44–46^, but phospho-mimetic and non-phosphorylatable mutants do not establish direct control by endogenous Ser131 phosphorylation.

Our 4D analyses indicate that TUBG depletion is associated with reduced centromere mobility and increased centromere fluorescence intensity. The data do not distinguish direct effects from indirect effects mediated through nuclear architecture, chromatin organization, cell cycle progression, or DNA damage signaling^47,48^. Fluorescence intensity alone does not demonstrate chromatin compaction, condensation, altered centromere function, or altered centromere volume; therefore, we conservatively interpret the phenotype as increased centromere fluorescence intensity associated with reduced mobility.

The association of TUBG with damaged or displaced genomic material parallels its role in aggresome-associated sequestration of protein aggregates^30^. Under genotoxic stress, the TUBG–centromere association may similarly relate to chromatin compartmentalization. Cisplatin and rotenone were associated with higher TUBG fluorescence intensity within centromere-defined volumes, supporting stress-associated meshwork remodeling. In H9 neurons, however, the increase was not statistically significant; therefore, this dataset is interpreted only as showing qualitative redistribution of the pre-existing meshwork rather than evidence of de novo recruitment.

Live-cell imaging showed TUBG accumulation from the micronuclear envelope toward the interior, coinciding with increased PCNA-Chromobody and DNA signals. This temporal association does not establish direct regulation of DNA replication, chromatin organization, or micronuclear function. Because mitotic figures were rare in primary kidney and ccRCC cultures, mitotic arrest, abnormal mitoses, and the origin of micronuclei could not be robustly assessed. Nevertheless, TUBG-containing structures were recurrently associated with micronuclei during interphase, consistent with a spatial relationship to displaced genomic material^37,38^.

Because elevated TUBG expression is frequently observed in tumors^20,49^, these findings provide a framework for understanding how TUBG-associated structures relate to chromatin organization and adaptation to genomic stress^50,51^. Overall, TUBG-containing structures are associated with centromere positioning, centromere mobility, and micronuclear organization, providing a framework for investigating their relationship to structural and genomic integrity under stress.

## Materials and methods

### Chemicals and reagents

The following antibodies and reagents were utilized in the study: anti-CENP-A (1:400) from Cell Signaling, anti-TUBG (1:900, rabbit, cat. no. T3320) from Sigma-Aldrich, anti-centromere protein (1:150, human, cat. no. SKU 15-234) from Antibodies Inc.; and anti-α-tubulin (1:1500; cat. no. CP06) from Calbiochem; anti-Nuclear Pore Complex (1:200; cat. no. MAb414) from BioLegend; anti-SAS6 (1:200; cat. no. sc-376836), β3-tubulin (1:500; cat. no. sc-80005), anti-pericentrin 1 (1:150; cat. no. sc-376111), anti-GCP2 (1:50; cat. no. sc-377117) and anti-Lamin B (1:1800; cat. no. sc-6216) all from Santa Cruz Biotechnology; anti-phospho-Ser131-TUBG (1:160) antibody was prepared and characterized as previously described^33^ and cell cycle chromobody encoding an anti-PCNA V_H_H (anti-PCNA nanobody) fused to red fluorescent protein (RFP; Pcc-RFP) (Chromotek, Germany).

### Cell culture

H9-NSCs were kindly provided by Dr. L. Karayan-Tapon (Poitiers University Hospital, France^52^) and were cultured and differentiated as previously described^4^. Neuronal cell death was induced by treating the cells with 25 nM rotenone (Chemtronica AB) for 48 hours^30^.

U2OS cells (cat. no. HTB-96 from ATCC) were cultured according to the manufacturer’s instructions. Cells were treated with 5 μM cisplatin for various times (Chemtronica AB) and with 500 nM rotenone for 24 hours before fixation.

U2OS cells were transfected with X-fect (Takara Bio Europa, cat. no. 631317) according to the manufacturer’s instructions. One day after transfection with *TUBG1* sgRNA, 40,000 cells were plated onto 35 mm glass-bottom dishes (MatTek, cat. no. p35G-1.0-20-C). The cells were then incubated for 7 days, with culture medium replaced every two days. To minimize cell death, cells expressing Cas9-GFP-*TUBG1*-sgRNA were not passaged, as they are particularly sensitive to excessive handling. Cells expressing *TUBG1* sgRNA were fixed after 7 days; this extended time point was optimized to characterize the phenotypic consequences associated with chronic, sublethal TUBG1 deficiency while minimizing terminal cell loss, as depletions exceeding 50% have been observed to be lethal in this model^22^. For all sgRNA depletion experiments, transfected (Cas9-GFP-positive) and non-transfected (Cas9-GFP-negative) cells were maintained in the same culture dishes to allow for internally controlled comparisons within identical imaging fields. When cells co-expressed either TUBG^1-335^ (Addgene plasmid 226519) or TUBG^334-451^ (Addgene plasmid 104436) sgRNA-resistant constructs (1:1), cells were fixed after 11 days^11^.

To ensure standardized depletion levels and a robust perturbation-associated readout within the viable cell population, we implemented a high-stringency single-cell validation approach. Only GFP-positive cells that exhibited a qualitatively discernible and quantitatively confirmed reduction in TUBG fluorescence intensity—averaging a 35.66% decrease relative to neighboring non-expressing (GFP-negative) internal control cells—were included in the final analysis. GFP-positive “escapee” cells that maintained baseline TUBG protein levels were strictly excluded. This single-cell protein-level verification supports the interpretation that the measured centromere-associated phenotypes are associated with reduced TUBG levels rather than with a generic response to the Cas9-GFP vector or secondary effects of active programmed cell death.

Primary human kidney epithelial and renal carcinoma cells were isolated from patient nephrectomy specimens after informed consent was obtained from all participants. The study was conducted in accordance with the Declaration of Helsinki and was approved by the Swedish Ethical Review Authority (LU680-08 and LU 289-07). Tumors were classified as ccRCC by an experienced pathologist. Normal kidney samples were collected from healthy cortex regions farthest from the tumor site. Excised tissue was sectioned and incubated overnight with a solution containing 300 U/ml Collagenase type I (Gibco) and with 200 U/ml DNase I (Sigma-Aldrich) in full medium. The next day, cells were collected, treated with trypsin for 5 min, and the cell suspension was serially filtered through 40-µm and 20-µm cell strainers. Isolated cells were cultured in high-glucose DMEM supplemented with 10% FBS and 1% penicillin/streptomycin (Thermo Scientific). Participant sex information was available in the clinical records but was not prespecified as an experimental variable and was not used to stratify the samples. Gender was not evaluated. Consequently, no sex- or gender-based analyses were performed because the study was not designed to assess sex- or gender-related differences.

To reduce endogenous TUBG levels, we stably transfected U2OS cells with pTER human *TUBG* short hairpin RNA (shRNA; Addgene plasmid 87955), which decreased endogenous TUBG expression by approximately 50%. To restore TUBG function, we co-expressed a shRNA-resistant *TUBG* gene tagged with green fluorescent protein (TUBG-GFP; Addgene plasmid 87956). U2OS cells co-expressing stably transfected *TUBG*-shRNA and shRNA-resistant TUBG-GFP were obtained by selection with 100 μg/ml zeocin (Invitrogen) and 200 μg/ml G418 (Sigma-Aldrich). A non-phosphorylatable TUBG-GFP Ser131Ala mutant or a phospho-mimetic TUBG-GFP Ser131Asp^33^ were transfected, and the resulting cells were imaged 12 hours after transfection. The inner nuclear membrane was labeled using either a *Lamin B1*-mCherry-tagged construct (pReceiver-M55; GeneCopoeia) or a mCherry-tagged nucleoporin 50 (*NUP50*-N10) provided by Michael Davidson (Addgene plasmid 55111). Centromeres were visualized with a mCherry-tagged CENP-A construct (*cenpA*-N-22), also from Michael Davidson (Addgene plasmid 62742). The spatial association of centrosome-associated TUBG with chromatin was analyzed using live imaging and fixed cells. The temporal relationship between TUBG accumulation and the replication-associated PCNA-Chromobody signal within micronuclei was monitored by coexpressing Pcc-RFP.

### Microscopy

U2OS cells were cultured and fixed following established protocols^4,53^. Fixation was performed in two steps: initial fixation with paraformaldehyde, followed by permeabilization and final fixation with a methanol-acetone mixture (1:1)^53^. DNA was stained for 1 h with 1 μg/ml Hoechst 33258 (Thermo Fisher Scientific).

Confocal fluorescence imaging was conducted using the following microscopes: Zeiss LSM 700 Axio Observer, Nikon A1RHD, a Zeiss LSM 880 with Airyscan, and Zeiss LSM 900 with Airyscan 2. All systems were equipped with Plan-Apochromat ×40 or ×63 NA 1.40 oil immersion objectives. Sequential images were captured at intervals of 0.1, 0.2, 0.4, or 1 μm, and time-lapse images were acquired at intervals of 1, 2, or 5 minutes.

### Image processing and visualization

Image processing was performed using ImageJ (Fiji, version 2.16.0/1.54p)^54^. Rolling-ball background subtraction was applied to images used for visualization. Unless otherwise stated, quantitative fluorescence-intensity measurements were performed using the corresponding raw image channels without background subtraction or normalization. Z-stack projections and further analyses were carried out using ImageJ. For 3D volumetric and live-cell imaging analyses, the canonical centrosome was identified and traced throughout Z-stacks based on its high-intensity spherical morphology, allowing for its spatial segregation from the elongated γ-tubule network. This structural differentiation was validated by SAS6 labeling in fixed material and further supported by pericentrin labeling in isolated biochemical complexes, ensuring that the quantified stress-induced association of TUBG with centromeres was measured independently of the main centrosomal body.

The 3D Viewer plugin (Fiji) was employed to generate 3D surface images^55^, while the Colocalization Threshold plugin was used to assess colocalization between fluorescent channels^54^. This plugin generates an image where areas of colocalization are displayed, with whiter regions indicating a greater degree of colocalization. All quantitative data and displacement tracking results were exported to GraphPad Prism (Version 10.05) for statistical analysis and visualization.

Z-stacks used for 3D centromere-volume analyses were processed using Fiji. To reduce high-frequency noise while preserving edge integrity, a 3D median filter (Radius: *X* = 2, *Y* = 2, *Z* = 1.5 pixels) was applied to the centromere channel. 3D segmentation was performed by applying a global Otsu threshold to the stack histogram to generate a binary mask, where centromeres were defined as foreground (value = 255) against a dark background (value = 0). The 3D Manager plugin (3D ImageJ Suite^26^) was employed to identify and isolate individual centromeric volumes. Objects were filtered by volume to exclude non-specific noise, and the resulting 3D regions of interest (ROIs) were saved as a spatial map.

Importantly, TUBG–centromere association was not assessed by visual inspection of merged maximum-intensity projections (MIPs). Instead, centromeres were defined objectively in three dimensions using the anti-centromere channel, and the resulting centromere-defined 3D ROIs were redirected to the corresponding raw TUBG channel throughout the complete Z-stack. For the analysis presented in Fig. 1e only, the same centromere-defined ROIs were additionally applied to the raw Lamin B channel to permit a paired comparison of TUBG and Lamin B fluorescence intensities. All segmentable centromeres within the analyzed cells were included when an individual centromere-defined 3D volume could be resolved and segmented. Thus, the primary unit of analysis was the individual centromere-defined volume rather than a binary cell-level score. In all other analyses, TUBG fluorescence intensity was quantified directly within centromere-defined volumes without comparison with Lamin B. TUBG fluorescence was analyzed as a continuous intensity variable, and no binary threshold was used to classify individual centromeres as TUBG-positive or TUBG-negative. Accordingly, the analysis reports continuous fluorescence-intensity measurements across the analyzed centromere-defined volumes and was not used to establish universal TUBG detectability across individual centromeres.

For all 3D ROI-based analyses, intensity values were extracted from raw data without background subtraction or normalization. Because representative images are displayed as partial MIPs and composites, fluorescence signals or spatial relationships confined to individual Z-planes may not be fully resolved when the Z-stacks are collapsed into two-dimensional projections. The displayed images were therefore used for illustration, whereas quantitative conclusions were derived from the complete Z-stacks using the segmented 3D ROIs. To compare protein distribution patterns between channels, line profile analysis was performed^56^. A linear ROI was drawn across regions of interest, and the Plot Profile tool was used to extract fluorescence intensity values along the distance of the line. Resulting data were exported to GraphPad Prism for visualization.

### 3D volumetric reconstruction and compartmentalization analysis

To quantify the spatial relationship between the centrosome, the nuclear envelope (NE), and chromatin, Z-stacks acquired via Airyscan 2 (0.1-μm intervals) were processed using Fiji (ImageJ). Raw channels for TUBG, NE markers, and Hoechst (DNA) were segmented into 3D binary masks using the 3D Simple Segmentation tool within the 3D ImageJ Suite^26^. To define a solid nuclear volume, a combined NE mask was generated based on the available markers: for fixed-cell datasets, a Boolean OR operation was performed on the NPC and LMNB channels, whereas for live-cell imaging, the LMNB channel was used exclusively. This initial mask was followed by a 3D Fill Holes operation to create a solid “Nuclear Interior” mask.

The total centrosomal volume was calculated from the binary TUBG mask. The encroaching centrosomal fraction (internal TUBG) was isolated by performing a Boolean AND operation between the TUBG mask and the solid nuclear interior mask. To identify the specific spatial overlap/proximity region of the cytosolic-nuclear bridge, a secondary AND operation was performed between the internal TUBG mask and the Hoechst (DNA) mask, yielding the TUBG-DNA spatial overlap/proximity volume.

All volumetric measurements (μm^3^) were extracted using the 3D Manager plugin. The Encroachment Percentage was defined as (Internal TUBG volume/Total TUBG Volume) × 100. The “DNA spatial overlap/proximity index” was calculated as the ratio of TUBG–DNA spatial overlap/proximity volume to Internal TUBG volume, providing a normalized measure of spatial association with chromatin. For visualization, 3D surface renderings were generated using the 3D Viewer plugin, with the NE rendered as a semi-transparent cyan surface (Alpha 0.3) to allow for the visualization of internal structures.

For fluorescence analyses not based on the segmented 3D centromere-defined volumes described above, fluorescence intensity measurements for centromeres and pSer131 TUBG were performed using MIPs of sequential images. Regions of interest were manually defined on staining patterns. Fluorescence intensity values reported in this manuscript represent raw data extracted directly from ImageJ. To ensure consistency, all measurements were conducted under identical conditions across samples.

For the categorical analysis presented in Fig. 7g, micronuclei were identified independently of the TUBG and centromere channels as discrete Hoechst-positive chromatin bodies located in the cytoplasm and spatially separated from the main nucleus. Each Hoechst-defined micronucleus was subsequently classified according to whether TUBG or centromere immunofluorescence was present within or spatially overlapping the micronuclear chromatin body and whether the micronucleus was spatially associated with a γ-tubule. This categorical micronucleus analysis was distinct from the continuous 3D fluorescence-intensity measurements performed within segmented centromere-defined volumes.

For immunofluorescence analysis of pSer131-TUBG, two matched dishes of ccRCC cells were prepared. Following the initial fixation step, one dish was treated with λ-phosphatase (New England Biolabs), according to the manufacturer’s instructions, while the other remained untreated. Subsequently, both phosphatase-treated and untreated samples were first stained with an anti-centromere antibody and an anti-pSer131-TUBG antibody, followed by their respective fluorophore-conjugated secondary antibodies. After this first round of staining and an additional fixation step, samples were then immunofluorescently labeled with an anti-TUBG antibody (cat. no. T3320).

To resolve overlap/proximity points between γ-tubules and the microtubule lattice, CPM was performed on isolated complexes. This method utilizes a Boolean intersection of thresholded pixels from the TUBG and α-tubulin channels to identify discrete overlap/proximity points.

### Live-cell centrosome tracking and dynamic analysis

To quantify the axial dynamics of the centrosome relative to the NE in living cells, Z-stack time-lapse sequences (acquired at 0.1-μm axial intervals) were analyzed. Centrosome position was tracked in the Z-dimension across sequential frames (*n* = 1050 frames) using the manual tracking plugin in Fiji. For each centrosome (*n* = 16), the axial displacement (Δ*Z*) between consecutive frames was calculated.

To ensure the accuracy of these measurements at the submicron scale, we accounted for potential microscopic stage drift. A rigid nuclear structure (the centroid of the Lamin B-mCherry signal) was used as a spatial reference point for each frame. The raw centrosome Z-coordinates were normalized to this reference to calculate the net displacement relative to the NE, thereby correcting for global microscope-stage drift. A “stability threshold” of 0.1 μm was established based on the Z-sampling interval used for these datasets. Displacements <0.1 μm were categorized as the operationally defined “stable state,” while displacements >0.1 μm were defined as “active steps.” The stability percentage was calculated as the proportion of total imaging time spent in the stable state. For active intervals, the mean active step was determined by averaging all displacements exceeding the 0.1-μm threshold. Additionally, the average cumulative distance per centrosome was calculated as the sum of all absolute *Z*-displacements over the total observation period.

### Deconvolution

To improve spatial resolution and increase the SNR of the CENP-A signals, raw 3D image stacks were subjected to DeconvolutionLab2 restorative deconvolution^57^. A theoretical Point Spread Function (PSF) was calculated based on the objective lens numerical aperture (NA = 1.4) and the specific emission wavelengths (red: 605 nm; green: 525 nm). The restoration was performed using a Constrained Iterative Richardson-Lucy algorithm with 10 iterations. This setting was optimized to reassign out-of-focus light to its origin while preventing the amplification of background noise or the generation of processing artifacts. This restoration reduced background “haze” enabling sub-pixel localization of CENP-A-labeled centromeric spots for kinematic analysis.

### Image pre-processing and hyperstack construction

Raw 16-bit deconvolved images of CENP-A (mCherry) and TUBG1 (GFP) were imported into Fiji (ImageJ v1.54p). To manage computational load and prevent memory errors, images were loaded as virtual stacks. The resulting image sequences were converted into 4D hyperstacks with dimensions defined by one or two channels (*c* = 1–2), 8–10 Z-slices (z = 8–10), and 49 time points (*t* = 49). To ensure consistency across all experimental replicates–including control, sgRNA, *TUBG*-shRNA–TUBG-GFP-sh-resistant, and cisplatin-treated groups–spatial calibration was standardized to a pixel size of 1.0 and a temporal interval of 1.0 minute.

### Fluorescence intensity normalization

To correct for temporal intensity fluctuations and potential photobleaching—particularly in frames where focal drift or laser instability caused dimming—a linear normalization was applied. Bleach correction was performed using the Simple Ratio method. To avoid artifacts from out-of-focus initial frames, a high-signal “reference frame” (typically *T* = 12) was manually selected as the intensity baseline. The background was subtracted based on the mean intensity of a non-signal region to preserve the signal-to-noise ratio (SNR). For frames with extreme intensity drops, Histogram Normalization (0.3% saturation) was applied to ensure the tracking algorithm could consistently detect centromere signals across the entire 49-minute duration. These processing steps were applied to support consistent CENP-A spot detection and tracking and were not used for the quantitative 3D fluorescence-intensity analyses described above.

### 3D drift correction and projection

Global cellular movement and stage drift were corrected in 3D space using the Correct 3D Drift plugin^58^. Registration was calculated using the CENP-A channel as the reference, with a feature size of 10 pixels and a maximum shift of 50 pixels. The calculated x, y, and z transformation matrices were simultaneously applied to the TUBG channel to maintain strict spatial alignment between the two signals. Following registration, the 8–10 slice Z-stacks were compressed into a 2D time-lapse using a MIP to optimize the detection of centromeric spots.

### Centromere tracking (TrackMate)

Centromere kinematics were quantified using the TrackMate plugin^59^. Automated spot detection was performed using a Laplacian of Gaussian (LoG) detector with an estimated object diameter of 4–6 pixels (adjusted for larger diameters in sgRNA-treated cells) and sub-pixel localization enabled. Spot linking was executed via a linear assignment problem (LAP) Tracker with the following parameters: linking maximum distance: 15.0 pixels; gap-closing maximum distance: 15.0 pixels; and maximum frame gap: two frames.

To ensure data integrity, only tracks persisting for at least 35 frames (~70% of total video duration) were included in the final analysis. Mean velocity and total distance were exported for each track. Velocity values (pixels/min) were converted to micrometers per minute using a standardized scaling factor of 6.4 pixels/μm across all experimental conditions. These parameters were used to identify and track CENP-A-labeled centromeric foci for kinematic analysis and were not used to classify centromeres as TUBG-positive or TUBG-negative.

### High-pressure freezing and immunogold labeling

U2OS cells were seeded to reach 100% confluence on carbon-coated (10 nm) 6 mm sapphire discs (Leica) placed in 12-well plates (Nunc). Cells were cryopreserved using high-pressure freezing (HPM100, Leica) and subsequently processed by freeze substitution (Leica AFS2, Leica) for 48 hours at −90 °C in acetone containing 0.1% uranyl acetate. The samples were then embedded in Lowicryl resin and polymerized at −25 °C for 48 hours.

Ultrathin sections of 60 nm were cut using a Leica Ultracut UC7 ultramicrotome (Leica, Vienna, Austria) and collected on Formvar-coated carbon grids and 200-mesh nickel grids. Sections were pre-incubated for 30 minutes in a pre-incubation buffer (50 mM glycine, 0.1% sodium borohydride [NaBH_4_], 0.05 M Tris, pH 7.4, 0.1% Triton X-100).

For immunogold labeling, sections were incubated for 2 hours at room temperature with a polyclonal anti-TUBG antibody diluted in TBST (0.05 M Tris, pH 7.4, 0.1% Triton X-100, 1% BSA). This was followed by a 1-hour incubation with gold-conjugated protein A (1:100, 10 nm gold; Agar Scientific, Essex, UK) in TBST. The final staining step was performed with 0.5% uranyl acetate (filtered) for 10 minutes.

Images were captured using a FEI Tecnai Spirit transmission electron microscope (FEI, Hillsboro, Oregon, USA) equipped with an SIS Veleta (2 × 2k) CCD camera (Olympus, Tokyo, Japan).

### Sucrose gradient ultracentrifugation

U2OS cells were cultured and prepared for analysis of centrosome- and centromere-associated material as described above. To preserve centrosome-associated material under stress conditions, cells were treated with 5 µM cisplatin for 24 h. Prior to harvesting, actin filaments were destabilized by the addition of 5 µg/ml cytochalasin B for 20 min at 37 °C, while TUBG structures were stabilized by the addition of 50 nM L12^43,60^. Cells were then fixed with 1% formaldehyde for 10 min at room temperature; the reaction was subsequently quenched by the addition of 0.125 M glycine for 5 min. Following two washes with PBS, cells were harvested via trypsinization and collected in ice-cold PBS supplemented with 1 mM PMSF (phenylmethanesulfonyl fluoride).

The cell pellet (50 × 10^6^ cells) was resuspended in 4 ml of Lysis Buffer (LB) (10 mM Tris–HCl [pH 7.5], 10 mM NaCl, 3 mM MgCl_2_, 0.5% IGEPAL, supplemented with 1 mM PMSF) and incubated on ice for 15 min. Intact nuclei were pelleted by centrifugation at 1300 × *g* for 5 min at 4 °C. The resulting supernatant was reserved as the cytosolic fraction. Isolated nuclei were resuspended in 400 µl of Nuclear Lysis Buffer (NLB) (10 mM Tris–HCl [pH 8.0], 150 mM NaCl, 1 mM CaCl_2_, 1.5 mM MgCl_2_, 0.5% IGEPAL) and incubated under rotation at room temperature for 25 min. Chromatin was digested by the addition of 25 U/ml DNase I for 5 min^60^. To stabilize protein complexes and prevent dissociation, EDTA was then added to a final concentration of 10 mM. The lysate was then cleared of debris by centrifugation at 10,000 × *g* for 5 min at 4 °C.

The cleared nuclear or cytosolic lysates were layered onto a discontinuous sucrose gradient consisting of 70% (500 µl), 50% (500 µl), and 40% (300 µl) sucrose solutions prepared in Dilution Buffer (DB). Samples were subjected to ultracentrifugation at 120,000 × *g* for 45 min at 4°C.

Fractions were collected from the top of the gradient. An initial 1.8 ml volume was collected as Fraction 1, followed by subsequent 200 µl fractions (Fractions 2–8). To identify fractions enriched in centromere-associated material, Western blotting was performed using an anti-CENP-A antibody as a biochemical marker for centromeric enrichment. For centrosome sedimentation from the cytosolic fraction, the sample was initially layered over a 60% sucrose cushion (400 µl) and centrifuged at 10,000 × *g* for 80 min before subsequent gradient loading.

The CENP-A-positive fractions were diluted at least five-fold in DB to reduce viscosity and selected for imaging. Samples were incubated with primary antibodies (anti-TUBG, anti-α-tubulin, and anti-centromere) for 1 h under rotation, followed by a 1-h incubation with fluorophore-conjugated secondary antibodies.

For fluorescence microscopy, microscope slides were pre-coated with 0.1% gelatin. A centrifuge adapter (Thermo Shandon Cytospin 2) was used to layer the labeled sample over a 30% sucrose cushion (1:1 ratio). Samples were centrifuged using the Thermo Shandon Cytospin 2 at 1500 × *g* for 10 min to sediment the isolated material onto the glass surface. Slides were mounted and stored for subsequent imaging.

### ChIP-seq analysis

The raw sequence data and processed files used for analyzing the distribution of TUBG-associated ChIP-seq signal in MCF10A and U2OS cells are publicly available in the NCBI Gene Expression Omnibus (GEO) database (http://www.ncbi.nlm.nih.gov/geo). ChIP-seq peaks were detected as previously described^4,22^. Accession numbers:

**MCF10A cells**: GSM2884568_gTub_no_shRNAi_1h^22^, GSM2884570_gTub_no_shRNAi_2h^22^, GSM2884576_gTub_with_shRNAi_1h^22^, GSM2884578_gTub_with_shRNAi_2h^22^.

**U2OS cells**: GSE148798_gTub_0h^4^, GSE148798_gTub_3h^4^, GSE148798_gTub_5h^4^, GSE148798_gTub_7h^4^, GSE148798_gTub_9h^4^, GSE148798_gTub_G0_G1^4^, GSE173732_TUBG_3h_U2OS^4^, GSE173732_TUBG_3h_shgtub^4^, GSE173732_TUBG_7h_U2OS^4^, GSE173732_TUBG_7h_shgtub^4^, GSE173732_TUBG_9h_U2OS^4^, GSE173732_TUBG_9h_shgtub^4^, GSE173732_TUBG_NS_U2OS^4^, GSE173732_TUBG_NS_shgtub^4^.

The distribution of ChIP-seq peaks across the genome was plotted with karyoploteR^61^. Distances from centromeres were calculated using genomic coordinates retrieved from the UCSC Genome Browser cytoband track (Supplementary Table 1) and the distanceToNearest function from GenomicRanges. All genomic distances are reported in base pairs (bp). For each peak, the distance to the centromere was calculated as the distance between their closest coordinates. Specifically, for peaks located 5’ of the centromere, the distance was measured between the 3’ end of the peak and the 5’ end of the centromere. Conversely, for peaks located 3’ of the centromere, the distance was measured between the 3’ end of the centromere and the 5’ end of the peak. All downstream analyses were performed in R^61^ using the following Bioconductor packages: GenomicRanges and ChIPseeker. Genomic annotations were from BSgenome.Hsapiens.UCSC.hg19 and TxDb.Hsapiens.UCSC.hg19.knownGene. Chromosome plots were generated using karyoploteR^61^ and other plots using ggplot2^62^. Random regions were generated using the following code: random_regions <− createRandomRegions(nregions = 5000, length.mean = 200, length.sd = 20, genome = “hg19”, non.overlapping = TRUE) from regioneR^63^.

To complement peak-based analysis, we performed signal-based centromere-centered analysis of normalized ChIP-seq coverage. For this purpose, BAM files from replicates at each time point were merged prior to analysis. ChIP-seq signal was binned (bin size = 10 bp), and coverage tracks were computed after scaling to 1× genomic coverage, excluding reads overlapping ENCODE blacklist regions^64^, using deepTools v. 3.5.6^65^. Centromere coordinates were derived from the UCSC cytoband track (feature type “acen”). Summary profile plots and heatmaps of signal flanking centromeres were generated using deepTools v 3.5.6, based on signal summarized in bins of 2 kb. Centromere intervals were scaled to a uniform length of 1 kb, and 5 kb flanking regions were added on both sides. Summary profiles represent the mean normalized signal across all centromeres for each time point, whereas heatmaps display the signal at individual centromeres while preserving the same row order across conditions. Genomic views were plotted using pyGenomeTracks^66^ with log10-transformed signal values, and data ranges were harmonized across time points to enable direct comparison. This approach enables visualization of normalized TUBG-associated signal across annotated centromere regions independently of peak calling, which may underestimate signal in highly repetitive centromeric regions.

### Statistics and reproducibility

Data are presented as mean ± standard deviation (SD). Normality of the data was evaluated using the Shapiro–Wilk test. Because the majority of datasets (e.g., centromere intensity and centrosome displacement) exhibited non-Gaussian distributions, non-parametric statistical methods were employed. The Wilcoxon matched-pairs signed-rank test was utilized for paired analyses, including the paired comparison of TUBG and Lamin B fluorescence intensities within the same centromere-defined ROI in Fig. 1e and the assessment of adjacent Cas9-GFP-*TUBG1*-sgRNA-expressing and non-expressing cells within the same imaging field (internal controls). The Mann–Whitney *U* test was used for comparisons between independent experimental groups (e.g., untreated vs. cisplatin-treated populations). Paired t-tests were used for paired datasets that satisfied the normality assumptions, as specified in the relevant figure legends. Unless otherwise stated, all statistical tests were two-sided. Exact *P* values are reported in the corresponding figure legends where available. *P* values displayed by GraphPad Prism as *P* < 0.0001 are reported as such. Statistical significance was determined as follows: *P < 0.05, **P < 0.01, ***P < 0.001, ****P < 0.0001. All statistical analyses and visualizations were performed using GraphPad Prism (version 10.05). The primary unit of analysis varied according to the experiment and included individual cells, segmented centromere-defined volumes, centromere tracks, centrosomes, or image frames. The corresponding unit of analysis and the number of independent biological replicates are specified in the relevant figure legends and Results sections.

### Use of artificial intelligence

During manuscript revision, the authors used ChatGPT (OpenAI) to assist with language editing, formatting, and manuscript-organization suggestions. ChatGPT was not used to generate experimental data, figures, or images, or to perform the statistical analyses reported in this study. All suggestions were critically reviewed and verified by the authors, who take full responsibility for the final content of the manuscript.

### Reporting summary

Further information on research design is available in the Nature Portfolio Reporting Summary linked to this article.

## Supplementary information

Transparent Peer Review file

Supplemental information

Description of additional Supplementary files

Supplementary Data

Supplementary video 1

Supplementary Video 2

Supplementary Video 3

Supplementary Video 4

Supplementary Video 5

Supplementary Video 6

Supplementary Video 7

Supplementary Video 8

Reporting Summary

## Data Availability

The raw sequence data and processed files used for the ChIP-seq analyses are publicly available in the NCBI GEO under accession codes GSE148798, GSE173732, GSM2884568, GSM2884570, GSM2884576, and GSM2884578. Numerical source data underlying the graphs in Figs. 1–3 and 5–9 and Supplementary Figs. 1 and 3, together with original blots for Fig. 7h and Supplementary Fig. 2, can be obtained from Supplementary Data. The additional data generated in this study include confocal and Airyscan fluorescence microscopy images, live-cell time-lapse image stacks and videos, electron microscopy images, immunoblot data, and the associated quantitative measurements. These data are currently stored on secure institutional servers at Lund University. Data supporting the findings of this study that are not included in the article or its Supplementary Information are available from the corresponding author, Maria Alvarado-Kristensson (maria.alvarado-kristensson@med.lu.se), upon reasonable request.

## Code availability

No custom code was generated for this study. All analyses were performed using publicly available software and packages, with the software versions and analysis parameters described in the Methods.
