# Supplementary material for: The TUBG meshwork is associated with centromere dynamics and micronuclear organization
Source: Commun Biol. 2026 Sep 16;9:1206. doi: 10.1038/s42003-026-10880-y (PMC13582872; doi:10.1038/s42003-026-10880-y)
Supplement: Supplementary file 1 [file 42003_2026_10880_MOESM13_ESM.pdf]

nature portfolio

Corresponding author(s): *Double-anonymous peer review submissions: write DAPR and your manuscript number here instead of author names.*

Last updated by author(s): *YYYY-MM-DD*

Reporting Summary

Nature Portfolio wishes to improve the reproducibility of the work that we publish. This form provides structure for consistency and transparency in reporting. For further information on Nature Portfolio policies, see our Editorial Policies and the Editorial Policy Checklist.

Statistics

For all statistical analyses, confirm that the following items are present in the figure legend, table legend, main text, or Methods section.

n/a	Confirmed	
☐	☒	The exact sample size (*n*) for each experimental group/condition, given as a discrete number and unit of measurement
☒	☐	A statement on whether measurements were taken from distinct samples or whether the same sample was measured repeatedly
☐	☒	The statistical test(s) used AND whether they are one- or two-sided<br>*Only common tests should be described solely by name; describe more complex techniques in the Methods section.*
☒	☐	A description of all covariates tested
☒	☐	A description of any assumptions or corrections, such as tests of normality and adjustment for multiple comparisons
☐	☒	A full description of the statistical parameters including central tendency (e.g. means) or other basic estimates (e.g. regression coefficient) AND variation (e.g. standard deviation) or associated estimates of uncertainty (e.g. confidence intervals)
☒	☐	For null hypothesis testing, the test statistic (e.g. *F*, *t*, *r*) with confidence intervals, effect sizes, degrees of freedom and *P* value noted<br>*Give P values as exact values whenever suitable.*
☒	☐	For Bayesian analysis, information on the choice of priors and Markov chain Monte Carlo settings
☒	☐	For hierarchical and complex designs, identification of the appropriate level for tests and full reporting of outcomes
☐	☒	Estimates of effect sizes (e.g. Cohen's *d*, Pearson's *r*), indicating how they were calculated

Our web collection on statistics for biologists contains articles on many of the points above.

Software and code

Policy information about availability of computer code

Data collection	Zeiss LSM 700 Axio Observer microscope, Nikon A1RHD, a Zeiss LSM 880 with Airyscan, and Zeiss LSM 900 with Airyscan 2, Fei Tecnai Spirit transmission electron microscope (FEI, Hillsboro, Oregon, USA) equipped with an SIS Veleta (2 × 2k) CCD camera (Olympus, Tokyo, Japan).
Data analysis	ImageJ (Fiji, version 2.16.0/1.54p), Zen, GraphPad Prism (Version 10.05), karyoploteR, GenomicRanges, ChIPseeker, ggplot2, regioneR, deepTools, deepTools (v3.5.6), pyGenomeTracks,

For manuscripts utilizing custom algorithms or software that are central to the research but not yet described in published literature, software must be made available to editors and reviewers. We strongly encourage code deposition in a community repository (e.g. GitHub). See the Nature Portfolio guidelines for submitting code & software for further information.

Data

Policy information about availability of data

All manuscripts must include a data availability statement. This statement should provide the following information, where applicable:

- Accession codes, unique identifiers, or web links for publicly available datasets
- A description of any restrictions on data availability
- For clinical datasets or third party data, please ensure that the statement adheres to our policy

material request should be addressed to the corresponding author: Maria Alvarado-Kristensson
Maria.alvarado-kristensson@med.lu.se

Research involving human participants, their data, or biological material

Policy information about studies with human participants or human data. See also policy information about sex, gender (identity/presentation), and sexual orientation and race, ethnicity and racism.

Reporting on sex and gender	From a gender perspective, we are committed to ensuring inclusivity and gender equity in all aspects of the research, including the collection of patient samples, data analysis, and the interpretation of results. This includes acknowledging potential gender differences in disease progression and response to treatment. Efforts are made to ensure equal representation and avoid bias, allowing for more comprehensive and accurate findings that can benefit all genders. Through these measures, we ensure that the research is conducted responsibly, with respect for ethical guidelines, and with consideration for gender diversity.
Reporting on race, ethnicity, or other socially relevant groupings	NA
Population characteristics	NA
Recruitment	Cancer patients
Ethics oversight	This research project investigates the role of TUBG in cancer. The project includes both cell models and analyses of tissue samples from renal carcinoma patients. Careful ethical considerations are required for this work. The use of human tissue samples follows the guidelines for biomedical research and is based on approval from ethical review boards. All samples are handled in accordance with the Helsinki Declaration, and informed consent is obtained from patients or their relatives. Anonymization and confidentiality are ensured to protect individuals' privacy. The results are used solely for scientific purposes, and no genetic data are linked to individual patients. Primary human kidney epithelial and renal carcinoma cells were isolated after informed consent from participants. Cells were cultured from patient nephrectomies specimens, and all analyses were performed under ethical approval from Lund University ethical committee (LU680-08 and LU 289-07). Tumors were classified as clear cell renal cell carcinoma (ccRCC) by an experienced pathologist. Normal kidney samples were collected from healthy cortex regions furthest from the tumor site.

Note that full information on the approval of the study protocol must also be provided in the manuscript.

Field-specific reporting

Please select the one below that is the best fit for your research. If you are not sure, read the appropriate sections before making your selection.

☒ Life sciences ☐ Behavioural & social sciences ☐ Ecological, evolutionary & environmental sciences

For a reference copy of the document with all sections, see nature.com/documents/nr-reporting-summary-flat.pdf

Life sciences study design

All studies must disclose on these points even when the disclosure is negative.

Sample size	Once the methods were optimized, that is, that all internal controls were working, the experiments were repeated according to size indicated in the manuscript.
Data exclusions	Experiments where the internal controls were not working were excluded.
Replication	All experiments with working internal controls were repeated to assure reproducibility in independent experiments as indicated in the manuscript.
Randomization	NA
Blinding	NA

Reporting for specific materials, systems and methods

We require information from authors about some types of materials, experimental systems and methods used in many studies. Here, indicate whether each material, system or method listed is relevant to your study. If you are not sure if a list item applies to your research, read the appropriate section before selecting a response.

Materials & experimental systems

n/a	Involved in the study
☐	☒ Antibodies
☐	☒ Eukaryotic cell lines
☒	☐ Palaeontology and archaeology
☒	☐ Animals and other organisms
☒	☐ Clinical data
☒	☐ Dual use research of concern
☒	☐ Plants

Methods

n/a	Involved in the study
☐	☒ ChIP-seq
☒	☐ Flow cytometry
☒	☐ MRI-based neuroimaging

Antibodies

Antibodies used

anti-gamma-tubulin, anti-anti-centromere protein, anti-α-tubulin, anti-Nuclear Pore Complex, anti-SAS6, β3-tubulin, anti-pericentrin 1, anti-GCP2, anti-lamin B and anti-phospho-Ser131-TUBG (1:160).

Validation

Antibodies against gamma-tubulin were validated with sg-TUBG and sh-TUBG. In addition, shTUBG (which decreased the levels of gamma-tubulin by 50% was also used to assure the specificity of the antibodies used in the Chip-seq. Antibodies against phosphorylated Serine 131 were validated with treatment with phosphatase and a total anti-gamma-tubulin antibody, as well as in vitro kinase assays using non-phosphorylable and phosphorylable mutants (DOI: 10.1038/ncb1921)

Eukaryotic cell lines

Policy information about cell lines and Sex and Gender in Research

Cell line source(s)

ACC and H9-NCS (a gift from Dr. Karayan-Tapon).

Authentication

We assay their morphology, size and protein expression. In addition, the different cell lines require different growth media. The authentication of H9-NSC was carried out by eurofins genomics Europe Applied genomics, by using a PCR-single-locus-technology.

Mycoplasma contamination

Yes, all cell lines are routinely tested for micoplasma contamination.

Commonly misidentified lines (See ICLAC register)

The effects we study are not cell line specific. We study how TUBG nucleates centromeres in mammalian cells.

Plants

Seed stocks

NA

Novel plant genotypes

NA

Authentication

NA

ChIP-seq

Data deposition

☒ Confirm that both raw and final processed data have been deposited in a public database such as GEO.

☒ Confirm that you have deposited or provided access to graph files (e.g. BED files) for the called peaks.

Data access links
May remain private before publication.

The raw sequence data and processed files used for mapping the location of TUBG in MCF10 and U2OS cells are publicly available in the NCBI Gene Expression Omnibus (GEO) database (http://www.ncbi.nlm.nih.gov/geo). ChIP-seq peaks were detected as previously described (DOI: 10.1038/s42003-018-0037-3 and 10.1038/s42003-021-02280-1). Accession numbers:
MCF10A cells: GSM2884568_gTub_no_shRNAi_1h, GSM2884570_gTub_no_shRNAi_2h,
GSM2884576_gTub_with_shRNAi_1h, GSM2884578_gTub_with_shRNAi_2h.
U2OS cells: GSE148798_gTub_0h, GSE148798_gTub_3h, GSE148798_gTub_5h, GSE148798_gTub_7h, GSE148798_gTub_9h, GSE148798_gTub_G0_G1, GSE173732_TUBG_3h_U2OS, GSE173732_TUBG_3h_shgtub, GSE173732_TUBG_7h_U2OS, GSE173732_TUBG_7h_shgtub, GSE173732_TUBG_9h_U2OS, GSE173732_TUBG_9h_shgtub, GSE173732_TUBG_NS_U2OS,

GSE173732_TUBG_NS_shgtub.

Files in database submission: *Provide a list of all files available in the database submission.*

Genome browser session (e.g. UCSC): *Provide a link to an anonymized genome browser session for "Initial submission" and "Revised version" documents only, to enable peer review. Write "no longer applicable" for "Final submission" documents.*

Methodology

Replicates: *Describe the experimental replicates, specifying number, type and replicate agreement.*

Sequencing depth: *Describe the sequencing depth for each experiment, providing the total number of reads, uniquely mapped reads, length of reads and whether they were paired- or single-end.*

Antibodies: *Describe the antibodies used for the ChIP-seq experiments; as applicable, provide supplier name, catalog number, clone name, and lot number.*

Peak calling parameters: *Specify the command line program and parameters used for read mapping and peak calling, including the ChIP, control and index files used.*

Data quality: *Describe the methods used to ensure data quality in full detail, including how many peaks are at FDR 5% and above 5-fold enrichment.*

Software: *Describe the software used to collect and analyze the ChIP-seq data. For custom code that has been deposited into a community repository, provide accession details.*
