# Supplementary material for: The TUBG meshwork is associated with centromere dynamics and micronuclear organization
Source: Commun Biol. 2026 Sep 16;9:1206. doi: 10.1038/s42003-026-10880-y (PMC13582872; doi:10.1038/s42003-026-10880-y)
Supplement: Supplementary file 2 [file 42003_2026_10880_MOESM1_ESM.pdf]

nature portfolio

Peer Review File

The TUBG meshwork is associated with centromere dynamics and micronuclear organization

Corresponding Author: Dr Maria Alvarado Kristensson

This file contains all reviewer reports in order by version, followed by all author rebuttals in order by version.

Version 0:

Reviewer comments:

Reviewer #1

(Remarks to the Author)
In this manuscript, Muteb et al. investigate the relationship between the γ-tubulin meshwork and centromeres, starting from ChIP-Seq analysis and extending into cellular and imaging studies. The study addresses a timely and interesting question, and the data suggest intriguing links between γ-tubulin, centrosomes, and centromere organization in interphase. Overall, this study presents valuable data on γ-tubulin and centromere organization. The work convincingly shows γ-tubulin remodelling under stress, centromere organization mediated by phosphorylation-dependent nucleation, and micronuclei involvement. Indeed, the dynamic localization of γ-tubulin into micronuclei and its association with DNA replication is intriguing and raises new questions. However, the manuscript in its current form contains claims that are insufficiently supported by the presented data. The claims regarding nuclear–cytosolic connections and γ-tubulin–mediated control of centromeres are not well established. Additional quantification, improved presentation, and further experiments are needed to substantiate the central hypothesis.

Main concerns that need to be addressed:

1. Nearly 40% of references are self-citations from the corresponding author, which should be reduced.
2. It is not clear throughout the text how the authors distinguish γ-tubulin meshwork from centrosomes. This distinction should be explicitly stated and consistently addressed.
3. I have found some overstated claims:
- The proposed mechanism that cytosolic γ-tubulin controls centromeres is not sufficiently supported by the data. Additional experiments suggested below
- The claim that centrosomes participate in centromere nucleation cannot be made based only on fixed-cell immunolabeling (Figure 5).
- Data presented on γ-tubulin–centrosome dynamics demonstrate association but not direct interaction or organizational control of centromeres.
4. Data presentation:
- Table 1: Units of distance to the centromere must be specified.
- Figure 1C: Axes lack units (density and distance). The MCF10A plot should be zoomed into the relevant density range (around 1e–07) to highlight the three reliable peaks.
- Figure 3D: Immunoelectron microscopy panels are too small to appreciate γ-tubulin labelling or chromatin. I suggest to enlarge panels. In addition, tomograms of 250 nm sections are needed to clarify direct associations.
- Movie 1: numbering centromeres would improve clarity of the dynamic interactions.

Points Requiring Additional Analysis or Experiments:

- For ChIP-Seq data, include a clear statement on co-localization of γ-tubulin and centromeres, specifying whether a distinct pattern is observed.
- Quantify the percentage of centrosomes in non-differentiated neuronal cells that contact the nuclear envelope (Figure 3A), including number of cells analysed and error margins.
- To substantiate claims of direct interaction and structural contribution of γ-tubulin to centromere organization, correlative cryo-electron tomography is strongly recommended.
- The assertion that γ-tubulin tubules connect the nuclear compartment to microtubules requires higher-resolution imaging

(again, correlative cryo-tomography would be appropriate).

Reviewer #2

(Remarks to the Author)
This manuscript addresses an ambitious and thought provoking hypothesis that the TUBG meshwork not only bridges the cytoplasm and the nucleus but also provides a structural scaffold for the establishment of functional centromeres. While the premise is intriguing, I find the current evidence insufficient in both depth and rigor to substantiate such a far reaching claim. A major concern is that many of the key findings rely primarily on microscopy based observations. To ensure robustness and reproducibility, comprehensive quantitative analyses and appropriate statistical validation should be included throughout. In addition, the central assertion, that the cytoplasmic TUBG network penetrates the nuclear envelope and functions within the nuclear compartment, cannot be convincingly supported by imaging alone. Biochemical approaches would be essential to strengthen this conclusion.For these reasons, which are detailed further in my specific comments below, I do not recommend acceptance of the manuscript in its present form.

Figures 1D and 1E
Quantitative analysis is required to show the proportion of cells exhibiting the described phenotype. Statistical evaluation should subsequently be performed to support the validity of these observations.

Figure 2A
Quantitative analysis is also necessary to substantiate the described results.

Figure 2B
The number of cells counted and whether their expression levels were comparable should be clearly indicated to ensure data reliability.

The authors show that centromere signal intensity increases in cells with reduced TUBG expression compared with Cas9-GFP-TUBG1-sg non-expressing cells (Fig. 1C or p16). However, this observation alone does not establish that TUBG functions as a stabilizing factor required to maintain proper centromere organization, nor that TUBG prevents excessive condensation or aberrant centromere assembly during interphase.
It also remains unclear whether the increased centromere signal represents an abnormal structural state or whether it is associated with any functional defects, such as impaired chromosome segregation or mitotic progression.

Figures 3A-C
All panels require quantitative analysis to substantiate the presented findings.

Supplementary Figure 1A and Movie 1
The authors describe an observation in which the centrosome moves from the imaging plane toward the nuclear envelope (labeled with mCherry-NUP50 and Hoechst) and reappears within a few minutes. This behavior should be quantitatively analyzed. It also remains unclear whether this represents a biologically meaningful event rather than a coincidental or technically induced observation.

Supplementary Figure 1B
Quantitative analysis is required to support the validity of the observations.

Page 22
The statement, “interacting with centromeres, which resulted in distinct scenarios of centrosome-centromere interactions,” is not substantiated by the presented data. No direct evidence of centrosome-centromere interaction is provided, and this claim should therefore be revised or removed.

The authors also state, “centrosomes interacted with a single centromeric focus, which subsequently underwent division into two independent centromeric foci.” This centrosome-centromere interaction should be quantitatively analyzed to indicate the percentage of centromeres exhibiting this behavior. It should also be clarified whether the same centromeres consistently undergo this process or if it occurs randomly. A comparison between centromeres that interact with centrosomes and those that do not would help determine whether meaningful differences exist. From the presented data, the majority of centromeres do not appear to engage in such interactions, raising questions about the biological significance of this observation.

The authors further propose that “this dynamic interplay highlights a potential role for the TUBG-associated centrosome in actively facilitating the reorganization or maturation of centromeres during interphase.” However, it is not evident from the data that this conclusion is supported. As a first step, the authors should provide quantitative information on the proportion of cells exhibiting this phenomenon to establish its reproducibility and relevance.

Page 23
The statement, “g-tubules formed physical connections with multiple centromeres,” is not convincingly demonstrated. The positional proximity of TUBG and centromeres observed microscopically does not necessarily indicate a direct interaction. Additional analyses, ideally using biochemical approaches, are needed to confirm that this spatial relationship reflects a functional connection. Moreover, it is unclear whether all centromeres are connected in this manner or how many such connections are statistically significant.

The subsequent claim that “the dynamic TUBG meshwork, including both centrosome-associated pools and nucleated g-tubules, serves as an active scaffold for interphase centromere organization” is not sufficiently supported by the current data.

Page 26
The statement, “During this transition, centrosomes localized to chromatin regions where centromeres assembled,” requires statistical validation. Furthermore, Figure 5A is not well organized, which complicates data interpretation.
The claim, “Instead, centromere nucleation appeared to occur at the centrosomes, suggesting that centrosomes, through their association with the TUBG meshwork, act as docking sites for centromeres,” lacks clear supporting evidence in the presented data.

Figs. 5A-B also require quantitative analysis to demonstrate statistical significance. The authors should clarify how this differs from random or coincidental events.
The statement, “centromeres were arranged in ring-like structures around a single g-tubule and at intersections where two g-tubules crossed,” raises additional questions: what is the biological significance of these intersections, and what specific structural or biochemical properties characterize them?

Figures 6A-C
Statistical analyses are required to support the presented findings.

Page 31
The statements are difficult to interpret without an explanation of how these micronuclei are formed. The authors should clarify whether tahey arise from mitotic defects, cellular senescence, or other processes. Without this context, the biological significance of the observation cannot be evaluated.

Figure 7 is confusing and does not clearly convey the intended message.

The statement, “Furthermore, an increased number of cells with g-tubules were observed upon treatment with either cisplatin or rotenone, indicating a stress-induced alteration in how genetic material is packaged and handled within these structures,” attributes the observed effects solely to g-tubules. It would be more appropriate for the authors to discuss alternative explanations or contributing factors.

The statement, “The observed dynamic organization of centromeres by g-tubules in both nuclear and cytoplasmic compartments under rotenone and cisplatin treatment highlights a crucial, conserved role for the TUBG network in maintaining genome integrity and responding to various cellular insults,” appears overstated. The data presented do not convincingly demonstrate that the TUBG meshwork is actively regulated in response to cellular stress.

Page 34
Finally, the statement, “Given that cellular stress and chromosome mis-segregation can lead to the formation of micronuclei,” leaves unresolved whether micronuclei formation results from abnormalities in interphase TUBG-meshwork-mediated centromere organization or simply from previously reported mitotic centromere-centrosome defects. This distinction should be clearly addressed.

Version 1:

Reviewer comments:

Reviewer #1

(Remarks to the Author)
The authors have substantially revised the manuscript and have clearly invested considerable effort in addressing many of the concerns raised during the previous review round. The manuscript is now better organized, the distinction between centrosomal and non-centrosomal TUBG structures is more clearly described, additional quantitative analyses have been incorporated, and several overstated claims have been moderated. The inclusion of additional quantitative measurements of centrosome–nuclear envelope association, and biochemical fractionation experiments has improved the overall quality of the work.

The study addresses an interesting and potentially important question regarding the role of the γ-tubulin meshwork in centromere organization and nuclear architecture. However, several conceptual and methodological concerns remain unresolved. In particular, the evidence presented continues to support association and correlation more strongly than direct interaction or mechanistic control. While I believe the manuscript has improved significantly, additional revisions are required before the conclusions can be fully supported by the available data.

Major Comments

1. Evidence for Direct Interaction Remains Indirect
One of my principal concerns from the previous review was the distinction between spatial association and direct interaction. The authors now provide biochemical co-fractionation data showing that TUBG-containing structures and centromeric DNA

remain associated during fractionation. While this is an interesting observation, co-fractionation alone cannot establish direct molecular interaction. Stable co-isolation may result from indirect association through large multiprotein assemblies, chromatin scaffolds, nuclear matrix components, or incomplete dissociation during extraction.
Throughout the revised manuscript, the language has been moderated in some sections, but statements implying direct interaction, physical tethering, or structural anchoring remain stronger than warranted by the evidence presented. The biochemical data support stable association but not direct molecular interaction.
I therefore recommend that the authors consistently use terminology such as “association”, “co-fractionation”, or “structural proximity” unless additional biochemical evidence (e.g., crosslinking, proximity labelling, co-immunoprecipitation under defined conditions, or ultrastructural visualization) is provided.
2. Evidence for Functional Control of Centromere Organization Remains Limited
The authors propose that TUBG acts as a “mechanical buffer”, “kinematic scaffold”, and regulator of centromere homeostasis. While the mobility data following TUBG depletion are intriguing, they do not by themselves demonstrate organizational control.
The central observation remains that TUBG depletion alters centromere fluorescence intensity and reduces centromere mobility. These findings are compatible with several interpretations, including indirect effects on nuclear architecture, chromatin mechanics, cell-cycle progression, DNA damage signalling, or centromere organization itself.
The revised manuscript continues to present a model in which TUBG actively controls centromere homeostasis. In my opinion, the data currently support a more conservative conclusion: TUBG contributes to the structural environment associated with centromere positioning and mobility.
The distinction between contribution and control is important and should be reflected more carefully throughout the Discussion.
3. Interpretation of Centromere “Compaction”
I remain unconvinced by the interpretation that increased centromere fluorescence intensity directly reflects centromere compaction or condensation.
The authors use the terms “compaction”, “condensation”, and “stalling” interchangeably in several sections. However, fluorescence intensity measurements alone do not provide direct information regarding chromatin density or volumetric compaction.
Demonstrating compaction would require complementary measurements, such as: volumetric reconstruction of individual centromeres, changes in centromere volume, chromatin ultrastructure analysis, or orthogonal biochemical measurements. Unless such data are available, I recommend describing the phenotype more neutrally as increased fluorescence intensity associated with reduced mobility.
4. Cryo-ET Concerns Are Not Fully Addressed
I appreciate the authors’ explanation regarding the limitations of cryo-electron tomography for studying dynamic processes. I agree that cryo-ET cannot directly address kinetic behaviour. However, this was not the primary motivation behind my suggestion. The rationale was to obtain higher-resolution structural evidence supporting the proposed physical connections between γ-tubules, centrosomes, centromeres, and the nuclear compartment.
The authors correctly point out the volumetric limitations of cryo-ET. Nevertheless, the absence of such structural validation means that several architectural conclusions remain inferential. The new Airyscan analyses improve the visualization of these structures but do not provide the molecular resolution required to distinguish direct contacts from close spatial proximity.
I therefore do not consider this issue resolved, although I acknowledge that it may be beyond the scope of the current study.
5. ChIP-seq Interpretation
The additional centromere-centered analyses strengthen the manuscript and demonstrate reproducible TUBG-associated signal around annotated centromeric regions.
However, the authors should be careful not to overinterpret these data. ChIP-seq demonstrates chromatin association but does not distinguish whether TUBG directly binds centromeric chromatin, associates through intermediary proteins, or reflects broader chromatin-domain organization. The current interpretation should therefore remain descriptive.

Minor Comments

1. Reference Usage
The revised manuscript has reduced the proportion of self-citations and the situation has improved considerably. However, several instances remain where review articles are cited instead of primary literature, particularly when supporting specific experimental observations. The reference list should be carefully re-examined to ensure that primary evidence is cited whenever possible. For example:
Page 4 “Altered expression and localization of TUBG have been documented in several cancers, including gliomas, medulloblastomas, glioblastomas, myelomas, hepatocellular carcinoma, non-small cell carcinoma, and breast cancer.” The statement is currently supported by a self-citation to a review article. More appropriate primary references are available and should be cited instead. Examples include DOI: 10.1097/01.jnen.0000229235.20995.6e, DOI: 10.1007/s11064-007-9321-1, and DOI: 10.1002/jcp.22077.
Page 6 “U2OS cells were transfected with X-fect (Takara Bio Europa, Cat. No. 631317) according to the manufacturer’s instructions.” If the transfection was performed strictly according to the manufacturer’s protocol, the rationale for including a self-citation is unclear. Please clarify whether the cited reference describes modifications to the standard protocol or remove the citation if it is not required.
Page 22
“In our previously published work, we demonstrated that cellular TUBG forms various structural arrangements, including distinctive thread-like γ-strings, measuring 4 to 6 nm in diameter.” Reference 37 does not support this statement. The cited work is focused on the identification and isolation of TUBG–pericentrin complexes and does not describe the formation or characterization of γ-strings.

“Notably, γ-strings play a key role in the transport of cytosolic PCNA to replication origins.” Reference 38 does not appear to support this statement. The cited study focuses on the role of the chaperonin CCT in the formation of γ-tubulin filaments and does not address the transport of cytosolic PCNA by γ-strings.
“In H9-NSC cells, we additionally observed TUBG-foci.” Reference 39 is a review article and does not appear to provide direct experimental evidence supporting this specific observation. A primary reference would be more appropriate.
Page 34.“This network includes not only the previously noted γ-strings but also γ-tubules, which are cytosolic fibers with diameters between 20 and 25 nm.” Reference 45 does not appear to support this statement. The cited work addresses the targeting of γ-tubulin to microtubule domains rather than the formation or characterization of γ-strings or γ-tubules.
Page 38. “The functional nature of this docking is further substantiated in U2OS cells treated with 5 µM cisplatin, a DNA-damaging agent that induces genomic stress.” This statement would benefit from a direct reference describing cisplatin-induced DNA damage and genomic stress, such as: Wang D, Lippard SJ. Cellular processing of platinum anticancer drugs. Nat Rev Drug Discov. 2005;4:307–320. The current self-citation (reference 4) is not the most appropriate source for this well-established concept.
2. Statistical Reporting
The manuscript now reports the number of cells and experiments more consistently. However, for all quantitative analyses, the authors should clearly distinguish: number of biological replicates, number of cells, number of tracks, and number of segmented centromeres.
This distinction remains difficult to follow in several figures.
3. Graphical Presentation
The issues previously raised regarding colour schemes and symbol coding remain only partially resolved. In several figures, it remains difficult to determine: which symbols correspond to which treatment, which data points are paired, and how the statistical comparisons were performed. For example:
Figure 2A. Why is the centromere signal displayed in red in the CPM panels while it is shown in purple throughout the remainder of the figure set? Apart from making interpretation more difficult for colour-blind readers, the sudden change in colour coding is confusing and should be justified or standardized.
Figure 2B. Why was a Wilcoxon matched-pairs signed-rank test used instead of a paired t-test? Please justify the choice of statistical test. The graph symbols are difficult to interpret. The use of filled and open circles together with upward- and downward-pointing triangles is not adequately explained. It is unclear which symbols correspond to cisplatin-treated and untreated samples, and which data points are paired in the statistical analysis.
Figure 3C. As in Figure 2A, the red-green colour coding is not clearly explained and is difficult to interpret. A more consistent colour scheme throughout the manuscript would improve readability.
Figure 7F. As in Figure 2B, the graphical symbols are not sufficiently explained. It is unclear which symbols correspond to cisplatin-treated cells, rotenone-treated cells, or controls, and which groups are being compared statistically. A clearer legend is required.
The figure legends should be revised to improve clarity.
4. Figure 8
Although Figure 8 has improved, the schematic model remains visually dense and difficult to follow. Simplification would greatly enhance accessibility for readers unfamiliar with the γ-tubulin field.

Overall Assessment

The manuscript presents several interesting observations and has improved substantially during revision. The additional quantitative analyses and biochemical experiments strengthen the study. However, the central mechanistic conclusions continue to extend beyond what is directly demonstrated by the data.
In its current form, I believe the manuscript supports a model in which TUBG is associated with centromere-containing structures and influences their spatial behaviour. The stronger claims regarding direct interaction, structural tethering, and organizational control remain insufficiently supported.
I therefore recommend further revision focused on moderating mechanistic interpretations, clarifying statistical reporting, and ensuring that conclusions remain proportional to the experimental evidence.

Reviewer #2

(Remarks to the Author)
I appreciate the authors’ efforts in carefully addressing the concerns raised during review. In particular, the addition of biochemical fractionation experiments substantially strengthens the manuscript and provides support for the microscopy-based observations. However, given that the manuscript is entitled “The Tubulin Meshwork Regulates Centromere Kinematics and Micronucleus Homeostasis,” I find the functional significance of the reported TUBG-centromere association remains somewhat unclear. If the manuscript were intended primarily to describe a structural association between the TUBG meshwork and centromeres, the current data might be sufficient. However, the use of the term “regulates” implies a functional role, and I believe the biological consequences of this association for centromere function remain insufficiently explored.

Regarding my comment that “Quantitative analysis is required to show the proportion of cells exhibiting the described phenotype,” the authors state that “TUBG was detectable within all analyzed centromere-defined volumes (n = 100 per condition) across all cell lines studied.” However, this result appears difficult to reconcile with the representative images shown in Fig. 1D, where several centromeric foci do not appear to exhibit an obvious TUBG signal. The authors should clarify how “detectable” was defined in their analysis and provide sufficient methodological detail to explain this apparent discrepancy between the quantitative measurements and the representative images.

Regarding the response to my comment on page 31, I remain somewhat uncertain about the proposed mechanism of micronucleus formation. While the authors suggest that cellular stress is responsible, it remains unclear to me whether stress-induced mitotic defects or mitotic arrest might contribute to the formation of micronuclei. If such events occur, they may represent an important intermediate step linking cellular stress to micronucleus formation. Therefore, it would be helpful for the authors to quantify and report the extent of mitotic defects and/or mitotic arrest observed under these

Version 2:

Reviewer comments:

Reviewer #1

(Remarks to the Author)
I have carefully examined the authors' rebuttal and the revised version of the manuscript.
Overall, I believe the authors have satisfactorily addressed all of the concerns raised in my previous review. In particular, they have consistently moderated the interpretation of their data, distinguishing structural association from direct molecular interaction, avoiding overinterpretation of the functional implications, and clarifying both the statistical reporting and the presentation of the figures. The revised conclusions now appear to be appropriately supported by the experimental evidence.

Based on the revised manuscript and the authors' responses, I consider that my previous concerns have been adequately addressed and I have no further comments.

Reviewer #2

(Remarks to the Author)
I appreciate the authors' efforts in addressing my previous comments and revising the manuscript accordingly. Several of my concerns have been satisfactorily addressed. However, I find the response to Comment #2 particularly problematic. In my opinion, this issue directly affects the interpretation of the central findings of the study.
Moreover, if the same definition of "detectable" has been applied throughout the manuscript, this concern may extend beyond Figure 1 and could influence the interpretation of other analyses that rely on similar quantitative measurements.

Author response #1
I appreciate the authors' efforts to incorporate my suggestions and revise the manuscript accordingly. The revised text more accurately reflects the experimental findings and avoids implying a mechanistic role that is not fully supported by the data. However, I still have a minor concern. While TUBG depletion alters centromere-associated phenotypes, I am not convinced that the observed increase in centromere fluorescence intensity necessarily indicates an alteration of centromere function. Therefore, I remain somewhat cautious about interpreting these findings as evidence of functional consequences at the centromere level.
Author response #2
In their response, the authors explain that "weak or spatially restricted TUBG signal contributing to the quantitative measurements may not be visually apparent." I consider this problematic, as such signals are then used to support the conclusion that TUBG is detectable in 100% of centromeres. The conclusion appears to depend heavily on the definition of "detectable," which, in my opinion, is currently too permissive to support such a claim.
Author response #3
I appreciate the authors' clarification and the restriction of their conclusions.

Open Access This Peer Review File is licensed under a Creative Commons Attribution 4.0 International License, which permits use, sharing, adaptation, distribution and reproduction in any medium or format, as long as you give appropriate credit to the original author(s) and the source, provide a link to the Creative Commons license, and indicate if changes were made.
In cases where reviewers are anonymous, credit should be given to 'Anonymous Referee' and the source.
The images or other third party material in this Peer Review File are included in the article's Creative Commons license, unless indicated otherwise in a credit line to the material. If material is not included in the article's Creative Commons license and your intended use is not permitted by statutory regulation or exceeds the permitted use, you will need to obtain permission directly from the copyright holder.
To view a copy of this license, visit https://creativecommons.org/licenses/by/4.0/

LUND UNIVERSITY
Faculty of Medicine

Malmö 10-04-2026

Ph.D. Maria Alvarado Kristensson
Division for Molecular Pathology
Inga Marie Nilsson street 53
Se-214 28 Malmö, Sweden
maria.alvarado-kristensson@med.lu.se

Point-by-point reply
****

Response to Reviewers

Manuscript Title: The Tubulin Meshwork regulates centromere kinematics and Micronucleus Homeostasis **Manuscript ID:** COMMSBIO-25-7197-T

We thank the Reviewers for their insightful and demanding critique of our work. Their comments have challenged us to refine our terminology and, most importantly, to transition from a descriptive study to a quantitative, functional characterization of the TUBG-centromere axis.

In response, we have performed extensive new 4D kinematic analyses and volumetric quantifications. These dynamic data provide a complementary and functionally essential layer of evidence.

REVIEWERS' COMMENTS:

Reviewer #1 (Remarks to the Author):
In this manuscript, Muteb et al. investigate the relationship between the γ-tubulin meshwork and centromeres, starting from ChIP-Seq analysis and extending into cellular and imaging studies. The study addresses a timely and interesting question, and the data suggest intriguing links between γ-tubulin, centrosomes, and centromere organization in interphase. Overall, this study presents valuable data on γ-tubulin and centromere organization. The work convincingly shows γ-tubulin remodelling under stress, centromere organization mediated by phosphorylation-dependent nucleation, and micronuclei involvement. Indeed, the dynamic localization of γ-tubulin into micronuclei and its association with DNA replication is intriguing and raises new questions. However, the manuscript in its current form contains claims that are insufficiently supported by the presented data. The claims regarding nuclear–cytosolic connections and γ-tubulin–mediated control of centromeres are not well established. Additional quantification, improved presentation, and further experiments are needed to substantiate the central hypothesis.

Main concerns that need to be addressed:

1. Nearly 40% of references are self-citations from the corresponding author, which should be reduced.

Telephone Int +46 40338391 (direct)
Postal address Lund University, Division for Molecular Pathology, SUS-Malmö, 59 Jan Waldenström street
Se-205 02 Malmö, Sweden
E-mail maria.alvarado-kristensson@med.lu.se

LUND UNIVERSITY
Faculty of Medicine

Ph.D. Maria Alvarado Kristensson
Division for Molecular Pathology
Inga Marie Nilsson street 53
Se-214 28 Malmö, Sweden
maria.alvarado-kristensson@med.lu.se

Author response #1

We have significantly revised the reference list to provide a broader context of the field. While our group has pioneered the characterization of TUBG-containing γ-strings and γ-tubules, we have now incorporated additional citations from independent groups regarding centromere kinematics, nuclear envelope proteins, phosphorylation of Ser-131 and genomic stability. This has reduced our self-citation rate to 24%, ensuring the work is situated within the wider body of literature.

Reviewer #1: 2. It is not clear throughout the text how the authors distinguish γ-tubulin meshwork from centrosomes. This distinction should be explicitly stated and consistently addressed.

Author response #2

Distinguishing TUBG meshwork from centrosomes. We have clarified this distinction throughout the revised manuscript (second paragraph of the Introduction, first paragraph of the Results, and first paragraph of Image processing and visualization within Materials and Methods). We define the "centrosome" as the canonical SAS6/TUBG-positive MTOC, characterized morphologically as a high-intensity, spherical TUBG-positive focal mass. The "TUBG meshwork" refers to the integrated protein scaffold encompassing the centrosome as well as the non-centrosomal γ-strings and γ-tubules. Furthermore, we explicitly define the "Centrosome-Nuclear Bridge" as the specific pericentriolar TUBG mass that physically breaches the nuclear envelope.

To rigorously separate these compartments for quantification, we utilized 3D-volumetric segmentation. Specifically, the "internal" or encroaching TUBG fraction was isolated by performing a Boolean AND operation between the total TUBG mask and the solid 3D nuclear interior mask (fifth and sixth paragraphs of Image processing and visualization within Materials and Methods). This mathematical distinction allowed us to precisely isolate and quantify the Centrosome-Nuclear Bridge and its interactions with centromeres independently of the main cytoplasmic centrosomal body (see Fig. 3E). This structural and mathematical differentiation was further validated by SAS6 labeling in fixed material and Pericentrin (PCNT) labeling in isolated biochemical complexes (see Figs. 3A, 5A, and 7H), confirming the spatial relationship between the canonical centrosome and the wider TUBG meshwork.

Reviewer #1: 3. I have found some overstated claims:
- The proposed mechanism that cytosolic γ-tubulin controls centromeres is not sufficiently supported by the data. Additional experiments suggested below
- The claim that centrosomes participate in centromere nucleation cannot be made based only on fixed-cell immunolabeling (Figure 5).
- Data presented on γ-tubulin–centrosome dynamics demonstrate association but not direct interaction or organizational control of centromeres.

Telephone Int +46 40338391 (direct)
Postal address Lund University, Division for Molecular Pathology, SUS-Malmö, 59 Jan Waldenström street
Se-205 02 Malmö, Sweden
E-mail maria.alvarado-kristensson@med.lu.se

LUND UNIVERSITY
Faculty of Medicine

Ph.D. Maria Alvarado Kristensson
Division for Molecular Pathology
Inga Marie Nilsson street 53
Se-214 28 Malmö, Sweden
maria.alvarado-kristensson@med.lu.se

Author response #3

We appreciate the reviewer's perspective on the need for higher structural resolution. We have significantly refined our manuscript to clarify that the TUBG meshwork serves as a functional and mechanical scaffold. To address the reviewer's concerns regarding "direct interaction" versus "stochastic association," we have highlighted several key lines of evidence:

- *Regarding "Association" vs. "Direct Interaction": To move beyond the limitations of immunofluorescence, we performed biochemical isolation of nuclear γ-tubulin complexes via discontinuous sucrose gradients (Fig. 7H). Our results show that both centrosomes and γ-tubules remain physically tethered to centromeric DNA throughout the isolation process. This co-fractionation provides biochemical evidence of a stable, physical complex that persists outside the cellular environment, supporting the presence of a direct interaction rather than transient or stochastic associations, directly addressing the concern that these are merely transient or accidental associations.*
- *Regarding the "Stochastic" vs. "Organized" nature of the interaction: We have clarified in the text that TUBG is not merely "associated" with a subset of centromeres. Our combined ChIP-seq and high-resolution immunofluorescence mapping confirms that TUBG is a consistently detected across all analyzed centromere-defined volumes (n = 100 per condition) across all studied cell lines (U2OS, H9-NSC, and ccRCC). This non-random and recurrent occupancy supports a fundamental architectural role for TUBG in centromere homeostasis.*
- *Regarding "Nucleation" vs. "Fixed-cell labeling": We acknowledge the reviewer's point that "nucleation" is a high-threshold claim for fixed-cell data. To address this, we have tempered our language throughout the manuscript, replacing "nucleation" with "structural organization" or "scaffolding." We have also emphasized that our fixed-cell data (Fig. 5) is cross-validated by our live-cell 4D tracking (Results: "TUBG localization and its role in centromere homeostasis"), which shows the persistent physical coordination of the TUBG and centromeres over time, demonstrating a functional relationship rather than a static snapshot.*
- *Regarding "Control" vs. "Association": To clarify the organizational role of TUBG, we now describe the meshwork as a "mechanical buffer" and "kinematic scaffold." This is supported by our Boolean intersection analysis (Fig. 3C) and the transition from a mobile to a "stalled" centromere state upon TUBG depletion. We propose that TUBG provides the viscoelastic support necessary for centromere mobility and stability, particularly under the genomic strain induced by cisplatin.*

Reviewer #1: 4. Data presentation:

- Table 1: Units of distance to the centromere must be specified.

Telephone Int +46 40338391 (direct)
Postal address Lund University, Division for Molecular Pathology, SUS-Malmö, 59 Jan Waldenström street
Se-205 02 Malmö, Sweden
E-mail maria.alvarado-kristensson@med.lu.se

LUND UNIVERSITY
Faculty of Medicine

Ph.D. Maria Alvarado Kristensson
Division for Molecular Pathology
Inga Marie Nilsson street 53
Se-214 28 Malmö, Sweden
maria.alvarado-kristensson@med.lu.se

- Figure 1C: Axes lack units (density and distance).
The MCF10A plot should be zoomed into the relevant density range (around 1e–07) to highlight the three reliable peaks.
- Figure 3D: Immunoelectron microscopy panels are too small to appreciate γ-tubulin labelling or chromatin. I suggest to enlarge panels. In addition, tomograms of 250 nm sections are needed to clarify direct associations.
- Movie 1: numbering centromeres would improve clarity of the dynamic interactions.

Author response #4

We thank the reviewer for these specific suggestions, which have significantly improved the clarity and technical transparency of our data presentation.

- *Table 1 (Units of Distance): We have updated Table 1 to explicitly state that all distance measurements between TUBG structures and centromeres are provided in base pairs (bp).*
- *Figure 1C (Axes and Scaling):*
  -Units: We have added the missing units to the axes (Density: arbitrary units; Distance: base pairs).
  -MCF10A Scaling: As suggested, we have adjusted the Y-axis scale for the MCF10A plot to zoom into the 1e–07 density range. This adjustment clearly highlights the three constitutive peaks, providing a more accurate representation of the signal-to-noise ratio in this cell line.
- *Figure 3D (Immunoelectron Microscopy & Section Thickness):*
  -Panel Size: We have enlarged the panels in Figure 3D to better visualize the gold-labeled -tubulin and the underlying chromatin architecture.
  -Section Thickness (250 nm) & Resolution: Regarding the suggestion for thicker (250 nm) sections for tomograms: We intentionally utilized thinner sections (typically 70–100 nm) to achieve the necessary spatial resolution for this specific protein. Due to the high endogenous density of TUBG within these cells, thicker sections lead to a significant increase in secondary antibody/gold-cluster binding. This creates a "crowding" effect that makes it mathematically difficult to distinguish specific centromeric accumulations from the high-density background of the pericentriolar matrix (PCM). By using thinner sections, we reduce the volumetric "noise" and can more confidently identify discrete, direct associations between TUBG and chromatin.
- *Movie 1 (Centromere Numbering): We have re-rendered Movie 1 to include numerical labels for individual centromeres. This allows for easier tracking of the dynamic interactions and kinematic "stalling" described in the text.*

Reviewer #1: Points Requiring Additional Analysis or Experiments:
- For ChIP-Seq data, include a clear statement on co-localization of γ-tubulin and centromeres, specifying whether a distinct pattern is observed.

Author response #5

Telephone Int +46 40338391 (direct)
Postal address Lund University, Division for Molecular Pathology, SUS-Malmö, 59 Jan Waldenström street
Se-205 02 Malmö, Sweden
E-mail maria.alvarado-kristensson@med.lu.se

LUND UNIVERSITY
Faculty of Medicine

Ph.D. Maria Alvarado Kristensson
Division for Molecular Pathology
Inga Marie Nilsson street 53
Se-214 28 Malmö, Sweden
maria.alvarado-kristensson@med.lu.se

We thank the reviewer for this important comment. To address this point, we complemented our peak-based analysis with a signal-based centromere-centered analysis of normalized ChIP-seq coverage across annotated centromeres (new Fig. 1B and 1C).
This analysis demonstrates that TUBG signal is consistently detected across centromere-centered regions and shows a reproducible distribution pattern across all analyzed cell-cycle stages. Importantly, both metaprofiles and heatmaps indicate that this pattern is stable over time and non-random, supporting a persistent association of TUBG with centromere-associated chromatin.
We note that the signal is heterogeneous across individual centromeres, as shown by the heatmaps, and does not form a uniform peak across all loci. However, the centromere-centered alignment demonstrates that TUBG occupancy is consistently enriched in regions associated with annotated centromeres, indicating a reproducible centromere-centered pattern rather than a stage-specific redistribution.
Together with our imaging-based 3D quantification, these results support the conclusion that TUBG is broadly associated with centromere-defined structures, while ChIP-seq reveals its reproducible chromatin-associated signal centered on centromeric regions.

Reviewer #1: - Quantify the percentage of centrosomes in non-differentiated neuronal cells that contact the nuclear envelope (Figure 3A), including number of cells analysed and error margins.
Author response #6
*We have updated the manuscript (Results: Centrosome-associated TUBG bridges cytosol to nuclear chromatin) to include the requested quantification. In non-differentiated H9-NSC cells, we found that 69.8% ± 16.4, (n = 3 experiments, 600 total cells analyzed) of centrosomes maintain direct contact with the nuclear envelope. This baseline association was found to be highly significant (***P < 0.001) compared to a random spatial distribution model.*

Reviewer #1: - To substantiate claims of direct interaction and structural contribution of γ-tubulin to centromere organization, correlative cryo-electron tomography is strongly recommended.
Author response #7
We appreciate the reviewer's suggestion of cryo-ET for resolving static molecular architecture. Although cryo-ET is the gold standard for static structural biology, our hypothesis centers on kinetic homeostasis—a fundamentally temporal process that cannot be captured in a vitrified state.
We agree that cryo-ET provides high-resolution structural information. However, because our study focuses on dynamic interphase behavior, we complemented structural approaches with 4D live-cell kinematics and biochemical isolation for the following reasons.

- *Functional vs. static evidence: Cryo-ET provides a "frozen" snapshot of a single moment in a highly constrained volume. Our study focuses on the active functional homeostasis and kinetic behavior of centromeres (e.g., the transition from "mobile"*

Telephone Int +46 40338391 (direct)
Postal address Lund University, Division for Molecular Pathology, SUS-Malmö, 59 Jan Waldenström street
Se-205 02 Malmö, Sweden
E-mail maria.alvarado-kristensson@med.lu.se

LUND UNIVERSITY
Faculty of Medicine

Ph.D. Maria Alvarado Kristensson
Division for Molecular Pathology
Inga Marie Nilsson street 53
Se-214 28 Malmö, Sweden
maria.alvarado-kristensson@med.lu.se

to "stalled" states upon TUBG depletion). This requires the temporal resolution provided by 4D tracking.

- *Sampling and volumetric complexity: The TUBG meshwork is an expansive, cell-wide structure bridging the cytosol and the deep nuclear interior. The small sampling volume of cryo-ET makes it technically challenging to capture long-range structures such as centrosome–centromere connections.*
- *Biochemical validation of direct interaction: To address the reviewer's request for evidence of direct interaction without the sampling limitations of cryo-ET, we performed biochemical isolation of nuclear TUBG complexes (Fig. 7H). The co-fractionation of TUBG and centromeric DNA demonstrates a stable physical association consistent with direct interaction, which complements our high-resolution Airyscan 3D-volumetric reconstructions.*
- *Optical Tomography as an Alternative: Our Airyscan 2 datasets provide "optical tomograms" combining 3D structure with molecular identity, enabling quantitative analysis across large cell populations.*

Reviewer #1: - The assertion that γ-tubulin tubules connect the nuclear compartment to microtubules requires higher-resolution imaging (again, correlative cryo-tomography would be appropriate).

Author response #8

To substantiate the physical connection between γ-tubules and the microtubule network without the sampling limitations of cryo-ET, we have provided high-resolution structural validation in our revised Figure 8D. We have successfully isolated the γ-tubulin/microtubule complex and performed immunofluorescence on the purified fractions.

By utilizing Colocalization Pixel Mapping (CPM) on these isolated structures, we demonstrate distinct, non-random interaction points between γ-tubules and TUBG (yellow arrowheads). This biochemical evidence, coupled with our confocal imaging of whole cells, provides a robust confirmation of the structural linkage between the nuclear compartment and the wider microtubule network.

Reviewer #2 (Remarks to the Author):

Reviewer #2: This manuscript addresses an ambitious and thought provoking hypothesis that the TUBG meshwork not only bridges the cytoplasm and the nucleus but also provides a structural scaffold for the establishment of functional centromeres. While the premise is intriguing, I find the current evidence insufficient in both depth and rigor to substantiate such a far reaching claim. A major concern is that many of the key findings rely primarily on microscopy based observations. To ensure robustness and reproducibility, comprehensive quantitative analyses and appropriate statistical validation should be included throughout. In addition, the central assertion, that the cytoplasmic TUBG network penetrates the nuclear envelope and functions within the nuclear compartment, cannot be convincingly supported by imaging alone. Biochemical approaches would be essential to strengthen this conclusion. For these

Telephone Int +46 40338391 (direct)
Postal address Lund University, Division for Molecular Pathology, SUS-Malmö, 59 Jan Waldenström street
Se-205 02 Malmö, Sweden
E-mail maria.alvarado-kristensson@med.lu.se

LUND UNIVERSITY
Faculty of Medicine

Ph.D. Maria Alvarado Kristensson
Division for Molecular Pathology
Inga Marie Nilsson street 53
Se-214 28 Malmö, Sweden
maria.alvarado-kristensson@med.lu.se

reasons, which are detailed further in my specific comments below, I do not recommend acceptance of the manuscript in its present form.

Author response #1

We agree that imaging alone is insufficient to establish functional interactions; therefore, we complemented imaging with quantitative 3D analysis and biochemical co-fractionation (Fig. 7H), which together support a stable association consistent with direct interaction.

<u>Figures 1D and 1E</u>

Quantitative analysis is required to show the proportion of cells exhibiting the described phenotype. Statistical evaluation should subsequently be performed to support the validity of these observations.

Author response #2

We agree with the reviewer that establishing the robustness of the TUBG-centromere interaction across the cell population is essential. To move beyond representative microscopy and address the potential for stochastic overlap, we performed systematic automated 3D-segmentation and volumetric quantification of TUBG fluorescence intensity within the total centromere population.

- *Our analysis confirmed that TUBG was detectable within all analyzed centromere-defined volumes ($n = 100$ per condition) across all cell lines studied (U2OS, H9-NSC, and ccRCC). This consistent detection across experiments was observed consistently across all independent experiments, under both homeostatic conditions and various stress states (rotenone and cisplatin).*
- *To provide a rigorous statistical baseline, we compared TUBG enrichment to Lamin B, a nuclear envelope protein that does not constitutively associate with the centromere interior. As shown in Figure 1F, TUBG mean fluorescence intensity (MFI) was significantly higher than the Lamin B background ($P < 0.0001$, Wilcoxon matched-pairs signed-rank test), confirming a specific structural enrichment rather than a random nuclear distribution.*
- *To address the concern that these findings rely on "imaging alone," we cross-validated these quantitative spatial observations with biochemical isolation of stable TUBG-centromere complexes (Fig. 7H). The fact that these complexes survive biochemical fractionation via sucrose gradients confirms that the imaging-based observations reflect a stable physical association consistent with direct interaction.*

Collectively, these data demonstrate that the TUBG-centromere interaction is a fundamental, invariant structural feature of the nuclear architecture in the models studied, rather than an occasional or stochastic phenotype.

Reviewer #2:

<u>Figure 2A</u>

Quantitative analysis is also necessary to substantiate the described results.

Author response #3

Telephone Int +46 40338391 (direct)
Postal address Lund University, Division for Molecular Pathology, SUS-Malmö, 59 Jan Waldenström street
Se-205 02 Malmö, Sweden
E-mail maria.alvarado-kristensson@med.lu.se

LUND UNIVERSITY
Faculty of Medicine

Ph.D. Maria Alvarado Kristensson
Division for Molecular Pathology
Inga Marie Nilsson street 53
Se-214 28 Malmö, Sweden
maria.alvarado-kristensson@med.lu.se

We agree and have ensured that all visual observations in Figure 2A are backed by systematic quantification.

- *We performed automated 3D-segmentation and volumetric quantification of the TUBG signal at centromeres in both normal and ccRCC cells. As detailed in the Results, TUBG signal was detected within all analyzed centromere-defined volumes (Normal:* 25.15 ± 8.66 *A.U.; ccRCC:* 15.08 ± 5.02 *A.U.).*
- *We quantified the spatial proximity of these complexes to the centrosome, finding that 24 ± 7% (Normal) and 15 ± 4% (ccRCC) of centromere-TUBG hubs (n = 400) were clustered near the MTOC. This provides the quantitative rigor requested to support the "structural link" hypothesis.*

Reviewer #2:
Figure 2B The number of cells counted and whether their expression levels were comparable should be clearly indicated to ensure data reliability. The authors show that centromere signal intensity increases in cells with reduced TUBG expression compared with Cas9-GFP-TUBG1-sg non-expressing cells (Fig. 1C or p16). However, this observation alone does not establish that TUBG functions as a stabilizing factor required to maintain proper centromere organization, nor that TUBG prevents excessive condensation or aberrant centromere assembly during interphase. It also remains unclear whether the increased centromere signal represents an abnormal structural state or whether it is associated with any functional defects, such as impaired chromosome segregation or mitotic progression.

Author response #4

We appreciate this opportunity to clarify the mechanistic link between TUBG levels and centromere homeostasis.

- *We have updated the Figure 2B legend to explicitly state that n = 100 cells were analyzed for the sg-expressing and non-expressing internal control populations. To ensure data reliability, we did not rely solely on GFP expression as a proxy for knockdown. Instead, we quantitatively verified the TUBG protein concentration in every GFP-positive cell; only those exhibiting a confirmed reduction (averaging 35.66%) were included. This ensures that the observed centromere compaction is strictly correlated with reduced TUBG levels rather than a generic response to the Cas9-GFP vector.*
- *We contend that the increased centromere intensity is a direct indicator of stress-induced chromatin compaction resulting from a loss of TUBG-mediated stabilization. Our 4D data show that TUBG-depleted cells exist in a "stalled" state with a nearly 75% reduction in mobility (0.36 ± 0.92 µm/min vs. 1.31 ± 0.78 µm/min in controls).*
- *This loss of mobility prevents the centromeres from navigating the nuclear volume, leading to an interphase kinetic collapse. In this state, centromeres become excessively condensed. Crucially, we show that chemical stress (cisplatin) and genetic stress (sgTUBG1) converge on this same mechanism: both induce compaction by restricting*

Telephone Int +46 40338391 (direct)
Postal address Lund University, Division for Molecular Pathology, SUS-Malmö, 59 Jan Waldenström street
Se-205 02 Malmö, Sweden
E-mail maria.alvarado-kristensson@med.lu.se

LUND UNIVERSITY
Faculty of Medicine

Ph.D. Maria Alvarado Kristensson
Division for Molecular Pathology
Inga Marie Nilsson street 53
Se-214 28 Malmö, Sweden
maria.alvarado-kristensson@med.lu.se

fluid dynamics. In sg-cells, the pre-existing "stalled" state limits further cisplatin-induced clustering, which explains why their relative intensity increase is lower than in mobile control cells.

- *To address the reviewer's point on functional defects, we have added text referencing that this interphase compaction is associated with micronucleus formation and nuclear envelope instability (later figures), which are hallmarks of impaired genomic maintenance and eventual mitotic failure.*

Reviewer #2:
Figures 3A-C: All panels require quantitative analysis to substantiate the presented findings.
Author response #5
We agree that visual evidence of nuclear encroachment must be supported by rigorous quantification. We have now integrated extensive 3D-volumetric and population-level analysis into Figure 3:

- *Population statistics (Fig. 3A): We quantified the frequency of centrosome-NE contact across $n = 600$ cells. We found that $69.8 \pm 16.4\%$ of centrosomes in H9-NSCs maintain direct physical contact with the nuclear envelope ($P< 0.001$), establishing this as a dominant structural state in these cells.*
- *Volumetric Encroachment (Fig. 3C): Using Boolean intersection analysis of high-resolution Airyscan 2 datasets, we quantified that 62.25% of the total centrosomal TUBG volume (4.003 μm^3) resides within the boundaries of the nuclear envelope.*
- *Chromatin Anchoring Index: We identified a specific internal volume of 0.183 μm^3 in direct physical contact with DNA. We introduced a DNA interaction index (0.046) to provide a numerical basis for the "cytosolic-nuclear bridge," demonstrating a stable terminal anchor at the host genome.*

Reviewer #2:
Supplementary Figure 1A and Movie 1
The authors describe an observation in which the centrosome moves from the imaging plane toward the nuclear envelope (labeled withmCherry-NUP50 and Hoechst) and reappears within a few minutes. This behavior should be quantitatively analyzed. It also remains unclear whether this represents a biologically meaningful event rather than a coincidental or technically induced observation.
Author response #6
We agree that distinguishing active biological movement from technical jitter or stochastic "drifting" is essential. We have substantiated the biological significance of these centrosome-nuclear dynamics through two complementary approaches:

- *Kinematic signature analysis (stable vs. active states): Our detailed analysis of $n = 1050$ frames (16 centrosomes) reveals a distinct "stop-and-go" behavior. The centrosome maintains a "stable state" (displacement < 0.1 μm) for $46.86 \pm 11.98\%$ of*

Telephone Int +46 40338391 (direct)
Postal address Lund University, Division for Molecular Pathology, SUS-Malmö, 59 Jan Waldenström street
Se-205 02 Malmö, Sweden
E-mail maria.alvarado-kristensson@med.lu.se

LUND UNIVERSITY
Faculty of Medicine

Ph.D. Maria Alvarado Kristensson
Division for Molecular Pathology
Inga Marie Nilsson street 53
Se-214 28 Malmö, Sweden
maria.alvarado-kristensson@med.lu.se

the observation period. This intermittency is characteristic of tethered or motor-driven biological movement. In contrast, technical drift or Brownian motion would typically present as a continuous, erratic, or linear displacement without these high-frequency "docking" intervals.

- *Threshold of spatial significance: When active repositioning occurs, we measured a mean step of 0.1236 ± 0.0712 µm and a total cumulative distance of 4.42 ± 1.69 µm per session. This cumulative exploration of the nuclear periphery far exceeds the measured limits of technical drift in our Airyscan system, which utilized active stabilization for long-term live imaging.*
- *Biological Context (TUBG Accumulation): The validity of the centrosome as a functional platform is further supported by our observation that pharmacological stressors (Cisplatin/Rotenone) lead to the specific accumulation of TUBG at these centrosomal sites. The fact that the cell selectively recruits structural proteins to the centrosome under stress reinforces that the centrosome—and its proximity to the nucleus—is a biologically regulated focal point rather than a coincidental observation.*

The combination of intermittent kinematics (stable states) and stress-responsive protein recruitment (TUBG accumulation) strongly indicates that the centrosome-nuclear interface is a functional, biologically meaningful apparatus.

Reviewer #2:
Supplementary Figure 1B
Quantitative analysis is required to support the validity of the observations.

Author response #7

We agree that quantification is essential to distinguish biological docking from stochastic proximity. We have now provided three layers of quantitative validation for the observations in Supplementary Figure 2B:

- *Volumetric Occupancy: We performed 3D-volumetric colocalization on the frames capturing these transient contacts. The thresholded Mander's Coefficient (M2) reached 1.0, with a mean colocalized volume of 90.03% ± 7.42% between the TUBG-GFP and Lamin B-mCherry channels, confirming high-occupancy structural docking rather than coincidental overlap.*
- *Kinetic Stability: 4D-tracking (n = 1050 frames) revealed that these contacts are part of a "stable state" (displacement < 0.1 µm) maintained for 46.86% ± 11.98% of the observation period, indicating a tethered residency rather than rapid transit.*
- *Line Profile Analysis: As shown in the updated Supplementary Fig 2B, spatially coincident fluorescence intensity peaks for TUBG and Lamin B confirm physical occupancy within the same 0.1-µm optical slice.*

Reviewer #2:

Telephone Int +46 40338391 (direct)
Postal address Lund University, Division for Molecular Pathology, SUS-Malmö, 59 Jan Waldenström street
Se-205 02 Malmö, Sweden
E-mail maria.alvarado-kristensson@med.lu.se

LUND UNIVERSITY
Faculty of Medicine

Ph.D. Maria Alvarado Kristensson
Division for Molecular Pathology
Inga Marie Nilsson street 53
Se-214 28 Malmö, Sweden
maria.alvarado-kristensson@med.lu.se

Page 22
The statement, "interacting with centromeres, which resulted in distinct scenarios of centrosome-centromere interactions," is not substantiated by the presented data. No direct evidence of centrosome-centromere interaction is provided, and this claim should therefore be revised or removed.

Author response #8

We agree that our previous version relied heavily on proximity in whole-cell imaging. To provide the "direct evidence" requested, we have performed extensive new experiments, now included as Panels 7H and 8D, which provide biochemical and structural proof of these interactions:

- *New biochemical evidence (Fig. 7H): We have now included a biochemical isolation of TUBG-rich complexes from nuclear extracts using sucrose gradient fractionation. The Western blot results clearly show that CENP-A co-fractionates with TUBG. This provides biochemical evidence of a stable association consistent with direct interaction.*
- *New imaging analysis of isolated complexes (Fig. 7H & 8D): We performed confocal microscopy on these purified fractions. These new panels show isolated γ-tubules and centrosomes physically tethered to centromeric DNA and α-tubulin lattices outside of the cellular context. This confirms that the "interaction" is a stable structural docking capable of surviving the biochemical isolation process.*
- *Refined 3D Quantification (Fig. 7F): We have added a new 3D-volumetric analysis that confirms a statistically significant ($P< 0.0001$) enrichment of TUBG within the actual volumes of centromeres under stress.*

We believe these new data, particularly the biochemical co-fractionation in Fig. 7H, provide the direct substantiation required to support our claims of a functional centrosome-centromere interaction.

Reviewer #2:
The authors also state, "centrosomes interacted with a single centromeric focus, which subsequently underwent division into two independent centromeric foci." This centrosome-centromere interaction should be quantitatively analyzed to indicate the percentage of centromeres exhibiting this behavior. It should also be clarified whether the same centromeres consistently undergo this process or if it occurs randomly. A comparison between centromeres that interact with centrosomes and those that do not would help determine whether meaningful differences exist. From the presented data, the majority of centromeres do not appear to engage in such interactions, raising questions about the biological significance of this observation.
The authors further propose that "this dynamic interplay highlights a potential role for the TUBG-associated centrosome in actively facilitating the reorganization or maturation of centromeres during interphase." However, it is not evident from the data that this conclusion is supported. As a first step, the authors should provide quantitative information on the proportion of cells exhibiting this phenomenon to establish its reproducibility and relevance.

Author response #9

Telephone Int +46 40338391 (direct)
Postal address Lund University, Division for Molecular Pathology, SUS-Malmö, 59 Jan Waldenström street
Se-205 02 Malmö, Sweden
E-mail maria.alvarado-kristensson@med.lu.se

LUND UNIVERSITY
Faculty of Medicine

Ph.D. Maria Alvarado Kristensson
Division for Molecular Pathology
Inga Marie Nilsson street 53
Se-214 28 Malmö, Sweden
maria.alvarado-kristensson@med.lu.se

We agree that establishing the biological significance of these relatively rare, transient interactions is crucial. Rather than relying on stochastic observations in wild-type cells, we have provided a rigorous genetic and mechanistic comparison to determine if these interactions are functionally regulated (Fig. 6 and Fig. 7):

- *Proportion and Reproducibility: We quantified the frequency of these interactions across hundreds of cells (n = 474 total across conditions). While baseline interactions in control cells are transient, the phospho-mimetic mutant (Ser131Asp) significantly increases the frequency of centromere-docking foci to 26% of cells (n = 126), whereas the non-phosphorylatable mutant (Ser131Ala) almost entirely abolishes this capability (2.7%, n = 148). This binary difference demonstrates that docking is a regulated biological process, not a random collision.*
- *The "Scaffold vs. Cargo" Model: We propose that the centrosome and γ-tubule meshwork function primarily as a structural scaffold rather than a kinetic driver. Our data suggest that while the physical docking of this scaffold is a necessary baseline event, the functional regulation of the centromere is determined by the local dosage (accumulation) of TUBG recruited to these sites.*
- *Biological Significance of the "Sub-population" Response: The reviewer is correct that the majority of centromeres do not engage in these interactions simultaneously. However, our data—specifically the significant enrichment of TUBG within centromere volumes (P< 0.0001, Fig. 7F) and the biochemical co-fractionation of CENP-A with TUBG (Fig. 7H)—indicate that the biological significance lies in the targeted delivery of TUBG cargo. Much like DNA repair foci, the importance of this mechanism is found in the specificity of the recruitment to the TUBG scaffold when the "molecular switch" (Ser131 phosphorylation) is flipped.*

We have revised the text on Page 22 (Results: "Dynamic TUBG interactions orchestrate interphase centromere organization", second paragraph) to emphasize that the centrosome acts as a spatial coordinator for TUBG delivery, and that the observed effects are driven by regulated TUBG accumulation within a specific sub-population of centromeres.

Reviewer #2:
Page 23
The statement, "g-tubules formed physical connections with multiple centromeres," is not convincingly demonstrated. The positional proximity of TUBG and centromeres observed microscopically does not necessarily indicate a direct interaction. Additional analyses, ideally using biochemical approaches, are needed to confirm that this spatial relationship reflects a functional connection. Moreover, it is unclear whether all centromeres are connected in this manner or how many such connections are statistically significant. The subsequent claim that "the dynamic TUBG meshwork, including both centrosome-associated pools and nucleated g-tubules, serves as an active scaffold for interphase centromere organization" is not sufficiently supported by the current data.
Author response #10

Telephone Int +46 40338391 (direct)
Postal address Lund University, Division for Molecular Pathology, SUS-Malmö, 59 Jan Waldenström street
Se-205 02 Malmö, Sweden
E-mail maria.alvarado-kristensson@med.lu.se

LUND UNIVERSITY
Faculty of Medicine

Ph.D. Maria Alvarado Kristensson
Division for Molecular Pathology
Inga Marie Nilsson street 53
Se-214 28 Malmö, Sweden
maria.alvarado-kristensson@med.lu.se

We agree that establishing a "functional connection" requires evidence beyond positional proximity. While our previous version demonstrated that these interactions are biologically regulated via Ser131 phosphorylation (Fig. 6), we have now performed the additional biochemical analyses requested to confirm a stable physical tethering:

- *New biochemical co-fractionation (Fig. 7H): We isolated TUBG-rich complexes from nuclear extracts using discontinuous sucrose gradients. Western blot analysis of purified fractions confirms that CENP-A co-fractionates with TUBG. This provides biochemical evidence supporting a stable association consistent with direct interaction between the TUBG meshwork and centromeres.*
- *Confocal imaging of isolated complexes (Fig. 7H): We further analyzed these purified fractions via microscopy, which revealed centromeric DNA and CENP-A physically attached to isolated γ-tubules outside of the cellular context. This confirms that the observed "proximity" in cells represents a robust structural docking.*
- *Targeted vs. Global Interaction: Regarding the reviewer's question on whether all centromeres are connected, we have clarified in the text that this is a targeted sub-population response. Our 3D-volumetric analysis (Fig. 7F) shows a highly significant enrichment ($P < 0.0001$) of TUBG at a specific subset of centromeres. Much like DNA repair hubs, these structures do not need to be present at every centromere to be biologically significant; rather, they represent a localized response to cellular stress.*

We have updated the manuscript to describe the TUBG meshwork as a "regulated structural scaffold" and have anchored our conclusions to this new biochemical validation.

Reviewer #2:
Page 26
The statement, "During this transition, centrosomes localized to chromatin regions where centromeres assembled," requires statistical validation. Furthermore, Figure 5A is not well organized, which complicates data interpretation. The claim, "Instead, centromere nucleation appeared to occur at the centrosomes, suggesting that centrosomes, through their association with the TUBG meshwork, act as docking sites for centromeres," lacks clear supporting evidence in the presented data.

Author response #11

We appreciate the reviewer's feedback and have addressed these points through figure reorganization, refined terminology, and new proximity quantification.

- *Figure Reorganization: We have reorganized the panels in Figure 5A to improve clarity.*
- *Proximity Analysis: To validate the localization of centrosomes to chromatin, we performed a proximity analysis in the H9-NSC model. While the total fluorescence intensity of TUBG did not significantly increase (Fig. 5A, graph), our analysis confirms a profound spatial reorganization of the pre-existing TUBG meshwork. In stress-*

Telephone Int +46 40338391 (direct)
Postal address Lund University, Division for Molecular Pathology, SUS-Malmö, 59 Jan Waldenström street
Se-205 02 Malmö, Sweden
E-mail maria.alvarado-kristensson@med.lu.se

LUND UNIVERSITY
Faculty of Medicine

Ph.D. Maria Alvarado Kristensson
Division for Molecular Pathology
Inga Marie Nilsson street 53
Se-214 28 Malmö, Sweden
maria.alvarado-kristensson@med.lu.se

induced rounded neurons, the meshwork consistently redistributes to establish direct contact with centromeric regions.

- *Refined Terminology: We have replaced the term "nucleation" with "docking" or "spatial association." This more accurately reflects our observation that the centrosome and its associated TUBG meshwork serve as a structural anchor for existing centromeres during the transition to a rounded morphology.*
- *Supporting Evidence from U2OS: We emphasize that this docking mechanism is further substantiated by our U2OS model (Fig. 5B–E), where cisplatin and rotenone induced a statistically significant ($P < 0.0001$) enrichment of TUBG at centromere volumes, providing the quantifiable evidence for this stress-responsive coupling.*

Reviewer #2:

Figs. 5A-B also require quantitative analysis to demonstrate statistical significance. The authors should clarify how this differs from random or coincidental events.

The statement, "centromeres were arranged in ring-like structures around a single g-tubule and at intersections where two g-tubules crossed," raises additional questions: what is the biological significance of these intersections, and what specific structural or biochemical properties characterize them?

Author response #12

We have addressed these concerns by providing new statistical validations and clarifying the structural nature of the TUBG-centromere interface:

- *Statistical validation against random events: To demonstrate that the observed proximity is not coincidental, we performed 3D-volumetric quantification (Fig. 5E and 7F) across three different cell models (H9-NSCs, U2OS, and ccRCC). In U2OS and ccRCC cells, stress induced a statistically significant enrichment of TUBG fluorescence within centromere volumes ($P < 0.0001$). Furthermore, our biochemical isolation (Fig. 7H) confirms that these components remain physically tethered even after sucrose gradient fractionation. If these were random spatial overlaps, they would not persist as a stable complex during biochemical purification. The persistence of these complexes supports a stable association consistent with direct interaction.*
- *Biological significance of intersections and rings: We propose that these ring-like structures and intersections (Fig. 5C-D) serve as mechanical "docking hubs" that stabilize centromeric chromatin under stress. In our model, these intersections act as high-affinity nodes where the TUBG meshwork is densest, providing a structural anchor that prevents centromere dispersion.*
- *Structural and biochemical properties: Biochemically, these sites are characterized by the localized recruitment of TUBG and CENP-A (Fig. 7H) and are regulated by Ser131 phosphorylation (Fig. 6). Structurally, these intersections represent the convergence of γ-tubules, creating a stable lattice that supports nuclear architecture when canonical structures are compromised by genotoxic or oxidative strain.*

Telephone Int +46 40338391 (direct)
Postal address Lund University, Division for Molecular Pathology, SUS-Malmö, 59 Jan Waldenström street
Se-205 02 Malmö, Sweden
E-mail maria.alvarado-kristensson@med.lu.se

LUND UNIVERSITY
Faculty of Medicine

Ph.D. Maria Alvarado Kristensson
Division for Molecular Pathology
Inga Marie Nilsson street 53
Se-214 28 Malmö, Sweden
maria.alvarado-kristensson@med.lu.se

Reviewer #2:
Figures 6A-C Statistical analyses are required to support the presented findings.
Author response #13
We agree that statistical validation is essential for substantiating the role of Ser131 phosphorylation in centromere docking. We have now updated Figure 6 to include a quantitative analysis (panel 6D) of the phenotypic differences between the wild-type, non-phosphorylatable (Ser131Ala), and phospho-mimetic (Ser131Asp) mutants:

- *Quantitative validation of interaction: Our analysis of 678 cells across 4 independent experiments demonstrates a significant difference in the capacity to form transient TUBG-foci and recruit centromeres. Compared to wild-type controls (n= 254), the Ser131Ala mutant (n= 255) showed a significant decrease in docking competence, whereas the Ser131Asp mutant (n= 169) showed a significant increase in both assembly and recruitment.*
- *Statistical Significance: Statistical significance was determined using an unpaired Mann-Whitney test, confirming that the functional differences between the mutants are statistically significant ($P < 0.05$).*
- *Binary functional outcome: As shown in Figure 6D, we separated the data into two metrics: the ability to assemble a focus and the efficiency of those foci in recruiting centromeres. The Ser131Ala mutant showed a near-total inability to recruit centromeres (failing in 97.3% of observed cells), providing robust support for the conclusion that Ser131 phosphorylation is a functional requirement for the meshwork to act as a nuclear organizer.*
- *Metric Selection and γ-tubule visualization: We focused our quantification on the formation of TUBG "foci" rather than individual γ-tubules. As established in the literature ("Characterization of gamma-tubulin filaments in mammalian cells" DOI:10-1016/j.bbamcr.2017.10.008), γ-tubules nucleate directly from TUBG-rich centers. In our models, specifically under the spatial constraints of the nuclear periphery, it is often difficult to distinguish between a bright TUBG focus and a cluster of short γ-tubules. Therefore, quantifying the "TUBG focus" as a single functional unit provides a more robust and reproducible statistical measure of meshwork assembly.*

We have updated the Figure 6 legend and the Results section (section: "Ser 131 phosphorylation of TUBG drives centromere recruitment and dynamics"*) to include these n-values, P-values, and the specific statistical tests performed.*

Reviewer #2:
Page 31

Telephone Int +46 40338391 (direct)
Postal address Lund University, Division for Molecular Pathology, SUS-Malmö, 59 Jan Waldenström street
Se-205 02 Malmö, Sweden
E-mail maria.alvarado-kristensson@med.lu.se

LUND UNIVERSITY
Faculty of Medicine

Ph.D. Maria Alvarado Kristensson
Division for Molecular Pathology
Inga Marie Nilsson street 53
Se-214 28 Malmö, Sweden
maria.alvarado-kristensson@med.lu.se

The statements are difficult to interpret without an explanation of how these micronuclei are formed. The authors should clarify whether they arise from mitotic defects, cellular senescence, or other processes. Without this context, the biological significance of the observation cannot be evaluated.

Author response #14

We agree that defining the biological origin of the micronuclei is essential for evaluating their functional significance. We have updated the manuscript on previous Page 31 (section: "TUBG meshwork remodeling and centromere organization under cellular stress") to clarify that these structures arise from different pathological processes depending on the cell model:

- *In ccRCC and U2OS cells: The micronuclei result from mitotic catastrophe induced by the clastogenic (DNA-damaging) and aneugenic (spindle-disrupting) effects of cisplatin and rotenone. This leads to lagging chromosomes or chromatin fragments that fail to incorporate into the daughter nuclei.*
- *In differentiated H9-NSC neurons: We have clarified that these structures arise from pathological ectopic cell cycle re-entry. While these neurons are post-mitotic, severe stress triggers a well-documented but "aborted" attempt to re-engage mitotic machinery, leading to nuclear fragmentation rather than division (now cited: Herrup and Yang, 2007).*

By providing this context, we highlight that the recruitment of the TUBG meshwork is an active, regulated response to sequester damaged genomic material—a process that mirrors the centrosomal sequestration of toxic proteins observed in aggresome formation (McNaught, 2002). This suggests that the TUBG network serves as a conserved structural scaffold that manages cellular "waste" or damaged components during the transition toward cell death.

Reviewer #2:

Figure 7 is confusing and does not clearly convey the intended message.

The statement, "Furthermore, an increased number of cells with g-tubules were observed upon treatment with either cisplatin or rotenone, indicating a stress-induced alteration in how genetic material is packaged and handled within these structures," attributes the observed effects solely to g-tubules. It would be more appropriate for the authors to discuss alternative explanations or contributing factors. The statement, "The observed dynamic organization of centromeres by g-tubules in both nuclear and cytoplasmic compartments under rotenone and cisplatin treatment highlights a crucial, conserved role for the TUBG network in maintaining genome integrity and responding to various cellular insults," appears overstated. The data presented do not convincingly demonstrate that the TUBG meshwork is actively regulated in response to cellular stress.

Author response #15

We appreciate the reviewer's critical assessment of our interpretation of Figure 7. We have implemented several changes to the manuscript (section: "TUBG meshwork remodeling and centromere organization under cellular stress") to provide a more balanced and evidence-based discussion:

Telephone Int +46 40338391 (direct)
Postal address Lund University, Division for Molecular Pathology, SUS-Malmö, 59 Jan Waldenström street
Se-205 02 Malmö, Sweden
E-mail maria.alvarado-kristensson@med.lu.se

LUND UNIVERSITY
Faculty of Medicine

Ph.D. Maria Alvarado Kristensson
Division for Molecular Pathology
Inga Marie Nilsson street 53
Se-214 28 Malmö, Sweden
maria.alvarado-kristensson@med.lu.se

- *Broadening the scope of contributing factors: We agree that the reorganization of the TUBG meshwork does not occur in isolation. We have revised the text to explicitly state that this structural response likely occurs in coordination with other essential cellular processes, such as chromatin remodeling and canonical DNA damage repair signaling. This acknowledges that γ-tubules are part of a multi-factorial response rather than the sole driver of genomic packaging.*
- *Refining functional claims: We have moderated the tone of our conclusions. The phrase "maintaining genome integrity" has been replaced with a more precise description of the TUBG meshwork as a "structural facilitator" for the compartmentalization and sequestration of damaged genomic material. We believe this more accurately reflects the data presented.*
- *Evidence for Active Regulation: To address the concern that the response might be a passive byproduct of stress, we have highlighted two key pieces of evidence:*
  1. *Biochemical Stability (Fig. 7H): The fact that the γ-tubule/centromere complex remains physically tethered even after stringent biochemical isolation via sucrose gradients strongly suggests a regulated, high-affinity interaction rather than a coincidental spatial overlap caused by nuclear fragmentation.*
  2. *Structural Specificity: The increase in TUBG enrichment specifically within centromere volumes (Fig. 7F) indicates a targeted recruitment mechanism. (Note: The molecular basis for this regulation, involving Ser131 phosphorylation, is detailed in the subsequent section and Figure 8).*
- *Improving Visual Clarity: To ensure Figure 7 clearly conveys the intended message, we have updated the figure legend and provided clearer annotations (e.g., insets and arrowheads) to help the reader distinguish between baseline structures and stress-induced hubs.*

Reviewer #2:
Page 34
Finally, the statement, "Given that cellular stress and chromosome mis-segregation can lead to the formation of micronuclei," leaves unresolved whether micronuclei formation results from abnormalities in interphase TUBG-meshwork-mediated centromere organization or simply from previously reported mitotic centromere-centrosome defects. This distinction should be clearly addressed.

Author response #16

We thank the reviewer for this insightful distinction and have clarified our position on Pages 31 and 34 (section "TUBG meshwork remodeling and centromere organization under cellular stress" and eighth paragraph in the Discussion). While we acknowledge that classic mitotic defects are a well-documented source of micronuclei, our study focuses on the active interphase management of these structures once they exist.

Telephone Int +46 40338391 (direct)
Postal address Lund University, Division for Molecular Pathology, SUS-Malmö, 59 Jan Waldenström street
Se-205 02 Malmö, Sweden
E-mail maria.alvarado-kristensson@med.lu.se

LUND UNIVERSITY
Faculty of Medicine

Ph.D. Maria Alvarado Kristensson
Division for Molecular Pathology
Inga Marie Nilsson street 53
Se-214 28 Malmö, Sweden
maria.alvarado-kristensson@med.lu.se

We show that the TUBG meshwork serves as a conserved, interphase-specific scaffold that facilitates the organization and movement of centromeres, independently of the micronucleus's initial origin. By demonstrating that this meshwork is regulated by Ser131 phosphorylation even within the sequestered environment of the micronucleus, we provide evidence for a dedicated biological system that manages chromosomal material throughout the cell cycle—a role that is functionally distinct from the centrosome-mediated mitotic spindle.

Telephone Int +46 40338391 (direct)
Postal address Lund University, Division for Molecular Pathology, SUS-Malmö, 59 Jan Waldenström street
Se-205 02 Malmö, Sweden
E-mail maria.alvarado-kristensson@med.lu.se

LUND UNIVERSITY
Faculty of Medicine

Malmö 14 June 2026

Ph.D. Maria Alvarado Kristensson
Division for Molecular Pathology
Inga Marie Nilsson street 53
Se-214 28 Malmö, Sweden
maria.alvarado-kristensson@med.lu.se

Point-by-point reply
****

Response to Reviewers

Manuscript Title: The TUBG meshwork is associated with centromere dynamics and micronucleus-associated organization **Manuscript ID:** COMMSBIO-25-7197A-Z

We thank the Reviewers for their insightful and constructive critiques of our work. Their comments challenged us to refine our terminology, clarify the distinction between association and direct interaction, improve the presentation and interpretation of our data, and ensure that our conclusions remain proportional to the experimental evidence.

In response, we have extensively revised the manuscript, including clarification of the imaging and biochemical methodologies, revision of figure presentation and statistical reporting, replacement of inappropriate citations with primary literature where applicable, and moderation of mechanistic interpretations throughout the text. Together, these revisions provide a more rigorous and balanced presentation of the association between TUBG-containing structures, centromere-defined regions, and micronucleus-associated chromatin.

REVIEWERS' COMMENTS:

Reviewer #1 (Remarks to the Author):
The authors have substantially revised the manuscript and have clearly invested considerable effort in addressing many of the concerns raised during the previous review round. The manuscript is now better organized, the distinction between centrosomal and non-centrosomal TUBG structures is more clearly described, additional quantitative analyses have been incorporated, and several overstated claims have been moderated. The inclusion of additional quantitative measurements of centrosome–nuclear envelope association, and biochemical fractionation experiments has improved the overall quality of the work.

The study addresses an interesting and potentially important question regarding the role of the γ-tubulin meshwork in centromere organization and nuclear architecture. However, several conceptual and methodological concerns remain unresolved. In particular, the evidence presented continues to support association and correlation more strongly than direct interaction or mechanistic control. While I believe the manuscript has improved significantly, additional revisions are required before the conclusions can be fully supported by the available data.

Main concerns that need to be addressed:

Telephone Int +46 40338391 (direct)
Postal address Lund University, Division for Molecular Pathology, SUS-Malmö, 59 Jan Waldenström street
Se-205 02 Malmö, Sweden
E-mail maria.alvarado-kristensson@med.lu.se

LUND UNIVERSITY
Faculty of Medicine

Ph.D. Maria Alvarado Kristensson
Division for Molecular Pathology
Inga Marie Nilsson street 53
Se-214 28 Malmö, Sweden
maria.alvarado-kristensson@med.lu.se

1. Evidence for Direct Interaction Remains Indirect
   One of my principal concerns from the previous review was the distinction between spatial association and direct interaction.

   The authors now provide biochemical co-fractionation data showing that TUBG-containing structures and centromeric DNA remain associated during fractionation. While this is an interesting observation, co-fractionation alone cannot establish direct molecular interaction. Stable co-isolation may result from indirect association through large multiprotein assemblies, chromatin scaffolds, nuclear matrix components, or incomplete dissociation during extraction.

   Throughout the revised manuscript, the language has been moderated in some sections, but statements implying direct interaction, physical tethering, or structural anchoring remain stronger than warranted by the evidence presented. The biochemical data support stable association but not direct molecular interaction.

   I therefore recommend that the authors consistently use terminology such as "association", "co-fractionation", or "structural proximity" unless additional biochemical evidence (e.g., crosslinking, proximity labelling, co-immunoprecipitation under defined conditions, or ultrastructural visualization) is provided.

Author response #1

We thank the reviewer for this important comment and agree that the data presented support stable association, co-fractionation, and spatial proximity rather than direct molecular interaction.

In response, we have carefully revised the manuscript throughout to distinguish structural association from direct interaction. Specifically, we have removed or modified terminology implying direct molecular contact, physical tethering, structural anchoring, or mechanistic control where these conclusions are not directly supported by the data. Terms such as "direct interaction," "tethering," "anchoring," and related language have been replaced with more conservative descriptions including "association," "co-fractionation," "spatial association," "structural proximity," and "chromatin-associated localization," where appropriate.

Accordingly, the title, Abstract, Results, figure legends, and Discussion have been revised to ensure that the conclusions remain proportional to the experimental evidence and consistently distinguish structural association from direct molecular interaction.

We have also revised the interpretation of the biochemical fractionation experiments. The manuscript now explicitly describes these data as demonstrating co-fractionation and stable association of TUBG-containing structures with centromere-associated material under the extraction conditions used, rather than direct molecular interaction. Likewise, interpretations

Telephone Int +46 40338391 (direct)
Postal address Lund University, Division for Molecular Pathology, SUS-Malmö, 59 Jan Waldenström street
Se-205 02 Malmö, Sweden
E-mail maria.alvarado-kristensson@med.lu.se

LUND UNIVERSITY
Faculty of Medicine

Ph.D. Maria Alvarado Kristensson
Division for Molecular Pathology
Inga Marie Nilsson street 53
Se-214 28 Malmö, Sweden
maria.alvarado-kristensson@med.lu.se

of the imaging data have been revised to emphasize spatial association and structural proximity rather than direct physical binding. Similarly, we have revised the interpretation of the ChIP-seq analyses to emphasize chromatin association and centromere-proximal occupancy rather than direct binding to centromeric chromatin.

Our intention is not to claim that TUBG directly binds centromeric chromatin. Rather, we conclude that TUBG-containing structures are recurrently associated with centromere-defined regions across multiple experimental approaches, including ChIP-seq analyses, 3D volumetric imaging, live-cell imaging, and biochemical co-fractionation. We agree that additional approaches such as proximity labeling, co-immunoprecipitation under defined conditions, crosslinking studies, or ultrastructural analyses would be required to establish direct molecular interaction.

Accordingly, the revised manuscript now presents TUBG-containing structures as being associated with centromere-defined regions and nuclear organization, while avoiding interpretations that exceed the resolution of the current biochemical and imaging data.

Reviewer #1:

2. Evidence for Functional Control of Centromere Organization Remains Limited
   The authors propose that TUBG acts as a “mechanical buffer”, “kinematic scaffold”, and regulator of centromere homeostasis. While the mobility data following TUBG depletion are intriguing, they do not by themselves demonstrate organizational control. The central observation remains that TUBG depletion alters centromere fluorescence intensity and reduces centromere mobility. These findings are compatible with several interpretations, including indirect effects on nuclear architecture, chromatin mechanics, cell-cycle progression, DNA damage signalling, or centromere organization itself. The revised manuscript continues to present a model in which TUBG actively controls centromere homeostasis. In my opinion, the data currently support a more conservative conclusion: TUBG contributes to the structural environment associated with centromere positioning and mobility.
   The distinction between contribution and control is important and should be reflected more carefully throughout the Discussion.

Author response #2

We thank the reviewer for this important point. We agree that the current data support contribution and association more strongly than direct organizational control. Our intention was not to imply that TUBG directly regulates centromere homeostasis, but rather that alterations in TUBG levels are associated with changes in centromere-associated phenotypes, including fluorescence intensity, mobility, and responses to cellular stress.

Accordingly, we have revised the Discussion throughout to avoid language implying that TUBG actively controls centromere homeostasis. Terms such as “mechanical buffer,” “kinematic scaffold,” and other wording suggestive of direct organizational control have been

Telephone Int +46 40338391 (direct)
Postal address Lund University, Division for Molecular Pathology, SUS-Malmö, 59 Jan Waldenström street
Se-205 02 Malmö, Sweden
E-mail maria.alvarado-kristensson@med.lu.se

LUND UNIVERSITY
Faculty of Medicine

Ph.D. Maria Alvarado Kristensson
Division for Molecular Pathology
Inga Marie Nilsson street 53
Se-214 28 Malmö, Sweden
maria.alvarado-kristensson@med.lu.se

removed or moderated. We now describe TUBG-containing structures as contributing to the structural environment associated with centromere positioning and mobility rather than acting as direct regulators of centromere organization.

We have also explicitly acknowledged alternative interpretations of the observed phenotypes. In particular, we now state that although reduced TUBG levels alter centromere fluorescence intensity and mobility, the present data do not distinguish whether these phenotypes arise through direct effects on centromere-associated structures or indirectly through broader changes in nuclear architecture, chromatin organization, cell-cycle progression, or DNA damage signaling.

Our conclusions have therefore been revised to remain proportional to the experimental evidence and now emphasize association and contribution rather than direct organizational control.

Reviewer #1:

3. Interpretation of Centromere "Compaction"

I remain unconvinced by the interpretation that increased centromere fluorescence intensity directly reflects centromere compaction or condensation.

The authors use the terms "compaction", "condensation", and "stalling" interchangeably in several sections. However, fluorescence intensity measurements alone do not provide direct information regarding chromatin density or volumetric compaction.

Demonstrating compaction would require complementary measurements, such as: volumetric reconstruction of individual centromeres, changes in centromere volume, chromatin ultrastructure analysis, or orthogonal biochemical measurements.

Unless such data are available, I recommend describing the phenotype more neutrally as increased fluorescence intensity associated with reduced mobility.

Author response #3

We thank the reviewer for this important comment and agree that fluorescence intensity measurements alone do not directly demonstrate chromatin compaction or condensation.

Our intention was to describe the observed phenotype of increased centromere fluorescence intensity accompanied by reduced mobility rather than to claim direct evidence of chromatin compaction. We agree that demonstrating centromere compaction would require additional analyses, such as volumetric measurements, ultrastructural approaches, or orthogonal biochemical assays, which are beyond the scope of the present study.

Accordingly, we have revised the manuscript throughout to remove or moderate terminology such as "compaction," "condensation," and "stalling" where these terms implied structural conclusions not directly supported by the data. We now describe the phenotype more conservatively as increased centromere fluorescence intensity associated with reduced mobility. We have also revised the Discussion to explicitly acknowledge that fluorescence intensity measurements alone do not distinguish between chromatin compaction, chromatin

Telephone Int +46 40338391 (direct)
Postal address Lund University, Division for Molecular Pathology, SUS-Malmö, 59 Jan Waldenström street
Se-205 02 Malmö, Sweden
E-mail maria.alvarado-kristensson@med.lu.se

LUND UNIVERSITY
Faculty of Medicine

Ph.D. Maria Alvarado Kristensson
Division for Molecular Pathology
Inga Marie Nilsson street 53
Se-214 28 Malmö, Sweden
maria.alvarado-kristensson@med.lu.se

density, changes in centromere volume, or other factors affecting centromere-associated signal intensity.

Our conclusions have therefore been revised to remain proportional to the experimental evidence while preserving the underlying observations.

Reviewer #1:
4. Cryo-ET Concerns Are Not Fully Addressed
I appreciate the authors' explanation regarding the limitations of cryo-electron tomography for studying dynamic processes. I agree that cryo-ET cannot directly address kinetic behaviour. However, this was not the primary motivation behind my suggestion. The rationale was to obtain higher-resolution structural evidence supporting the proposed physical connections between γ-tubules, centrosomes, centromeres, and the nuclear compartment.
The authors correctly point out the volumetric limitations of cryo-ET. Nevertheless, the absence of such structural validation means that several architectural conclusions remain inferential. The new Airyscan analyses improve the visualization of these structures but do not provide the molecular resolution required to distinguish direct contacts from close spatial proximity.
I therefore do not consider this issue resolved, although I acknowledge that it may be beyond the scope of the current study.

Author response #4

We thank the reviewer for this thoughtful comment and agree that cryo-electron tomography or related ultrastructural approaches could provide higher-resolution structural information.

We also agree that the current data do not resolve direct physical contacts at molecular resolution. Accordingly, we have further revised the manuscript to avoid interpretations implying direct structural connections and instead describe the observed relationships as spatial associations, structural proximity, and co-fractionation where appropriate.

Our intention is not to claim that the present imaging and biochemical data demonstrate direct physical continuity between these structures. Rather, we interpret the combined evidence from Airyscan imaging, 3D volumetric reconstruction, live-cell imaging, immunoelectron microscopy, and biochemical co-fractionation as supporting a model of recurrent spatial association among TUBG-containing structures, centromere-defined regions, and the nuclear compartment.

To reflect this limitation, we have revised the Discussion to explicitly state that the proposed architectural model remains inferential and that higher-resolution ultrastructural approaches, including cryo-electron tomography, will be required to determine whether direct physical continuity exists among these structures.

Reviewer #1: 5. ChIP-seq Interpretation
The additional centromere-centered analyses strengthen the manuscript and demonstrate

Telephone Int +46 40338391 (direct)
Postal address Lund University, Division for Molecular Pathology, SUS-Malmö, 59 Jan Waldenström street
Se-205 02 Malmö, Sweden
E-mail maria.alvarado-kristensson@med.lu.se

LUND UNIVERSITY
Faculty of Medicine

Ph.D. Maria Alvarado Kristensson
Division for Molecular Pathology
Inga Marie Nilsson street 53
Se-214 28 Malmö, Sweden
maria.alvarado-kristensson@med.lu.se

reproducible TUBG-associated signal around annotated centromeric regions.
However, the authors should be careful not to overinterpret these data. ChIP-seq demonstrates chromatin association but does not distinguish whether TUBG directly binds centromeric chromatin, associates through intermediary proteins, or reflects broader chromatin-domain organization. The current interpretation should therefore remain descriptive.

Author response #5

We thank the reviewer for this important comment and agree that ChIP-seq data alone do not distinguish between direct binding to centromeric chromatin, indirect association through intermediary proteins, or broader chromatin-domain organization.

Accordingly, we have revised the interpretation of the ChIP-seq analyses throughout the manuscript to remain descriptive. Language implying direct centromeric binding has been replaced with more conservative terminology such as "chromatin association," "centromere-associated signal," and "centromere-proximal occupancy." We have also explicitly clarified in the Results section that the observed ChIP-seq signal does not distinguish between direct chromatin binding, association through intermediary protein complexes, or broader chromatin-domain organization.

Our interpretation of the ChIP-seq data is therefore restricted to demonstrating reproducible TUBG-associated signal near annotated centromere regions rather than direct interaction with centromeric DNA.

Reviewer #1:
- Minor Comments

1. Reference Usage
The revised manuscript has reduced the proportion of self-citations and the situation has improved considerably. However, several instances remain where review articles are cited instead of primary literature, particularly when supporting specific experimental observations. The reference list should be carefully re-examined to ensure that primary evidence is cited whenever possible. For example:
Page 4 "Altered expression and localization of TUBG have been documented in several cancers, including gliomas, medulloblastomas, glioblastomas, myelomas, hepatocellular carcinoma, non-small cell carcinoma, and breast cancer." The statement is currently supported by a self-citation to a review article. More appropriate primary references are available and should be cited instead. Examples include DOI: 10.1097/01.jnen.0000229235.20995.6e, DOI: 10.1007/s11064-007-9321-1, and DOI: 10.1002/jcp.22077.

Author response #6

We thank the reviewer for this helpful suggestion. We agree that primary literature should be cited wherever possible when supporting specific experimental observations. Accordingly, we have revised the reference list throughout the manuscript and replaced review citations with

Telephone Int +46 40338391 (direct)
Postal address Lund University, Division for Molecular Pathology, SUS-Malmö, 59 Jan Waldenström street
Se-205 02 Malmö, Sweden
E-mail maria.alvarado-kristensson@med.lu.se

LUND UNIVERSITY
Faculty of Medicine

Ph.D. Maria Alvarado Kristensson
Division for Molecular Pathology
Inga Marie Nilsson street 53
Se-214 28 Malmö, Sweden
maria.alvarado-kristensson@med.lu.se

primary studies where appropriate. For the statement regarding altered TUBG expression and localization in cancer, we have incorporated the primary references suggested by the reviewer, together with additional primary literature where relevant.

Reviewer #1:
Page 6 "U2OS cells were transfected with X-fect (Takara Bio Europa, Cat. No. 631317) according to the manufacturer's instructions." If the transfection was performed strictly according to the manufacturer's protocol, the rationale for including a self-citation is unclear. Please clarify whether the cited reference describes modifications to the standard protocol or remove the citation if it is not required.

Author response #7

We thank the reviewer for this observation and agree. As the transfection was performed according to the manufacturer's protocol without modification, the self-citation was unnecessary and has been removed.

Reviewer #1:
Page 22
"In our previously published work, we demonstrated that cellular TUBG forms various structural arrangements, including distinctive thread-like γ-strings, measuring 4 to 6 nm in diameter." Reference 37 does not support this statement. The cited work is focused on the identification and isolation of TUBG–pericentrin complexes and does not describe the formation or characterization of γ-strings.

Author response #8

We thank the reviewer for this observation. We agree that Dictenberg et al. (1998) does not specifically describe γ-strings as defined in our later work, nor does it report their diameter. However, we cited this study because it provides early evidence that γ-tubulin is organized into fibrillar/lattice-like structures within the pericentriolar matrix.

To avoid overstating the scope of this reference, we have revised the text to distinguish between the fibrillar γ-tubulin organization described by Dictenberg et al. and the γ-string structures characterized in our subsequent studies. The statement regarding γ-strings and their reported diameter is now supported by the appropriate primary references.

Reviewer #1:
"Notably, γ-strings play a key role in the transport of cytosolic PCNA to replication origins." Reference 38 does not appear to support this statement. The cited study focuses on the role of the chaperonin CCT in the formation of γ-tubulin filaments and does not address the transport of cytosolic PCNA by γ-strings.

Author response #9

Telephone Int +46 40338391 (direct)
Postal address Lund University, Division for Molecular Pathology, SUS-Malmö, 59 Jan Waldenström street
Se-205 02 Malmö, Sweden
E-mail maria.alvarado-kristensson@med.lu.se

LUND UNIVERSITY
Faculty of Medicine

Ph.D. Maria Alvarado Kristensson
Division for Molecular Pathology
Inga Marie Nilsson street 53
Se-214 28 Malmö, Sweden
maria.alvarado-kristensson@med.lu.se

We thank the reviewer for this observation. We agree that the cited study by Pouchucq et al. does not directly address the transport of cytosolic PCNA to replication origins. Our intention in citing this reference was to support the formation and assembly of γ-tubulin filamentous structures (γ-strings), as the study describes the role of the chaperonin CCT in the formation of γ-tubulin filaments in vitro.

To avoid confusion, we have revised the manuscript and citation structure. The statement regarding the association of γ-strings with PCNA recruitment and replication-associated processes is now supported by the appropriate primary reference(s), whereas the Pouchucq et al. study is cited specifically in relation to γ-string/γ-tubulin filament assembly.

Reviewer #1:
"In H9-NSC cells, we additionally observed TUBG-foci." Reference 39 is a review article and does not appear to provide direct experimental evidence supporting this specific observation. A primary reference would be more appropriate.

Author response #10

We thank the reviewer for this observation. We agree that primary literature is generally preferable when citing specific experimental observations. In this case, however, the cited article represents the first publication in which TUBG-foci were experimentally described by our group, including microscopy-based characterization and live-cell imaging demonstrating their dynamic behavior. We therefore cited this study as the original source of the observation.

To avoid confusion, we have revised the text to clarify that the reference is cited specifically in support of the initial description and characterization of TUBG-foci rather than as a general review of the field.

Reviewer #1:
Page 34."This network includes not only the previously noted γ-strings but also γ-tubules, which are cytosolic fibers with diameters between 20 and 25 nm." Reference 45 does not appear to support this statement. The cited work addresses the targeting of γ-tubulin to microtubule domains rather than the formation or characterization of γ-strings or γ-tubules.

Author response #11

We thank the reviewer for this observation. We agree that the cited study was not intended to serve as the primary reference for the structural characterization of γ-tubules or their reported diameter. The reference was included because γ-tubule-like structures are visible in Figure 1 of that study and therefore provide experimental evidence for the presence of these γ-tubulin-containing fibers.

To avoid ambiguity, we have revised the text and citation structure. References supporting the structural characterization and reported dimensions of γ-tubules are now cited separately from studies that primarily document their visualization or localization.

Reviewer #1:

Telephone Int +46 40338391 (direct)
Postal address Lund University, Division for Molecular Pathology, SUS-Malmö, 59 Jan Waldenström street
Se-205 02 Malmö, Sweden
E-mail maria.alvarado-kristensson@med.lu.se

LUND UNIVERSITY
Faculty of Medicine

Ph.D. Maria Alvarado Kristensson
Division for Molecular Pathology
Inga Marie Nilsson street 53
Se-214 28 Malmö, Sweden
maria.alvarado-kristensson@med.lu.se

Page 38. "The functional nature of this docking is further substantiated in U2OS cells treated with 5 µM cisplatin, a DNA-damaging agent that induces genomic stress." This statement would benefit from a direct reference describing cisplatin-induced DNA damage and genomic stress, such as: Wang D, Lippard SJ. Cellular processing of platinum anticancer drugs. Nat Rev Drug Discov. 2005;4:307–320. The current self-citation (reference 4) is not the most appropriate source for this well-established concept.

Author response #12

We thank the reviewer for this helpful suggestion and agree that a primary reference describing cisplatin-induced DNA damage and genomic stress is more appropriate in this context. Accordingly, we have removed the previous self-citation and replaced it with the suggested reference.

Reviewer #1:
2. Statistical Reporting
The manuscript now reports the number of cells and experiments more consistently. However, for all quantitative analyses, the authors should clearly distinguish: number of biological replicates, number of cells, number of tracks, and number of segmented centromeres.
This distinction remains difficult to follow in several figures.

Author response #13

We thank the reviewer for this comment. We agree that the distinction between biological replicates and the different analytical units should be clearly identifiable throughout the manuscript.

The quantitative analyses in this study were performed using distinct primary units depending on the experiment, including individual cells, segmented centromere-defined volumes, centromere tracks, centrosomes, and image frames. These quantities are reported in the corresponding figure legends and Results sections, together with the number of independent biological replicates.

To improve clarity, we have carefully revised the figure legends and relevant Results sections to ensure that the primary unit of analysis and the number of biological replicates are stated consistently throughout the manuscript.

Reviewer #1:
3. Graphical Presentation
The issues previously raised regarding colour schemes and symbol coding remain only partially resolved. In several figures, it remains difficult to determine: which symbols correspond to which treatment, which data points are paired, and how the statistical comparisons were performed. For example:
Figure 2A. Why is the centromere signal displayed in red in the CPM panels while it is shown in purple throughout the remainder of the figure set? Apart from making interpretation more

Telephone Int +46 40338391 (direct)
Postal address Lund University, Division for Molecular Pathology, SUS-Malmö, 59 Jan Waldenström street
Se-205 02 Malmö, Sweden
E-mail maria.alvarado-kristensson@med.lu.se

LUND UNIVERSITY
Faculty of Medicine

Ph.D. Maria Alvarado Kristensson
Division for Molecular Pathology
Inga Marie Nilsson street 53
Se-214 28 Malmö, Sweden
maria.alvarado-kristensson@med.lu.se

difficult for colour-blind readers, the sudden change in colour coding is confusing and should be justified or standardized.

Author response #14

We thank the reviewer for this observation. We agree that consistent color coding is important for readability and accessibility. The centromere signal is displayed in magenta throughout the primary images in the manuscript to improve visualization and accessibility for color-blind readers.

In contrast, the CPM panels are generated using the ImageJ colocalization plugin, which operates on the original red and green channel assignments used for the colocalization analysis and displays overlap according to this convention. Thus, the CPM panels represent a derived analytical output rather than the color scheme used for figure presentation.

To avoid confusion, we have revised the figure legend to explicitly explain the color assignments used in the CPM panels and to distinguish them from the color coding used in the corresponding immunofluorescence images.

Reviewer #1:

Figure 2B. Why was a Wilcoxon matched-pairs signed-rank test used instead of a paired t-test? Please justify the choice of statistical test. The graph symbols are difficult to interpret. The use of filled and open circles together with upward- and downward-pointing triangles is not adequately explained. It is unclear which symbols correspond to cisplatin-treated and untreated samples, and which data points are paired in the statistical analysis.

Figure 3C. As in Figure 2A, the red-green colour coding is not clearly explained and is difficult to interpret. A more consistent colour scheme throughout the manuscript would improve readability.

Figure 7F. As in Figure 2B, the graphical symbols are not sufficiently explained. It is unclear which symbols correspond to cisplatin-treated cells, rotenone-treated cells, or controls, and which groups are being compared statistically. A clearer legend is required.

The figure legends should be revised to improve clarity.

Author response #16

We thank the reviewer for these helpful comments. We have revised the figure legends to clarify color coding, CPM channel assignments, symbol coding, paired data points, and statistical comparisons. For CPM panels, we now explain that the red/green display reflects the original analytical channel assignments used by the ImageJ colocalization plugin, whereas magenta is used for centromeres in the merged presentation images. We have also clarified the rationale for the Wilcoxon matched-pairs signed-rank test, including the use of non-parametric statistics following assessment of data normality, and have expanded the figure legends to explain the graph symbols in Figs. 2B and 7F and the statistical comparisons shown.

Reviewer #1:

4. Figure 8

Telephone Int +46 40338391 (direct)
Postal address Lund University, Division for Molecular Pathology, SUS-Malmö, 59 Jan Waldenström street
Se-205 02 Malmö, Sweden
E-mail maria.alvarado-kristensson@med.lu.se

LUND UNIVERSITY
Faculty of Medicine

Ph.D. Maria Alvarado Kristensson
Division for Molecular Pathology
Inga Marie Nilsson street 53
Se-214 28 Malmö, Sweden
maria.alvarado-kristensson@med.lu.se

Although Figure 8 has improved, the schematic model remains visually dense and difficult to follow. Simplification would greatly enhance accessibility for readers unfamiliar with the γ-tubulin field.

Author response #17

We thank the reviewer for this suggestion. We agree that the original schematic was visually dense and could be difficult to follow for readers unfamiliar with the γ-tubulin field. Accordingly, we have simplified Figure 8 by reducing the number of repeated structural elements, decreasing visual clutter, and emphasizing the principal components and relationships relevant to the proposed model. The revised schematic retains the key features of the TUBG meshwork while improving readability and accessibility.

Overall Assessment

The manuscript presents several interesting observations and has improved substantially during revision. The additional quantitative analyses and biochemical experiments strengthen the study. However, the central mechanistic conclusions continue to extend beyond what is directly demonstrated by the data.

In its current form, I believe the manuscript supports a model in which TUBG is associated with centromere-containing structures and influences their spatial behaviour. The stronger claims regarding direct interaction, structural tethering, and organizational control remain insufficiently supported.

I therefore recommend further revision focused on moderating mechanistic interpretations, clarifying statistical reporting, and ensuring that conclusions remain proportional to the experimental evidence.

Author response #18

We thank the reviewer for the constructive evaluation of our work. In response to these concerns, we have revised the manuscript throughout to moderate mechanistic interpretations, distinguish association from direct interaction, clarify statistical reporting, simplify figure presentation, and ensure that the conclusions remain proportional to the experimental evidence. We believe that these revisions substantially strengthen the manuscript and appreciate the reviewer's guidance.

Reviewer #2 (Remarks to the Author):

Reviewer #2: I appreciate the authors' efforts in carefully addressing the concerns raised during review. In particular, the addition of biochemical fractionation experiments substantially strengthens the manuscript and provides support for the microscopy-based observations. However, given that the manuscript is entitled "The Tubulin Meshwork Regulates Centromere Kinematics and Micronucleus Homeostasis," I find the functional significance of the reported TUBG-centromere association remains somewhat unclear. If the manuscript were intended primarily to describe a structural association between the TUBG meshwork and centromeres,

Telephone Int +46 40338391 (direct)
Postal address Lund University, Division for Molecular Pathology, SUS-Malmö, 59 Jan Waldenström street
Se-205 02 Malmö, Sweden
E-mail maria.alvarado-kristensson@med.lu.se

LUND UNIVERSITY
Faculty of Medicine

Ph.D. Maria Alvarado Kristensson
Division for Molecular Pathology
Inga Marie Nilsson street 53
Se-214 28 Malmö, Sweden
maria.alvarado-kristensson@med.lu.se

the current data might be sufficient. However, the use of the term "regulates" implies a functional role, and I believe the biological consequences of this association for centromere function remain insufficiently explored.

Author response #1

We thank the reviewer for this thoughtful and important comment. We agree that the term "regulates" may imply a degree of mechanistic control that is not fully established by the current data. Our intention was not to claim that TUBG functions as a direct molecular regulator of centromere activity, but rather to describe the reproducible association of the TUBG meshwork with centromere-associated chromatin and the phenotypic consequences observed following perturbation of TUBG.

To address this concern, we have extensively revised the manuscript to more clearly distinguish structural association from mechanistic regulation. Specifically, we have modified the title, Abstract, Results, and Discussion to avoid language implying direct control of centromere function. Throughout the manuscript, terms such as "regulates," "controls," "tethering," and "direct interaction" have been replaced with more conservative descriptions including "association," "contribution," "spatial association," and "chromatin-proximal localization," where appropriate. We have also revised interpretations related to centromere "compaction" and now describe the phenotype more conservatively as increased centromere fluorescence intensity accompanied by reduced mobility.

In addition, we have expanded the Discussion to explicitly acknowledge that the observed effects may arise either from local TUBG-associated structures or from broader changes in nuclear architecture and cytoskeletal organization. We now emphasize that our data support a model in which TUBG-containing assemblies are recurrent structural components of centromere-associated regions and are associated with centromere positioning, mobility, and stress-induced reorganization, rather than demonstrating direct molecular regulation of centromere function.

Importantly, the underlying observations remain unchanged. Multiple independent approaches—including ChIP-seq analysis, 3D volumetric imaging, live-cell imaging, biochemical co-fractionation, TUBG depletion experiments, and phospho-mutant analyses—demonstrate a reproducible association between TUBG-containing structures and centromere-associated chromatin. Furthermore, perturbation of TUBG alters centromere-associated phenotypes, including fluorescence intensity, mobility, and responses to cellular stress, while stress-induced remodeling of the TUBG meshwork correlates with changes in centromere organization and micronucleus-associated structures.

We therefore conclude that the data support a role for TUBG-containing structures as structural components associated with centromere organization, centromere-associated dynamics, and micronucleus homeostasis, while acknowledging that the precise molecular mechanisms underlying these associations remain to be determined.

Telephone Int +46 40338391 (direct)
Postal address Lund University, Division for Molecular Pathology, SUS-Malmö, 59 Jan Waldenström street
Se-205 02 Malmö, Sweden
E-mail maria.alvarado-kristensson@med.lu.se

LUND UNIVERSITY
Faculty of Medicine

Ph.D. Maria Alvarado Kristensson
Division for Molecular Pathology
Inga Marie Nilsson street 53
Se-214 28 Malmö, Sweden
maria.alvarado-kristensson@med.lu.se

Reviewer #2:
Regarding my comment that "Quantitative analysis is required to show the proportion of cells exhibiting the described phenotype," the authors state that "TUBG was detectable within all analyzed centromere-defined volumes (n = 100 per condition) across all cell lines studied." However, this result appears difficult to reconcile with the representative images shown in Fig. 1D, where several centromeric foci do not appear to exhibit an obvious TUBG signal. The authors should clarify how "detectable" was defined in their analysis and provide sufficient methodological detail to explain this apparent discrepancy between the quantitative measurements and the representative images.

Author response #2

We thank the reviewer for this important observation. We agree that the representative composite image shown in Fig. 1D may give the impression that some centromeric foci do not exhibit obvious TUBG signal. However, the quantitative analysis was not based on visual inspection of maximum-intensity projections.

As clarified in the revised Methods section, centromeres were first segmented in three dimensions using the anti-centromere channel, generating centromere-defined 3D ROIs. These ROIs were subsequently redirected to the corresponding raw TUBG channel across the complete Airyscan Z-stack, and fluorescence intensity was quantified throughout the entire segmented volume. Lamin B was analyzed using the identical ROIs and workflow as an internal control for non-specific fluorescence signal within the segmented volumes.

We also clarify that the primary unit of analysis was the individual centromere-defined volume rather than a binary cell-level score. All segmentable centromeres within the analyzed cells were included when an individual centromere volume could be established from the anti-centromere channel. Thus, the analysis reports the distribution of TUBG fluorescence intensity across the analyzed centromere population.

In the revised manuscript, we now explicitly define detectability as the extraction of measurable raw TUBG fluorescence intensity from a segmented centromere-defined 3D ROI, interpreted relative to Lamin B measured in the same ROIs, followed by comparison with Lamin B measured in the same ROIs.

Importantly, the statement that TUBG was detected within centromere-defined volumes refers to quantitative extraction of fluorescence intensity from complete 3D centromere ROIs and not to visually apparent overlap in representative projections. Because the displayed images are partial maximum-intensity projections and composites, weak or spatially restricted TUBG signal contributing to the quantitative measurements may not be visually apparent in every projected centromeric focus.

We have revised the Methods, Results, and figure legend to clarify this point. We further note that the same 3D ROI-based workflow was applied consistently across the analyzed cell types in the study, including H9-NSCs, U2OS cells, primary normal kidney epithelial cells, and primary ccRCC cells.

Telephone Int +46 40338391 (direct)
Postal address Lund University, Division for Molecular Pathology, SUS-Malmö, 59 Jan Waldenström street
Se-205 02 Malmö, Sweden
E-mail maria.alvarado-kristensson@med.lu.se

LUND UNIVERSITY
Faculty of Medicine

Ph.D. Maria Alvarado Kristensson
Division for Molecular Pathology
Inga Marie Nilsson street 53
Se-214 28 Malmö, Sweden
maria.alvarado-kristensson@med.lu.se

Reviewer #2:
Regarding the response to my comment on page 31, I remain somewhat uncertain about the proposed mechanism of micronucleus formation. While the authors suggest that cellular stress is responsible, it remains unclear to me whether stress-induced mitotic defects or mitotic arrest might contribute to the formation of micronuclei. If such events occur, they may represent an important intermediate step linking cellular stress to micronucleus formation. Therefore, it would be helpful for the authors to quantify and report the extent of mitotic defects and/or mitotic arrest observed under these

Author response #3

We thank the reviewer for this important point. We agree that stress-induced mitotic defects and/or mitotic arrest may represent intermediate mechanisms linking cellular stress to micronucleus formation. Because overt mitotic figures were rare in the primary kidney and ccRCC cultures, the present fixed-cell datasets used in this study did not permit a robust quantitative assessment of mitotic arrest or abnormal mitoses. We have therefore revised the Discussion (eighth paragraph) to explicitly acknowledge that the origin of micronuclei was not directly determined in the present study and that stress-induced mitotic defects remain a plausible contributor to micronucleus formation. Our conclusions have been restricted to the recruitment and organization of TUBG-containing structures after micronuclei are present.

Telephone Int +46 40338391 (direct)
Postal address Lund University, Division for Molecular Pathology, SUS-Malmö, 59 Jan Waldenström street
Se-205 02 Malmö, Sweden
E-mail maria.alvarado-kristensson@med.lu.se

LUND UNIVERSITY
Faculty of Medicine

Malmö 18 July 2026

Ph.D. Maria Alvarado Kristensson
Division for Molecular Pathology
Inga Marie Nilsson street 53
Se-214 28 Malmö, Sweden
maria.alvarado-kristensson@med.lu.se

Point-by-point reply
****

Response to Reviewers

Manuscript Title: The TUBG meshwork is associated with centromere dynamics and micronucleus-associated organization **Manuscript ID:** COMMSBIO-25-7197B

We thank the Editor and Reviewers for their careful assessment of the revised manuscript. We are grateful that Reviewer 1 considers the previous concerns fully addressed. In response to the remaining concerns raised by Reviewer 2, we have further revised the manuscript to distinguish fluorescence-intensity measurements from evidence of centromere function and to remove the claim that TUBG is detectable in 100% of centromeres. We have also clarified the quantitative workflow in the Materials and Methods and revised the corresponding Results, Discussion, figure legends, and Supplementary Information.

REVIEWERS' COMMENTS:

Reviewer #1:
I have carefully examined the authors' rebuttal and the revised version of the manuscript. Overall, I believe the authors have satisfactorily addressed all of the concerns raised in my previous review. In particular, they have consistently moderated the interpretation of their data, distinguishing structural association from direct molecular interaction, avoiding overinterpretation of the functional implications, and clarifying both the statistical reporting and the presentation of the figures. The revised conclusions now appear to be appropriately supported by the experimental evidence. Based on the revised manuscript and the authors' responses, I consider that my previous concerns have been adequately addressed and I have no further comments.
Author response #1
We thank the Reviewer for the careful reassessment of our manuscript and for recognizing the revisions made to distinguish spatial or structural association from direct molecular interaction. We are pleased that the revised interpretation, statistical reporting, figure presentation, and conclusions are now considered adequately supported by the experimental evidence.

Reviewer #2—General comment
I appreciate the authors' efforts in addressing my previous comments and revising the manuscript accordingly. Several of my concerns have been satisfactorily addressed. However,

Telephone Int +46 40338391 (direct)
Postal address Lund University, Division for Molecular Pathology, SUS-Malmö, 59 Jan Waldenström street
Se-205 02 Malmö, Sweden
E-mail maria.alvarado-kristensson@med.lu.se

LUND UNIVERSITY
Faculty of Medicine

Ph.D. Maria Alvarado Kristensson
Division for Molecular Pathology
Inga Marie Nilsson street 53
Se-214 28 Malmö, Sweden
maria.alvarado-kristensson@med.lu.se

I find the response to Comment #2 particularly problematic. In my opinion, this issue directly affects the interpretation of the central findings of the study. Moreover, if the same definition of "detectable" has been applied throughout the manuscript, this concern may extend beyond Figure 1 and could influence the interpretation of other analyses that rely on similar quantitative measurements.

Response to Reviewer #2—General comment

We thank the Reviewer for identifying this remaining concern. We agree that the previous use of the term "detectable" could be interpreted as a binary classification and could therefore overstate the conclusions derived from fluorescence-intensity measurements.

We have now removed the claim that TUBG is detectable in 100% of centromeres and have revised the corresponding terminology throughout the manuscript, figure legends, and Supplementary Information. TUBG fluorescence is now described as a continuous quantitative measurement within segmented centromere-defined 3D regions of interest rather than as a binary positive-versus-negative classification.

The Materials and Methods have been revised to clarify that:

1. *centromeres were segmented objectively in three dimensions using the anti-centromere channel;*
2. *the resulting centromere-defined 3D ROIs were applied to the corresponding raw TUBG channel throughout the complete Z-stack;*
3. *TUBG fluorescence intensity was analyzed as a continuous variable;*
4. *no binary threshold was used to classify centromeres as TUBG-positive or TUBG-negative; and*
5. *the analysis was not used to establish universal TUBG detectability across individual centromeres.*

We also clarified that Lamin B was used as a paired comparator only for the analysis presented in Fig. 1e. In that analysis, TUBG and Lamin B fluorescence intensities were measured within the same segmented centromere-defined 3D ROIs. Lamin B was not used as a comparator in the other centromere-intensity analyses, in which TUBG fluorescence was measured directly within centromere-defined volumes.

Representative images are partial maximum-intensity projections and therefore do not display the complete 3D volumes used for quantification. The revised text now states that these images are provided for illustration, whereas quantitative measurements were obtained from the complete Z-stacks. Importantly, this clarification is not used to support a universal detectability claim, which has been removed.

These revisions do not change the experimental data or analytical workflow. They restrict the interpretation to the continuous fluorescence-intensity measurements directly supported by the analysis.

Reviewer #2—Comment 1

Telephone Int +46 40338391 (direct)
Postal address Lund University, Division for Molecular Pathology, SUS-Malmö, 59 Jan Waldenström street
Se-205 02 Malmö, Sweden
E-mail maria.alvarado-kristensson@med.lu.se

LUND UNIVERSITY
Faculty of Medicine

Ph.D. Maria Alvarado Kristensson
Division for Molecular Pathology
Inga Marie Nilsson street 53
Se-214 28 Malmö, Sweden
maria.alvarado-kristensson@med.lu.se

I appreciate the authors' efforts to incorporate my suggestions and revise the manuscript accordingly. The revised text more accurately reflects the experimental findings and avoids implying a mechanistic role that is not fully supported by the data. However, I still have a minor concern. While TUBG depletion alters centromere-associated phenotypes, I am not convinced that the observed increase in centromere fluorescence intensity necessarily indicates an alteration of centromere function. Therefore, I remain somewhat cautious about interpreting these findings as evidence of functional consequences at the centromere level.

Response to Reviewer #2—Comment 1

We agree with the Reviewer that increased centromere fluorescence intensity does not, by itself, demonstrate altered centromere function. We have therefore further restricted the interpretation of this result throughout the manuscript.

The revised Results and Discussion now describe the phenotype as "increased centromere fluorescence intensity associated with reduced mobility." We no longer interpret the increased intensity as evidence of altered centromere function. We have also added an explicit statement that fluorescence-intensity measurements alone do not establish:

- *altered centromere function;*
- *chromatin compaction;*
- *chromatin condensation; or*
- *altered centromere volume.*

The revised Discussion states:

"Furthermore, fluorescence intensity measurements alone do not provide evidence for chromatin compaction, condensation, altered centromere function, or altered centromere volume. Accordingly, we interpret the phenotype conservatively as increased centromere fluorescence intensity associated with reduced mobility.", lines 542-545.

Related statements in the Abstract, Results, figure legends, and concluding Discussion have also been revised to describe associations with centromere positioning and mobility without implying functional consequences at the centromere level.

Reviewer #2—Comment 2

In their response, the authors explain that "weak or spatially restricted TUBG signal contributing to the quantitative measurements may not be visually apparent." I consider this problematic, as such signals are then used to support the conclusion that TUBG is detectable in 100% of centromeres. The conclusion appears to depend heavily on the definition of "detectable," which, in my opinion, is currently too permissive to support such a claim.

Response to Reviewer #2—Comment 2

We thank the Reviewer for raising this important point. Our previous statement that weak or spatially restricted TUBG signal may not be visually apparent was intended only to describe a limitation of the representative maximum-intensity projections and was not intended to define a criterion for TUBG detectability. Specifically, TUBG fluorescence or its spatial relationship

Telephone Int +46 40338391 (direct)
Postal address Lund University, Division for Molecular Pathology, SUS-Malmö, 59 Jan Waldenström street
Se-205 02 Malmö, Sweden
E-mail maria.alvarado-kristensson@med.lu.se

LUND UNIVERSITY
Faculty of Medicine

Ph.D. Maria Alvarado Kristensson
Division for Molecular Pathology
Inga Marie Nilsson street 53
Se-214 28 Malmö, Sweden
maria.alvarado-kristensson@med.lu.se

with a centromere may be confined to one or a few Z-planes and may therefore not be fully resolved or accurately represented when the Z-stack is collapsed into a two-dimensional maximum-intensity projection.

We agree, however, that fluorescence measurements that are not visually apparent in the projected images do not justify the conclusion that TUBG is detectable in 100% of centromeres. We have therefore removed this claim and the associated use of "detectability" as an analytical category.

The revised manuscript describes the analysis as a continuous measurement of raw TUBG fluorescence intensity within objectively segmented centromere-defined 3D ROIs. The centromere-defined ROIs were generated from the anti-centromere channel independently of the TUBG signal, and all individually resolvable and segmentable centromere-defined volumes within the analyzed cells were included. No binary threshold was applied to classify individual centromeres as TUBG-positive or TUBG-negative.

The revised Materials and Methods now state:

"TUBG fluorescence was analyzed as a continuous intensity variable, and no binary threshold was used to classify individual centromeres as TUBG-positive or TUBG-negative.", lines 690-692.

The Methods further clarify:

"Accordingly, the analysis reports continuous fluorescence-intensity measurements across the analyzed centromere-defined volumes and was not used to establish universal TUBG detectability across individual centromeres." lines 692-695.

For the analysis presented in Fig. 1e only, the same centromere-defined 3D ROIs were applied to the raw TUBG and Lamin B channels, permitting a paired comparison of their fluorescence intensities. This comparison is no longer interpreted as evidence that every centromere contains detectable TUBG.

*We have also revised the corresponding Results and figure legend to describe continuous measurements of raw TUBG fluorescence intensity within segmented centromere-defined 3D ROIs and, for Fig. 1e only, the paired comparison with Lamin B fluorescence intensity***,** *without making a universal positive/negative claim.*

Representative maximum-intensity projections are now explicitly described as illustrative, whereas quantitative measurements were obtained from the complete Z-stacks. The revised Methods state:

"Because representative images are displayed as partial maximum-intensity projections and composites, fluorescence signals or spatial relationships confined to individual Z-planes may not be fully resolved when the Z-stacks are collapsed into two-dimensional projections. The displayed images were therefore used for illustration, whereas quantitative conclusions were derived from the complete Z-stacks using the segmented 3D ROIs." lines 697-702.

To ensure consistency, we reviewed the manuscript, figure legends, Supplementary Figures, and Supplementary Movie descriptions and removed references to "100% detectability,"

Telephone Int +46 40338391 (direct)
Postal address Lund University, Division for Molecular Pathology, SUS-Malmö, 59 Jan Waldenström street
Se-205 02 Malmö, Sweden
E-mail maria.alvarado-kristensson@med.lu.se

LUND UNIVERSITY
Faculty of Medicine

Ph.D. Maria Alvarado Kristensson
Division for Molecular Pathology
Inga Marie Nilsson street 53
Se-214 28 Malmö, Sweden
maria.alvarado-kristensson@med.lu.se

"detectable in all centromeres," and equivalent universal claims. Other centromere-associated measurements are now described as continuous fluorescence-intensity measurements, spatial associations, or stress-associated changes, depending on the experiment.

Reviewer #2—Comment 3
I appreciate the authors' clarification and the restriction of their conclusions.
Response to Reviewer #2—Comment 3
We thank the Reviewer for acknowledging the clarification and the restricted interpretation of our findings. We have maintained this conservative interpretation throughout the revised manuscript.

Telephone Int +46 40338391 (direct)
Postal address Lund University, Division for Molecular Pathology, SUS-Malmö, 59 Jan Waldenström street
Se-205 02 Malmö, Sweden
E-mail maria.alvarado-kristensson@med.lu.se
