# Supplementary material for: The TUBG meshwork is associated with centromere dynamics and micronuclear organization
Source: Commun Biol. 2026 Sep 16;9:1206. doi: 10.1038/s42003-026-10880-y (PMC13582872; doi:10.1038/s42003-026-10880-y)
Supplement: Supplementary file 3 [file 42003_2026_10880_MOESM2_ESM.pdf]

Supplementary information

Chromosome	Start position	End position	Cytoband	Centromeric region
chr1	121500000	125000000	p11.1	acen *
chr1	125000000	128900000	q11	acen
chr10	38000000	40200000	p11.1	acen
chr10	40200000	42300000	q11.1	acen
chr11	51600000	53700000	p11.11	acen
chr11	53700000	55700000	q11	acen
chr12	33300000	35800000	p11.1	acen
chr12	35800000	38200000	q11	acen
chr13	16300000	17900000	p11.1	acen
chr13	17900000	19500000	q11	acen
chr14	16100000	17600000	p11.1	acen
chr14	17600000	19100000	q11.1	acen
chr15	15800000	19000000	p11.1	acen
chr15	19000000	20700000	q11.1	acen
chr16	34600000	36600000	p11.1	acen
chr16	36600000	38600000	q11.1	acen
chr17	22200000	24000000	p11.1	acen
chr17	24000000	25800000	q11.1	acen
chr18	15400000	17200000	p11.1	acen
chr18	17200000	19000000	q11.1	acen
chr19	24400000	26500000	p11	acen
chr19	26500000	28600000	q11	acen
chr2	90500000	93300000	p11.1	acen
chr2	93300000	96800000	q11.1	acen
chr20	25600000	27500000	p11.1	acen
chr20	27500000	29400000	q11.1	acen
chr21	10900000	13200000	p11.1	acen
chr21	13200000	14300000	q11.1	acen
chr22	12200000	14700000	p11.1	acen
chr22	14700000	17900000	q11.1	acen
chr3	87900000	91000000	p11.1	acen
chr3	91000000	93900000	q11.1	acen
chr4	48200000	50400000	p11	acen
chr4	50400000	52700000	q11	acen
chr5	46100000	48400000	p11	acen
chr5	48400000	50700000	q11.1	acen
chr6	58700000	61000000	p11.1	acen
chr6	61000000	63300000	q11.1	acen
chr7	58000000	59900000	p11.1	acen
chr7	59900000	61700000	q11.1	acen
chr8	43100000	45600000	p11.1	acen
chr8	45600000	48100000	q11.1	acen
chr9	47300000	49000000	p11.1	acen
chr9	49000000	50700000	q11	acen
chrX	58100000	60600000	p11.1	acen

chrX	60600000	63000000	q11.1	acen
chrY	11600000	12500000	p11.1	acen
chrY	12500000	13400000	q11.1	acen

*acen = acentromeric or central region of the centromere on a chromosome

Supplementary Table 1. **List of centromeres' genomic coordinates, Related to Figure 1**. The coordinates were retrieved from UCSC Genome Browser cytoband using function distance ToNearest from GenomicRanges.

A. Modified cell lines				
Cell line	Expressing	Co-expressing	Selection	Denoted
U2OS	*TUBG* shRNA	TUBG-GFP	Zeocin, G418 GFP	*TUBG*-shRNA-U2OS-TUBG1-GFP
U2OS	*TUBG* shRNA	TUBG-GFP, *Lamin B1*-mCherry	Zeocin, G418, GFP, mCherry	sh*TUBG*-TUBG-LMNB-U2OS
U2OS	*TUBG* shRNA	TUBG-GFP, mCherry-*NUP50*	Zeocin, G418, GFP, mCherry	*TUBG*-shRNA-U2OS-TUBG1-GFP-NUP50
U2OS	*TUBG* shRNA	TUBG-GFP, mCherry-CENP-A	Zeocin, G418, GFP, mCherry	*TUBG*-shRNA-U2OS-TUBG1-CENP-A
U2OS	*TUBG* shRNA	TUBG-GFP, PCNA-Chromobody-RFP	Zeocin, G418, GFP, RFP	*TUBG*-shRNA-U2OS-TUBG-GFP-Pcc-RFP
U2OS	*TUBG1* sgRNA Cas9-crispGFP	–	GFP	*TUBG1*-sgRNA-U2OS
U2OS	*TUBG1* sgRNA Cas9-crispGFP	$TUBG_{rest}^{1-335}$ (*TUBG1*sg RNA resistant)	GFP	*TUBG1*-sgRNA-U2OS-$TUBG1^{1-335}$
U2OS	*TUBG1* sgRNA Cas9-crispGFP	$TUBG^{336-451}$	GFP	*TUBG1*-sgRNA-U2OS-$TUBG1^{336-451}$

Supplementary Table 2. Description of modified cell lines used in this study

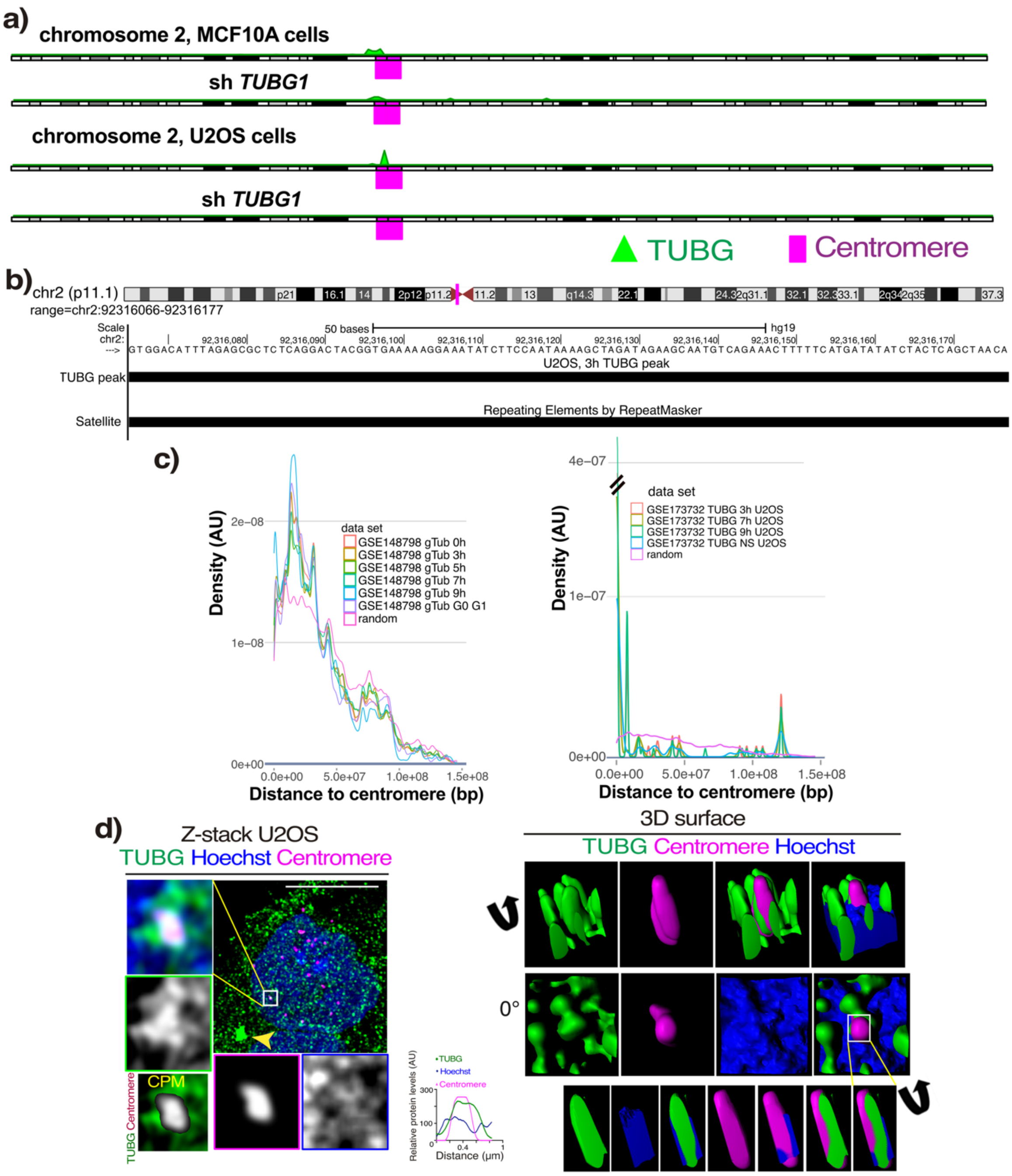

Supplementary Fig. 1 Nuclear localization and centromeric association of TUBG1.

a) ChIP-seq analysis showing TUBG-associated signal distribution across representative chromosomal regions on chromosome 2 in MCF10A cells (GSM2884570_gTub_no_shRNAi_2h) and U2OS cells (GSE173732_TUBG_3h_U2OS). A comparison is shown with cells exhibiting reduced TUBG expression (sh *TUBG*; MCF10A, GSM2884578_gTub_with_shRNAi_2h; U2OS, GSE173732_TUBG_3h_shgtub) [4,24].

b) Alignment of a TUBG-associated ChIP-seq signal peak with RepeatMasker annotations, identifying satellite DNA within centromeric and adjacent repetitive regions of chromosome 2 (range = chr2:92316066–92316177).

c) Distances (in base pairs) between TUBG-associated peaks and annotated centromeres in U2OS cells: either asynchronous (GSE173732_TUBG_NS_U2OS), synchronized in G0/G1 (GSE148798_gTub_G0_G1), early S phase (0 h; GSE148798_gTub_0h), or after release for 3

h (GSE148798_gTub_3h, GSE173732_TUBG_3h_U2OS), 5 h (GSE148798_gTub_5h, S phase), 7 h (GSE148798_gTub_7h, GSE173732_TUBG_7h_U2OS), or 9 h (GSE148798_gTub_9h and GSE173732_TUBG_9h_U2OS, S–G2/M phase) [4,24]. A random distribution of genomic regions, 200 bp in length with a standard deviation of 20 bp, was used as a control. This analysis shows that TUBG-associated peaks occur more frequently near annotated centromeres than expected from the random genomic regions, supporting a non-random centromere-proximal association.

d) Representative Airyscan 2 images of U2OS cells immunofluorescently co-stained with anti-TUBG (green) and anti-centromere (magenta), with DNA counterstained using Hoechst. To maintain visual clarity, panels display partial maximum-intensity projections (MIPs) of Z-stacks (acquired at 0.1 μm intervals). Yellow arrowheads indicate centrosomes; white boxes delineate magnified insets. TUBG signal is observed in close spatial association with centromere-defined structures. 3D surface rendering visualizes the spatial relationship between centromeres and γ-strings from two rotation angles. Colocalization pixel maps (CPMs) highlight spatial overlap in white. Scale bars: 10 μm. Images are representative of four independent experiments.

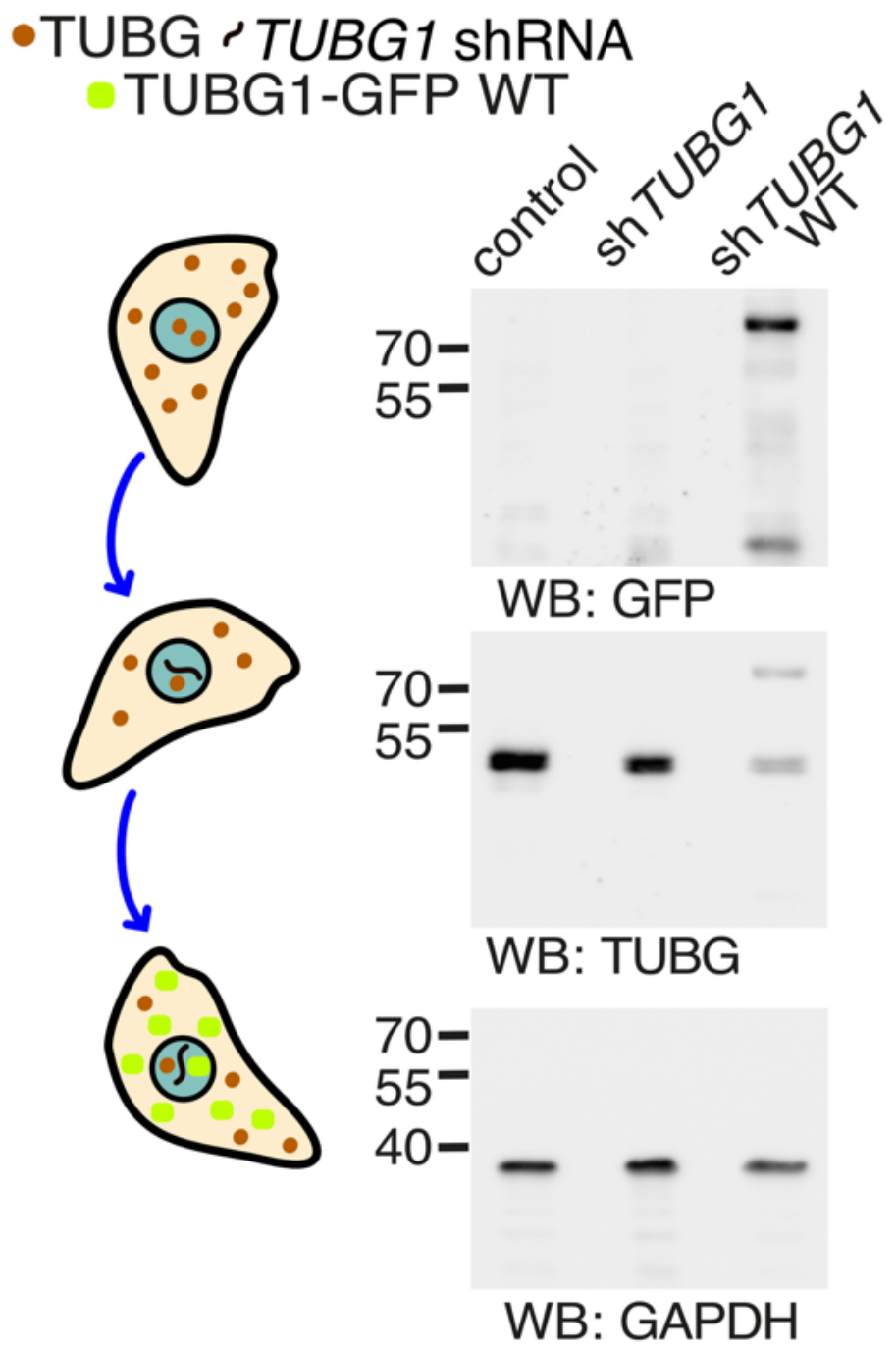

Supplementary Fig. 2 Validation of TUBG1 knockdown and rescue in U2OS cells. Western blot (WB) analysis of total cell lysates from control U2OS cells, U2OS cells stably expressing *TUBG1*-shRNA (sh), and cells co-expressing *TUBG1*-shRNA with an shRNA-resistant GFP-tagged wild-type TUBG1 construct (WT). Immunoblotting with anti-TUBG and anti-GFP antibodies confirms the partial depletion of endogenous TUBG and the stable expression of the GFP-tagged rescue protein, respectively. GAPDH was employed as a loading control (n = 3 independent experiments). A schematic detailing the genetic modifications of the cell lines is provided for additional context.

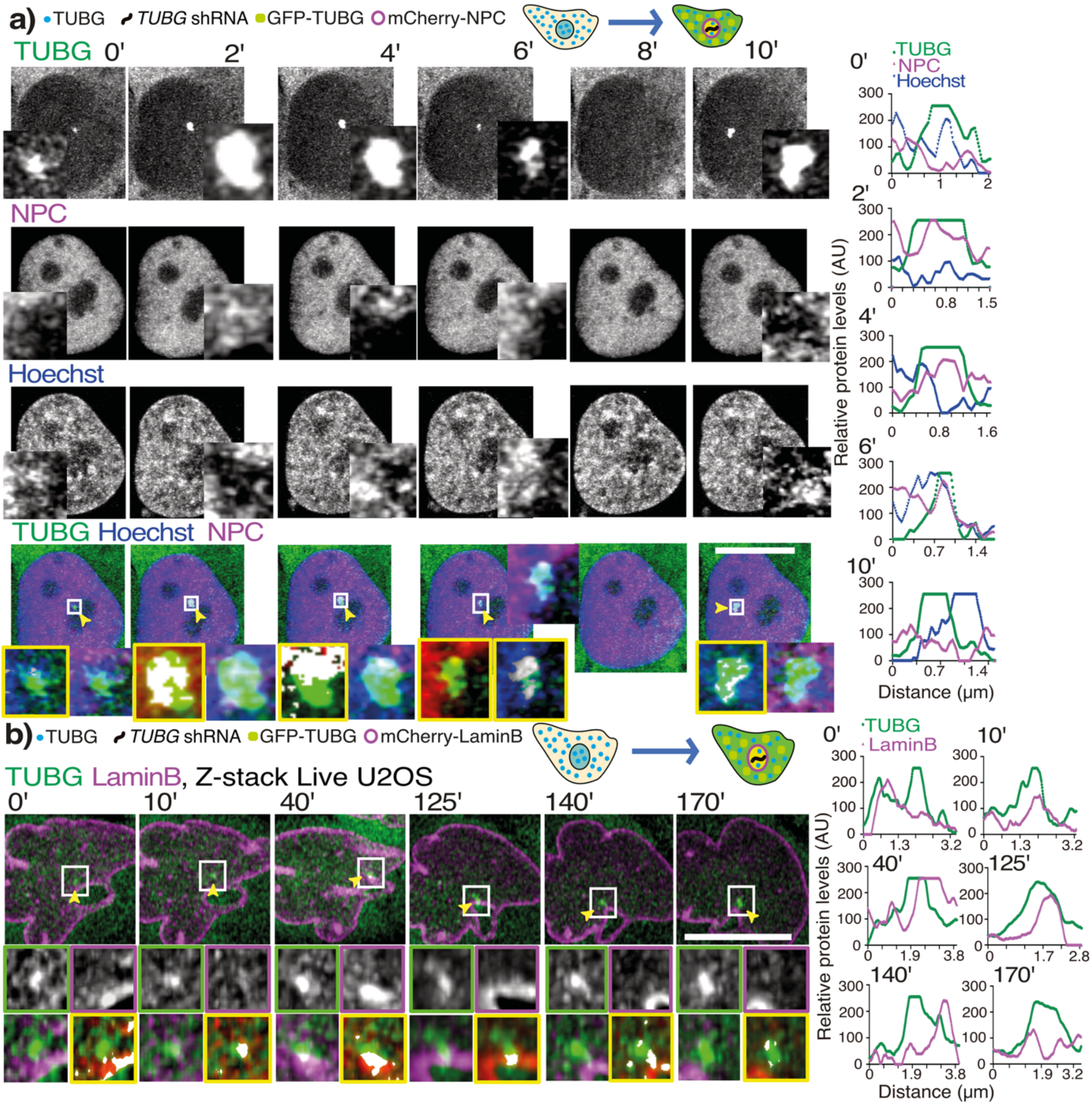

Supplementary Fig. 3 Interactions of centrosomes with chromatin, nuclear pore complexes, and nuclear lamina in live cells.

a) Time-lapse fluorescence images acquired with Airyscan 2, showing selected frames from a series recorded in U2OS cells stably co-expressing *TUBG* shRNA and sh-resistant TUBG-GFP, along with mCherry-NUP50-N10 (marking the nuclear-facing side of the nuclear pore complex). Images were acquired with Airyscan 2 as single Z-slices (0.1-μm thick) to monitor coplanar spatial associations. Selected frames at 6, 8, and 10 minutes reveal centrosome movement in and out of the imaging plane. Colocalization pixel maps (CPMs) display spatial overlap between TUBG and NUP50-N10 (2, 4 and 6 minutes) or TUBG and DNA (0, 6 and 10 minutes) within the magnified white box, consistent with centrosome movement near the nuclear region. Representative line plots (right) across the ROI insets show spatially coincident fluorescence intensity peaks for TUBG (green), NUP50 (red), and DNA (blue), supporting close spatial proximity of centrosomal TUBG to nuclear components within the analyzed focal plane.

b) Time-lapse frames, acquired with Airyscan, depicting transient spatial associations between the pericentriolar matrix (TUBG-GFP) and the nuclear envelope (Lamin B-mCherry). Images

were acquired as a Z-stack series (0.1-µm intervals) and displayed as maximum intensity projections (MIPs) to capture the full 3D volume of the centrosome-nuclear interface. CPMs show spatial overlap between the Lamin B and TUBG channels. Line plots (right) quantify these transient association events, showing spatial overlap between the pericentriolar TUBG signal and the nuclear lamina boundary.

a, **b**) A schematic representation of the modifications made to the cell lines is included for additional context. Yellow arrowheads denote the centrosome, while the yellow insets display colocalization pixel maps (CPMs), where white areas indicate colocalized pixels across channels. Scale bars, 10 µm.

Supplementary Video 1: Centrosomal TUBG and centromere dynamics
Description: Time-lapse sequence illustrating the dynamic movement of centrosomal TUBG (green) in close spatial association with centromeres (magenta) at the nuclear surface.
Cell line: Live U2OS cells stably expressing TUBG shRNA, sh-resistant TUBG-GFP, and mCherry-CENP-A (a centromere marker).
Imaging: Confocal microscopy. Time-lapse Z-stack images were acquired at 1-minute intervals (with 1-µm intervals between Z-planes) and presented as maximum intensity projections.
Related to: Fig. 4a

Supplementary Video 2: Centromere separation associated with TUBG
Description: Time-lapse sequence showing a close spatial association between centrosomal TUBG (green) and a single centromeric focus (magenta), followed by the apparent separation of the centromeric signal into two independent foci.
Cell line: Live U2OS cells stably expressing TUBG shRNA, sh-resistant TUBG-GFP, and mCherry-CENP-A (a centromere marker).
Imaging: Confocal microscopy. Time-lapse Z-stack images were acquired at 1-minute intervals (with 1-µm intervals between Z-planes) and presented as maximum intensity projections.
Related to: Fig. 4b

Supplementary Video 3: Gamma-tubule formation from nuclear envelope
Description: Transmitted photomultiplier tube (T-PMT)/fluorescence time-lapse images showing the formation of a γ-tubule (TUBG, green) extending from the nuclear envelope toward CENP-A-labeled centromeric foci (magenta).
Cell line: Live U2OS cells stably expressing TUBG shRNA, sh-resistant TUBG-GFP, and mCherry-CENP-A (a centromere marker).
Imaging: Confocal microscopy. Time-lapse Z-stack images were acquired at 1-minute intervals (with 1-µm intervals between Z-planes) and presented as maximum intensity projections.
Related to: Fig. 4c

Supplementary Video 4: Non-phosphorylatable TUBG-GFP Ser131Ala Foci
Description: Time-lapse series showing the transient formation of non-phosphorylatable TUBG-GFP Ser131Ala foci (green).
Cell line: shTUBG-TUBG-CENP-A-U2OS cells (stably expressing *TUBG* ShRNA to reduce endogenous TUBG, and mCherry-CENP-A as a centromere marker) transiently expressing TUBG-GFP Ser131Ala.
Treatment: Cells were treated with 5 µM cisplatin. Live-cell imaging was performed during cisplatin incubation.

Imaging: Time-lapse Z-stack images were acquired at 2.5-minute intervals (with 1-µm intervals between Z-planes) and displayed as maximum intensity projections.
Related to: Fig. 6a

Supplementary Video 5: Phospho-mimetic TUBG-GFP Ser131Asp foci and γ-tubules
Description: Time-lapse sequence illustrating the dynamic formation of phospho-mimetic TUBG-GFP Ser131Asp foci (green) and TUBG-GFP Ser131Asp-labeled γ-tubules that subsequently become spatially associated with the mCherry-tagged CENP-A centromere marker (magenta).
Cell line: ShTUBG-TUBG-CENP-A-U2OS cells (stably expressing *TUBG* ShRNA to reduce endogenous TUBG, and mCherry-CENP-A as a centromere marker) transiently expressing phospho-mimetic TUBG-GFP Ser131Asp.
Treatment: Cells were treated with 5 µM cisplatin. Live-cell imaging was performed during cisplatin incubation.
Imaging: Time-lapse Z-stack images were acquired at 2.5-minute intervals (with 1-µm intervals between Z-planes) and displayed as maximum intensity projections.
Related to: Fig. 6b

Supplementary Video 6: Centromere Movement with Phospho-Mimetic TUBG Foci
Description: This time-lapse series shows the dynamic formation of two TUBG-GFP Ser131Asp-labeled foci, with which centromeres become spatially associated and subsequently display coordinated movement.
Cell line: shTUBG-TUBG-CENP-A-U2OS cells (stably expressing *TUBG* ShRNA to reduce endogenous TUBG, and mCherry-CENP-A as a centromere marker) transiently expressing phospho-mimetic TUBG-GFP Ser131Asp.
Treatment: Cells were treated with 5 µM cisplatin. Live-cell imaging was performed during cisplatin incubation.
Imaging: Time-lapse Z-stack images were acquired at 2.5-minute intervals (with 1-µm intervals between Z-planes) and displayed as maximum intensity projections.
Related to: Fig. 6c

Supplementary Video 7: TUBG enrichment in micronuclei
Description: Z-stack time-lapse frames (with 1-µm intervals between Z-planes), acquired with Airyscan, depicting a micronucleus containing a TUBG-focus positioned at its nuclear envelope (Lamin B-mCherry). This is followed by TUBG enrichment at the nuclear envelope, leading to the accumulation of TUBG inside the micronucleus.
Cell line: Stable TUBGsh-U2OS cells (expressing *TUBG* ShRNA to reduce endogenous TUBG) coexpressing shRNA-resistant TUBG-GFP (green) and Lamin B-mCherry (a nuclear envelope marker; magenta).
Imaging: Time-lapse Z-stack images were acquired at 5-minute intervals and displayed as maximum intensity projections.
Related to: Fig. 9a

Supplementary Video 8: TUBG, PCNA-Chromobody, and DNA-associated dynamics in micronuclei
Description: Time-lapse Z-stack images illustrating micronuclear accumulation of TUBG concurrent with PCNA-Chromobody signal (Pcc-RFP; magenta) and increased DNA signal (Hoechst fluorescence) within the micronucleus. DNA was stained with Hoechst. These observations show temporal association among the three signals but do not establish that TUBG directly regulates DNA replication or micronuclear function.

Cell line: Stable TUBGsh-U2OS cells (expressing *TUBG* ShRNA to reduce endogenous TUBG) coexpressing shRNA-resistant TUBG-GFP (green) and PCNA-Chromobody.
Imaging: Time-lapse Z-stack images were acquired at 5-minute intervals (with 1-μm intervals between Z-planes) and displayed as maximum intensity projections.
Related to: Fig. 9b
