# Supplementary material for: The TUBG meshwork is associated with centromere dynamics and micronuclear organization
Source: Commun Biol. 2026 Sep 16;9:1206. doi: 10.1038/s42003-026-10880-y (PMC13582872; doi:10.1038/s42003-026-10880-y)
Supplement: Supplementary file 4 [file 42003_2026_10880_MOESM3_ESM.pdf]

Description of Additional Supplementary Files

File name: Supplementary Data
Description: Numerical source data underlying the graphs in Figs. 1–3 and 5–9 and Supplementary Figs. 1 and 3, together with original western blot data for Fig. 7h and Supplementary Fig. 2.

File name: Supplementary Video 1
Description: Supplementary Video 1: Time-lapse imaging of centrosomal TUBG in close spatial association with CENP-A-labelled centromeres at the nuclear surface. Related to Fig. 4a.
Supplementary Video 2: Time-lapse imaging of centrosomal TUBG associated with a centromeric focus followed by apparent separation of the centromeric signal into two foci. Related to Fig. 4b.
Supplementary Video 3: Time-lapse imaging of γ-tubule formation from the nuclear envelope toward CENP-A-labelled centromeric foci. Related to Fig. 4c.
Supplementary Video 4: Time-lapse imaging of transient TUBG-GFP Ser131Ala focus formation during cisplatin treatment. Related to Fig. 6a.
Supplementary Video 5: Time-lapse imaging of phospho-mimetic TUBG-GFP Ser131Asp foci and γ-tubules during cisplatin treatment. Related to Fig. 6b.
Supplementary Video 6: Time-lapse imaging of phospho-mimetic TUBG-GFP Ser131Asp foci spatially associated with centromeres and their coordinated movement. Related to Fig. 6c.
Supplementary Video 7: Time-lapse imaging showing TUBG enrichment at the micronuclear envelope followed by accumulation within the micronucleus. Related to Fig. 9a.
Supplementary Video 8: Time-lapse imaging showing micronuclear accumulation of TUBG together with PCNA-Chromobody and DNA-associated fluorescence. Related to Fig. 9b.
